# Safety and efficacy of cisplatin and nab-paclitaxel pressurised intraperitoneal aerosol chemotherapy (PIPAC) in patients with peritoneal carcinomatosis: Nab-PIPAC phase 1b study

**DOI:** 10.1016/j.ebiom.2026.106477

**Published:** 2026-09-11

**Authors:** Manuela Undurraga, Noemie Lang, Thibaud Koessler, Antonella Diciolla, Mariagrazia Di Marco, Nicolas Mach, Catherine Raimond, Vassilis Genoud, Valentine Du Bois, Jean-Christophe Tille, Aurelie Bornand, Jonathan S. Moore, Marc Weiner, Laure Smekens, José L. Sandoval, Julie Terzic, Yann Christinat, Jennifer Veillard, Petros Tsantoulis, Paul Thoueille, Thomas Mercier, Stefan Monig, Patrick Petignat, Christian Toso, Laurent Decosterd, Martin Hübner, Intidhar Labidi-Galy, Frederic Ris

**Affiliations:** a Division of Gynecology, Department of Paediatrics, Gynecology and Obstetrics, Geneva University Hospitals and Faculty of Medicine, University of Geneva, Geneva, Switzerland; b Department of Oncology, Geneva University Hospitals and Department of Medicine, Center of Translational Research in Onco-Haematology, Faculty of Medicine, University of Geneva, Geneva, Switzerland; c Department of Oncology, Lausanne University Hospital CHUV, Lausanne, Switzerland; d Division of Clinical Pathology, Department of Diagnostics, Geneva University Hospitals, Geneva, Switzerland; e Service and Laboratory of Clinical Pharmacology, Department of Laboratory Medicine and Pathology, Lausanne University Hospital CHUV and University of Lausanne, Lausanne, Switzerland; f Division of Visceral Surgery, Geneva University Hospitals, Geneva, Switzerland; g Department of Visceral Surgery, University Hospital CHUV and University of Lausanne (UNIL), Lausanne, Switzerland

**Keywords:** PIPAC, Nab-paclitaxel, Cisplatin, Peritoneal carcinomatosis, Ovarian, Gastric

## Abstract

**Background:**

Platinum and taxanes are backbone chemotherapy in multiple solid tumours. We aimed to investigate the safety, tolerability, and pharmacokinetics of nab-paclitaxel and cisplatin administered by pressurised intraperitoneal aerosol chemotherapy (PIPAC).

**Methods:**

Nab-PIPAC is a phase 1b trial investigating PIPAC administration of cisplatin at 10.5 mg/m^2^ and escalating dose of nab-paclitaxel with a 3 + 3 design (7.5, 15, 25, 37.5, 52.5 and 70 mg/m^2^). PIPAC was repeated at 4–6 weeks intervals for up to three courses to estimate the maximum tolerated dose (MTD).

**Findings:**

We enrolled 22 patients with peritoneal carcinomatosis, of whom 18 received at least one PIPAC: 6 ovarian, 9 gastric and 3 pancreatic cancers. Four patients (ovarian cancer) did not receive PIPAC due to bowel obstruction (n = 1) or no access to peritoneal cavity (n = 3). Median age was 67 years (43–81). In total, 45 PIPAC procedures were administered and 12 of 18 (67%) patients completed three PIPAC cycles. There was no dose limiting toxicity. The most common side effect was abdominal pain. One patient had grade 3 treatment related adverse event: bladder lesion requiring surgical reintervention. Median progression-free survival was 3.5 months and median overall survival was 9.4 months. Five patients with gastrointestinal cancer survived more than one year (3 gastric and 2 pancreatic). Pharmacokinetic analyses showed good linearity between nab-paclitaxel dose and the AUC_0-last_ (R^2^ = 0.90).

**Interpretation:**

The combination of nab-paclitaxel and cisplatin administered by PIPAC was safe and well tolerated in patients with peritoneal carcinomatosis. Further investigation of this combination is warranted.

**Funding:**

The study was supported by la bourse jeune chercheur des Hopitaux Universitaires de Geneve (NL), Fondation Fond’action (ILG) and Fondation NOVA (ILG).

**Research in context**

**Evidence before this study**

Pressurised intraperitoneal aerosol chemotherapy (PIPAC) is a technique developed in the last 15 years for the administration of low-dose chemotherapy during a laparoscopic procedure at an increase of intrabdominal pressure. The advantages of PIPAC are to decrease peri-operative morbidity while increasing the distribution and depth of drug penetration into peritoneal carcinomatosis nodules. One previous phase 1 trial investigated the safety and maximum-tolerated dose of nab-paclitaxel administered by PIPAC, but the investigators combined PIPAC with intravenous chemotherapy, preventing specific investigation of tolerance of PIPAC alone. Further, no study so far combined nab-paclitaxel with another drug by PIPAC. We have interrogated PubMed using the search strings ‘PIPAC’, “cisplatin” and ‘nab-paclitaxel’, until 11/06/2026. No previous clinical studies were retrieved that reported the combination of nab-paclitaxel and cisplatin during PIPAC.

**Added value of this study**

We conducted a phase 1b trial with dose escalation of nab-paclitaxel administered by PIPAC combined with flat dose of cisplatin at 10.5 mg/m^2^. We found that Nab-PIPAC was well tolerated with no dose limiting toxicity at dose level 70 mg/m^2^. The most common side effect was abdominal pain. One patient had grade 3 treatment related adverse event: bladder lesion requiring surgical reintervention. Five patients with gastrointestinal cancer survived more than one year (3 gastric and 2 pancreatic).

**Implications of all the available evidence**

The results of this trial show that combining nab-paclitaxel and cisplatin by PIPAC in patients with peritoneal carcinomatosis was safe and well-tolerated.

## Introduction

Peritoneal carcinomatosis (PC) is a major clinical challenge in gastrointestinal and gynaecological malignancies,^1^ occurring in up to 70% of ovarian cancer, 50% of gastric cancer and 10% of colorectal cancer. Patients with PC have a poor quality of life due to abdominal pain, loss of weight and refractory malignant ascites. Complications such as bowel occlusion and spontaneous bacterial peritonitis are the most frequent cause of death. Compared to other metastatic sites, patients with gastrointestinal cancer and PC had worse outcome.^1^, ^2^, ^3^ Recent multi-omics studies brought new insights into the biology of PC,^4^, ^5^, ^6^, ^7^ showing that it has a unique tumour immune microenvironment characterised by an enrichment in cancer-associated fibroblasts and reduced T cells, contributing to poor response to systemic chemotherapy.^4^

Due to limited blood supply to the peritoneum^8^ and hindered penetration of systemic drugs to the peritoneum, systemic anti-cancer therapies have limited effect on PC.^3^ Since PC is considered as locoregional disease for some cancers, particularly ovarian and appendiceal, due to direct shedding of tumour cells into the peritoneal cavity, multiple approaches of intra-peritoneal (IP) chemotherapy have been investigated. Pressurised intraperitoneal aerosol chemotherapy (PIPAC) is a technique developed in the last 15 years for the administration of low-dose chemotherapy during a laparoscopic procedure at an increase of intrabdominal pressure.^9^^,^^10^ The advantages of PIPAC are decreased peri-operative morbidity while increasing the distribution and depth of penetration drug into PC nodules.

There are practical and technical considerations for conducting phase 1 trials with PIPAC.^11^ It needs a strong multidisciplinary collaboration between surgeons and oncologists. To date, very few phase 1 clinical trials with dose-escalation of chemotherapy drug uniquely administered through PIPAC have been published^12^, ^13^, ^14^ and/or investigated the pharmacokinetics.^12^^,^^15^^,^^16^ The most investigated drugs by PIPAC are doxorubicin, cisplatin and oxaliplatin. Taxanes and platinum are essential drugs in the management of ovarian, gastric and pancreatic cancer. Nab-paclitaxel is a nanoparticle albumin-bound formulation of paclitaxel that has shown better toxicity profile and higher tumour control rate than paclitaxel in pre-clinical studies and early clinical trials (review in^17^). Few studies investigated the IP administration^18^ or PIPAC of nab-paclitaxel,^15^ and none combined it with another drug by PIPAC.

The primary objective of the current study was to assess the safety and tolerability of PIPAC administration of cisplatin and nab-paclitaxel, and determine the maximum-tolerated dose of nab-paclitaxel and flat-dose cisplatin. Secondary objectives were to report pharmacokinetic analysis of total plasma concentrations of paclitaxel, evaluate histological regression and objective tumour response rate assessed according to peritoneal regression grade score system (PRGS), assess the objective response rate and the clinical benefit rate, defined by revised RECIST v.1.1 criteria and the Quality of life.

## Methods

### Study design

This is a prospective, single arm, phase 1b multicentre trial, with a 3 + 3 dose-escalation design to evaluate the safety and tolerability of cisplatin and nab-paclitaxel in patients with PC. Rationale and study design of the protocol have been previously described in details.^19^ Briefly, patients were enrolled if they had PC related to pancreatic, oesogastric, ovarian cancer or primitive peritoneal mesothelioma, with limited extra-peritoneal metastases, who received at least one line of chemotherapy and for whom standard therapies have been exhausted or not feasible. Patients with residual disease following the first line of therapy or following secondary debulking were also eligible. Patients were required to have performance score 0–2, not candidate for surgical cytoreduction and IP/hyperthermic intraperitoneal chemotherapy (HIPEC) based on expert multidisciplinary board. The trial protocol is available in the Supplementary materials. The study was sponsored by Hopitaux Universitaires de Geneve.

### PIPAC procedure

Patients received PIPAC during laparoscopic surgery. First the peritoneal cavity was explored and the peritoneal cancer index (PCI) was calculated.^20^ Then punch biopsies were collected with up to four biopsies formalin-fixed paraffin-embedded (FFPE) for clinical pathology estimation of peritoneal regression grading score (PRGS) and two biopsies were snap-frozen for further translational research. Next, patients received intraperitoneal chemotherapy through PIPAC procedure. Specifically, chemotherapy agents were administered through a nebuliser to a high-pressure injector (Accutron HP-D injector, Medtron AG) and a nebuliser (Capnopen ®) at ambient temperature with a maximal upstream pressure of 300 psi. Treatment was maintained for 30 min at a pressure of 12 mmHg, and repeated at 4–6 weeks intervals for a maximum of 3 PIPAC courses. Cisplatin was administrated at flat dose of 10.5 mg/m^2^ reconstituted in 150 mL of NaCl 0.9% and escalating dose of nab-paclitaxel was reconstituted in 150 mL of NaCl 0.9%. The same dose was administered at each PIPAC procedure in the same patient. Concomitant intravenous chemotherapy was not allowed during the enrolment in the trial. In total, six cohorts were planned at nab-paclitaxel doses of 7.5, 15, 25, 37.5, 52.5 and 70 mg/m^2^.

### Patient population

Inclusion criteria for the study were the following: patients aged >18 years, psychologically and physically able to follow the trial procedures and to give a written informed consent, suffering from PC, with limited extraperitoneal metastases from pancreatic, oesogastric, ovarian cancers or primitive peritoneal mesothelioma, for whom standard therapies have been exhausted, nor feasible. Patients with residual disease following the first line of therapy or following secondary debulking were eligible.^19^

Patients were excluded in case of predominant extraperitoneal metastases at the discretion of the study team after discussion at the multidisciplinary tumour-board, in the presence of bowel obstruction, active gastroduodenal ulcer or ongoing abdominal infection (bacterial, viral or fungal), if the patient had received chemotherapy or surgery in the 2 weeks prior of enrolment, in the presence of general or local (abdominal) contraindications for laparoscopic surgery, and in the presence of known allergy to cisplatin or other platinum-containing compounds or to compounds of similar chemical or biological composition of nab-paclitaxel. Sex data were self-reported by study participants.

### Histological response

Punch peritoneal biopsies were collected from firm, nodular or plaque-like nodular deposits with features judged as visually malignant by the surgeon. Confluent or elevated lesions, as well as the presence of neovascularisation and inflammation, were considered indirect signs of malignancy and served as target zones for biopsies. Conversely, white, flattened lesions were typically viewed as indicators of post-chemotherapy fibrosis; these areas were biopsied to confirm this benign assumption. Between 3 and 4 biopsies were collected at each PIPAC for clinical pathology, fixed in 4% paraformaldehyde and embedded in paraffin. Tissues were sectioned and stained with haematoxylin & eosin. The PRGS was determined by a GI pathologist (AB) who was blinded for the treatment dose.^21^ The maximal PRGS score among all biopsies collected per PIPAC procedure was selected.

### Study endpoints

The primary endpoint was to assess safety and tolerability of cisplatin and nab-paclitaxel administered by PIPAC and to determine the maximum tolerated dose (MTD) of nab-paclitaxel. The study was designed following a modified Fibonacci sequence. Three patients were treated at each dose level (DL) until the first DLT in the first cycle of treatment (defined as grade 3 or 4, CTCAE version 5.0) occurred. Details of the statistical design have been previously described.^19^ MTD was defined as the lowest DL at which ≥33% (≥2/6) of patients experience DLT.

DLT was defined as any of the following CTCAE 5.0 Grade 3 or 4 adverse event determined to be probably, possibly or definitely related to Nab-paclitaxel (Abraxane®) and/or cisplatin IP administration and assessed within 4 weeks after the first PIPAC treatment:

#### Haematologic DLTs

- Febrile neutropenia grade >3 for more than 7 days
- Platelet count decreased grade 3 or 4 for more than 7 days
- Thrombocytopenia requiring transfusion

#### Non-haematologic DLTs

- Any grade ≥3 non-haematological trial treatment-related AE Exception: non-clinically significant non-haematological laboratory findings
- Any treatment-related AE that leads to a delay of treatment in the start of cycle 2 of >14 days
- Abdominal pain grade ≥3 during more than 7 days and requiring opioids treatment. Pain was estimated with visual analogic scale (VAS). The highest VAS value of the day taken in bed was recorded in the eCRF.

Adverse events related to the primary tumour, such as progression of the disease, were not considered as DLTs.

Secondary endpoints were i) to evaluate histological regression assessed according to PRGS^22^; ii) to assess the objective response rate (ORR) and the clinical benefit rate (CBR), defined by revised RECIST version 1.1 criteria^23^; iii) to evaluate any benefit in quality of life assessed by EORTC QLQ-C30 v3.0 and visual analogic scale for pain questionnaires. Other endpoints of interest included pharmacokinetic analyses of total plasma concentrations of paclitaxel and genomic alterations.

### Pharmacokinetics

Per protocol, pharmacokinetic analysis of paclitaxel plasma concentrations after nab-paclitaxel (Abraxane®) administration was planned to be performed for the two first patients treated for each dose level escalation, meaning 12 patients for the 6 dose levels. Blood samples for pharmacokinetic (PK) studies were collected in EDTA tubes at predefined time points. One 6 mL tube was collected at each of the following time point: t0 (before PIPAC), 1, 4 and 24 h after the end of PIPAC (locoregional administration of nab-paclitaxel). Blood samples were centrifuged at 2000 *g* for 10 min at 4 °C to separate the plasma. Plasma samples were stored at −80 °C until analysis. Total plasma concentrations of paclitaxel were determined using high-performance liquid chromatography-tandem mass spectrometry (LC-MS/MS). Plasma samples (50 uL-aliquots) were subjected to protein precipitation with 150 uL of methanol (MeOH) containing the stable istotope-labeled internal standard [^2^H_5_]-paclitaxel. Samples were vortex-mixed for 5 s, sonicated for 30 s, and subsequently centrifuged at 20,810 *g* for 10 min at +4°C. The resulting supernatant was diluted 1:1 with ultrapure water and homogenized before being transferred to HPLC vials for LC-MS/MS analysis. Paclitaxel plasma concentrations were measured at each defined time point. Standard PK parameters were calculated using non-compartmental calculations in PKanalix® (2023R1, Lixoft SAS, a Simulations Plus company). In particular, the area under the concentration-time curves from time zero to the last measurable concentration (AUC_0-last_, in this case approximately 24 h) was calculated for each individual using the linear-up log-down rule. The correlation between the dose and the AUC_0-last_ was investigated. Data visualisation was performed with R (R version 4.4.1, R Development Core Team, http://www.r-project.org/).

### Statistics

Data are presented as median (minimum–maximum). Progression-free survival (PFS) and overall survival (OS) were estimated using the Kaplan–Meier method. PFS was defined as the time from inclusion in the trial to the date of progression or death from any cause. OS was defined as the time from inclusion in the trial to the date of death from any cause. Only patients who received at least one PIPAC were included in the survival analyses. All analyses were performed using R (R version 4.4.1, R Development Core Team, http://www.r-project.org/).

### Genomic analyses

DNA was extracted from FFPE tumour blocks that contained at least 15% of tumour cells. From each sample, tumour DNA was extracted and analysed by next-generation sequencing of a panel than 400 genes (NGS400v5 Agilent SureSelect XT HS; Illumina NextSeq 550Dx). Note that for patient NAB-HUG-08, the NGS failed. When at least 30% tumour content was available (n = 13 patients), copy-number alterations were computed using OncoScan FFPE Assay Kit (ThermoFisher Scientific®). Homologous recombination defects (HRD) score was estimated using the Geneva HRD test, corresponding to the normalised large-scale transition score (nLST), previously described.^24^^,^^25^ A tumour was considered HRD-positive if its nLST score was greater than 15.

### Ethics

The study protocol was approved by SwissMedic, local ethical committees of Geneva (CCER) and Vaud (CER-VD) cantons (BASEC ID 2018-01327). The trial was prospectively registered on ClinicalTrials.gov on 25.06.2019 (NCT04000906). It was conducted in accordance with the Declaration of Helsinki and Good Clinical Practice Guidelines. Written informed consent was provided by all patients before undergoing any study-specific procedures.

### Role of funders

This clinical trial was supported by la bourse jeune chercheur des Hopitaux Universitaires de Genève (NL), and grants from Fondation Fond’action and Fondation NOVA. The funder did not have any role in study design, data collection, analyses, interpretation or manuscript writing. Nab-paclitaxel (abraxane ®) was purchased from Celgene and cisplatin (cisplatin-Teva®) was purchased from Teva. The nebuliser Capnopen ® was purchased from Capnopen and the high-pressure injector (Accutron HP-D injector, Medtron AG) was supplied by the department of surgery of each university hospital (HUG and CHUV). Manufacturers did not provide any material, intellectual or financial support for the study and did not have any role in the design, execution, analysis or reporting of the trial.

## Results

### Patient characteristics

**Fig. 1:**
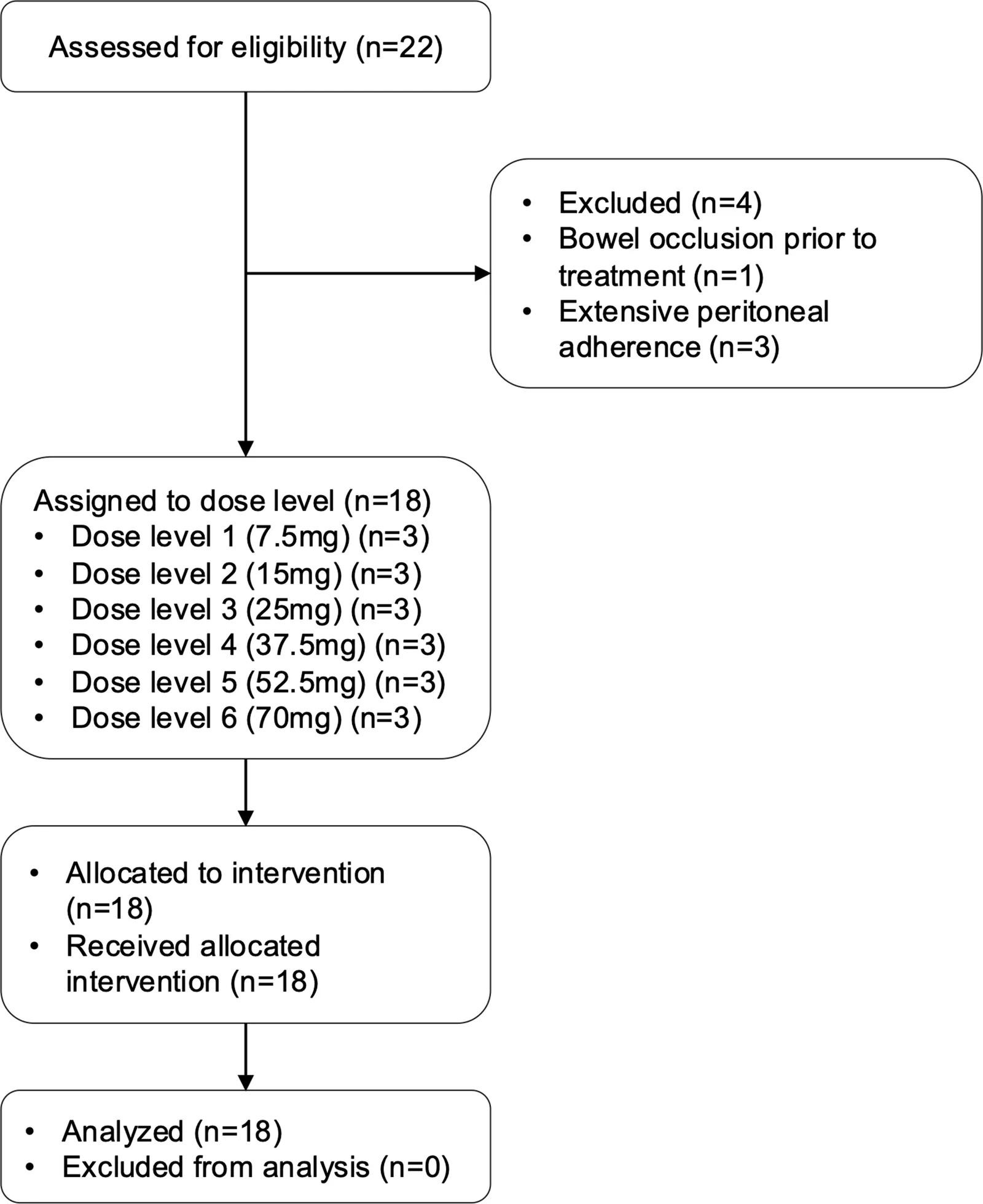
**CONSORT diagram**.

A total of 22 patients were enrolled between September 2021 and November 2023 at the Hôpitaux Universitaires de Genève (HUG) and Centre Hospitalier Universitaire Vaudois (CHUV), Switzerland. Four enrolled patients with platinum-resistant ovarian cancer were excluded from the study: three patients had extensive peritoneal adherence during laparoscopy, not allowing access to the peritoneal cavity and one patient (Patient NAB-HUG -12) developed bowel occlusion after signing the informed consent. The CONSORT diagram is detailed in Fig. 1. Overall, 18 patients received at least one PIPAC. Median age was 67 years (min–max: 43–81), and the majority (n = 13; 72%) were female. Six patients had high-grade serous ovarian carcinoma, nine had gastric adenocarcinoma and three had pancreatic adenocarcinoma (Table 1).

**Table 1:** Patient characteristics.

Variables	Total (n = 18)
Age (years)	67 (43–81)
Sex	
Female	13 (72%)
Male	5 (28%)
Origin of primary tumour	
High-grade serous ovarian carcinoma	6 (33%)
Oesogastric adenocarcinoma	9 (50%)
Pancreatic adenocarcinoma	3 (17%)
ECOG	
1	5 (28%)
0	13 (72%)
Previous lines of systemic therapy	
1	6 (33%)
2	7 (39%)
3+	5 (28%)
PCI score (median (min—max))	13 (4–39)
Ascites volume (mL)	
Absent	7 (39%)
Present	11 (61%)
Extraperitoneal metastasis	
Yes	3 (17%)
No	15 (83%)
Best response (according to RECIST v1.1)	
Progressive disease	7 (70%)
Stable disease	3 (30%)
Not evaluable	8

### DLT and maximum tolerated dose

Treatment related AE (TRAE) and severe adverse events (SAE) are summarised in Table 2. The most frequent reported adverse events were abdominal pain (seven patients) and fatigue (six patients). No DLT was observed in the six cohorts and MTD was not reached. Three patients developed SAE: one patient (Patient NAB-HUG-12) had bowel occlusion after signing the informed consent and was excluded from the study, one patient developed grade 2 renal insufficiency (Patient NAB-HUG-16) after the second PIPAC, and a third patient (Patient NAB-HUG-17) presented grade 3 abdominal pain due to bladder breach during the second PIPAC procedure (Clavien 3b surgical complication), leading to uroperitoneum that required repeat laparoscopic intervention 48 h later. This grade 3 adverse event was considered as TRAE. Of note, one grade 3 neutropenia, identified at the end-of-treatment visit two months after the last PIPAC cycle, was considered unrelated to study treatment.

**Table 2:** Adverse events.

Dose	7.5 mg/m^2^		15 mg/m^2^		25 mg/m^2^		37.5 mg/m^2^		52.5 mg/m^2^		70 mg/m^2^		Total
Grade	1–2	≥3	1–2	≥3	1–2	≥3	1–2	≥3	1–2	≥3	1–2	≥3	
Serious adverse events													
Abdominal pain	2		2		2					1			7
Blood creatinine increased					1		3						4
Treatment-related adverse events													
Decreased appetite					1								1
Diarrhoea					1				1				2
Fatigue	1	1			1		3						6
Memory impairment									1				1
Nausea and vomiting					1				1		1		3
Polyneuropathy											1		1
Scrotal infection											1		1
Skin rash			1		1								2
Weight decreased											1		1
Other adverse events													
Alopecia							1						1
Anaemia					1		1						2
Ascites	1						1				1		3
Back pain					1								1
Blood bilirubin increased									1				1
COVID-19 infection			1		2								3
Constipation							1		1				2
Cough	1												1
Eosinophilia			1										1
Haemorrhoids			1										1
Hypertension		1											1
Iridocyclitis					1								1
Neutropenia				1									1
Oedema peripheral							1						1
Oral candidiasis					1								1
Oropharyngeal pain	1												1
Pain in extremity					1								1
Pruritus							1						1
Respiratory infection			1										1
Rhinitis							1						1
Seroma	1												1
Venous thrombosis							1				1		2

### PIPAC procedures

In total, 45 PIPAC procedures were performed: 18 patients had one PIPAC, 15 patients had two PIPAC and 12 had three PIPAC (Table 3). After enrolment of 10 patients, an audit from Geneva ethical committee of (CCER) recommended that patients who did not progress after 3 PIPAC should pursue with additional PIPACs until progression. Two patients with gastric adenocarcinoma received additional PIPAC cycles after terminating the trial due to the favourable clinical response and tolerance: Patient NAB-HUG-13 received 2 additional PIPAC and Patient NAB-HUG-09 received one additional PIPAC. The median duration of hospitalisation was 3 days for PIPAC 1. Median duration of intervention was 138 min.

**Table 3:** Clinical and pathological outcome of PIPAC.

Variables	PIPAC C1 (n = 18)	PIPAC C2 (n = 15)	PIPAC C3 (n = 12)
Ascites volume (mL)	20 (0–5300)	20 (0–5000)	40 (0–3200)
PRGS score			
Grade 1	1 (6%)	3 (20%)	3 (25%)
Grade 2	7 (39%)	6 (40%)	4 (33%)
Grade 3	6 (33%)	6 (40%)	5 (42%)
Grade 4	2 (11%)	0	0
Not assessable	2 (11%)	0	0
PCI score	13 (4–39)	9 (2–39)	9.5 (0–39)
Operation time (minutes)	138 (117–306)	134 (114–311)	130.5 (112–234)
Duration of hospitalisation (days)	3 (2–4)	2 (2–4)	3 (2–3)

For ascites volume, operation time and duration of hospitalisation median and min–max values are displayed.

Overall, PIPAC procedures were well tolerated and had minimal impact on quality of life (Supplementary Fig. S1). EORTC QLQ-C30 scores were stable throughout treatment, with a median change of ≤−1 point from baseline for both Physical Functioning and Global QoL across all assessment time points. Peri-procedural pain, assessed by visual analogue scale (VAS, 0–10), peaked on the day of the procedure (median ∼3/10 for cycle 1, ∼2/10 for subsequent cycles) and resolved to baseline by Day +2, with VAS scores of 0 at follow-up across all cycles.

### Efficacy

About half of the patients (10/18; 55%) had measurable disease by RECIST at baseline and could be assessed for efficacy: three had stable disease, and seven had progressive disease at the end of treatment CT-scan (Table 1). The majority of patients (13/18) had a concurrent intra and extra-peritoneal progression (Table 1 and Supplementary Table S1). Multiple peritoneal biopsies were collected from at least 3 different regions of the abdominal cavity at each PIPAC before aerosol delivery. Among the 13 patients who received at least 2 PIPAC, 5 had improvement in PRGS, 5 had no changes and 3 had increase of PRGS (Supplementary Fig. S1A). Overall, there was high heterogeneity in PCI trajectories (Table 3 and Supplementary Fig. S1B).

**Fig. 2:**
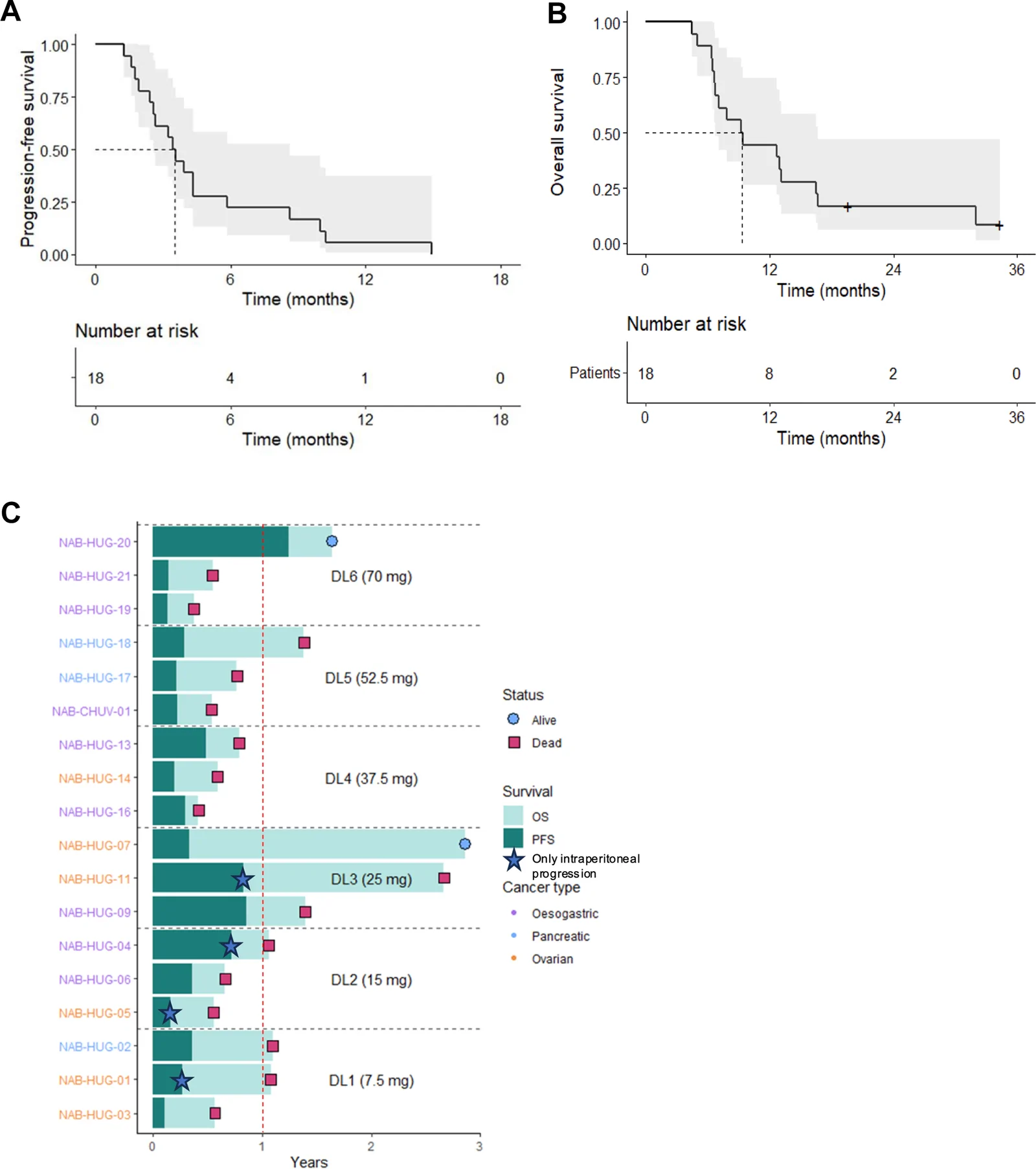
**Clinical outcomes. A.** Progression-free survival. **B.** Overall survival. **C.** Swimmer’s plot.

After a median follow-up of 9.3 months, median PFS was 3.5 (95% CI 2.6–8.6) months, and PFS rate at 6 months was 22.2% (95% CI 9.4–52.7 Fig. 2A). Median overall survival was 9.4 (95% CI 6.8–16.7) months, and OS rate at 12 months was 44.4% (95% CI 26.5–74.5; Fig. 2B). Five patients with gastrointestinal cancer were alive at one year: 3 patients with gastric cancer and two patients with pancreatic cancer. Responses over time by cancer of origin and dose level are illustrated in a swimmer plot Fig. 2C.

### Pharmacokinetics

**Fig. 3:**
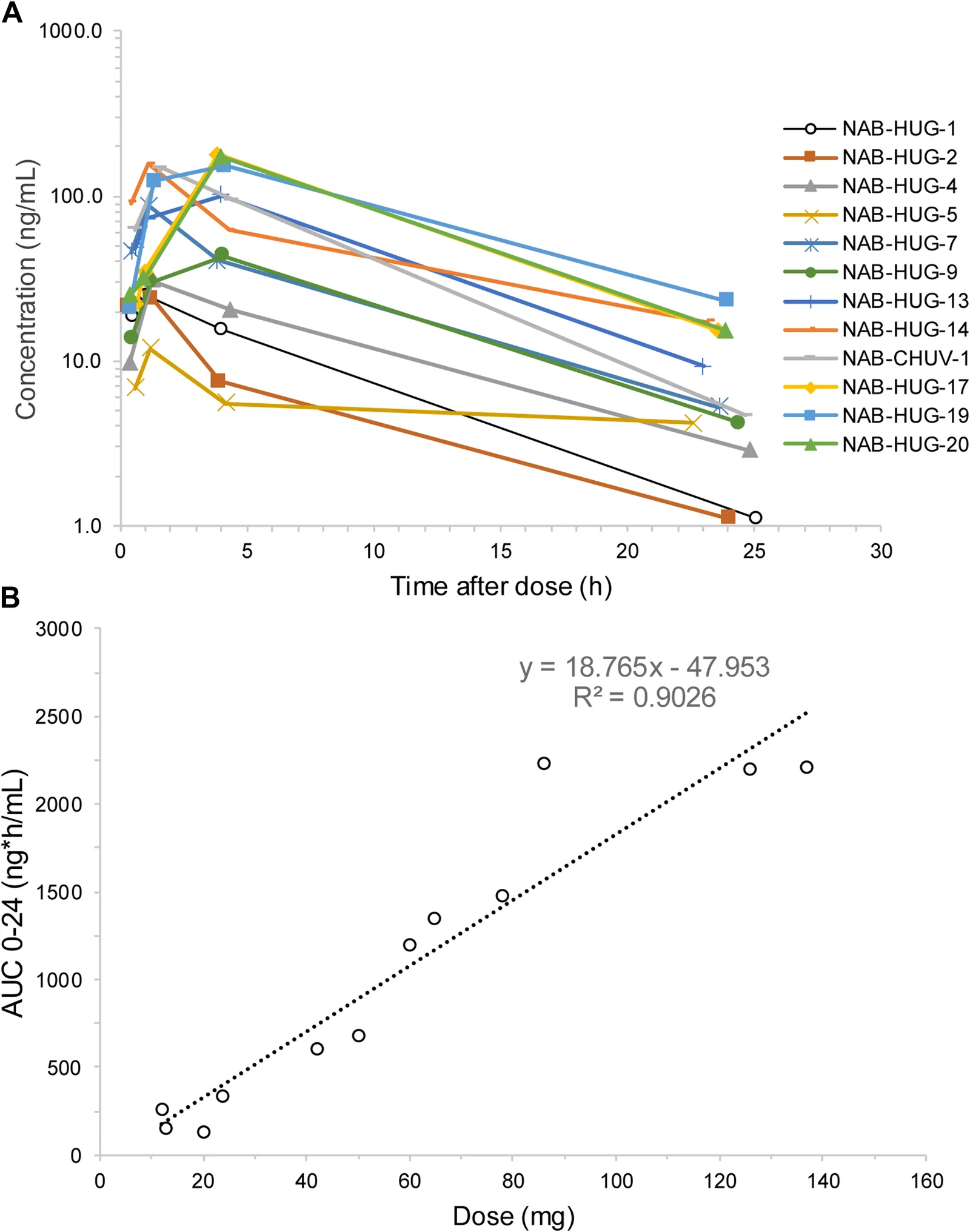
**Pharmacokinetics. A.** Individual concentration-time profiles. **B.** Correlation between nab-paclitaxel dose and AUC_0-last._ AUC was calculated using PKanalix (PKA1: linear/log).

PK analyses were performed in 12 patients. Table 4 shows the PK parameters calculated for each individual, each of whom received a different PIPAC dose. Fig. 3A illustrates the concentration-time profiles of paclitaxel for the various doses administered. The median time to reach maximum concentration (T_max_) was 87 min, with a range of 60–249 min, indicating significant variability in absorption rates across individuals. The median half-life of elimination (t_1/2_) was 6.0 (range: 4.6–46.2) hours. Of note, the longer t_1/2_ of 46.2 h calculated after administration of 20 mg of PIPAC in patient HUG-05, is related to the unusually flat PK profile, resulting in an increased calculated volume of distribution, which in turn resulted in a longer estimated t_1/2_. This difference becomes particularly apparent when examining the contribution of the AUC_0-last_ to the estimation of the AUC_0-inf_ (equations provided in Supplementary Material). For most individuals, the AUC_0-last_ accounted for a median of 92.7% (range: 84.9–97.2) of the AUC_0-inf_. However, in the individual with the extended t_1/2_, the contribution was reduced to 30.2%. PK analyses showed good linearity between nab-paclitaxel dose and AUC_0-last_ with a coefficient of determination (R^2^) of 0.90 (Fig. 3B).

**Table 4:** Individual pharmacokinetic parameters of nab-paclitaxel administered by PIPAC.

Patient ID	dose (mg/m^2^)	total dose	t_max (h)_	C_max__(ng/mL)_	AUC0_inf_	AUC0-tlast	t_1/2 (h)_	% diff	CL/F	Clast	kel	Vd/F
Nab_HUG_1	7.5	12	1.00	25.0	199	191	5.3	95.7%	0.06	1.11	0.13	0.47
Nab_HUG_2	7.5	13	1.23	23.8	141	129	7.3	91.8%	0.09	1.1	0.095	0.97
Nab_HUG_5	15	20	1.18	11.9	403	122	46.2	30.2%	0.05	4.21	0.015	3.32
Nab_HUG_4	15	24	1.27	30.8	309	280	7.0	90.7%	0.08	2.84	0.099	0.78
Nab_HUG_9	25	42	4.07	43.1	501	464	6.3	92.7%	0.08	4.2	0.11	0.73
Nab_HUG_7	25	50	1.13	87.8	608	563	5.8	92.6%	0.08	5.27	0.12	0.70
Nab_HUG_14	37.5	60	1.13	155.2	1280	1086	7.8	84.9%	0.05	17.12	0.089	0.53
Nab_HUG_13	37.5	65	4.00	101.5	1102	1029	5.3	93.4%	0.06	9.21	0.13	0.47
Nab_CHUV_1	52.5	78	1.62	147.7	1096	1065	4.6	97.2%	0.07	4.63	0.15	0.48
Nab_HUG_17	52.5	86	3.85	178.5	1756	1633	5.8	93.0%	0.05	15.29	0.12	0.39
Nab_HUG_19	70	126	4.15	151.7	2056	1814	7.3	88.2%	0.06	22.97	0.095	0.64
Nab_HUG_20	70	137	3.95	173.0	1751	1624	5.8	92.8%	0.08	15.36	0.12	0.64

t_max_:time to maximum concentration; C_max_:maximum concentration; AUC_inf_: area under the curve from time 0 to infinity; CL/F: clearance; t_1/2_: elimination half-time; Vd/F: volume of distribution.

### Molecular alterations

**Fig. 4:**
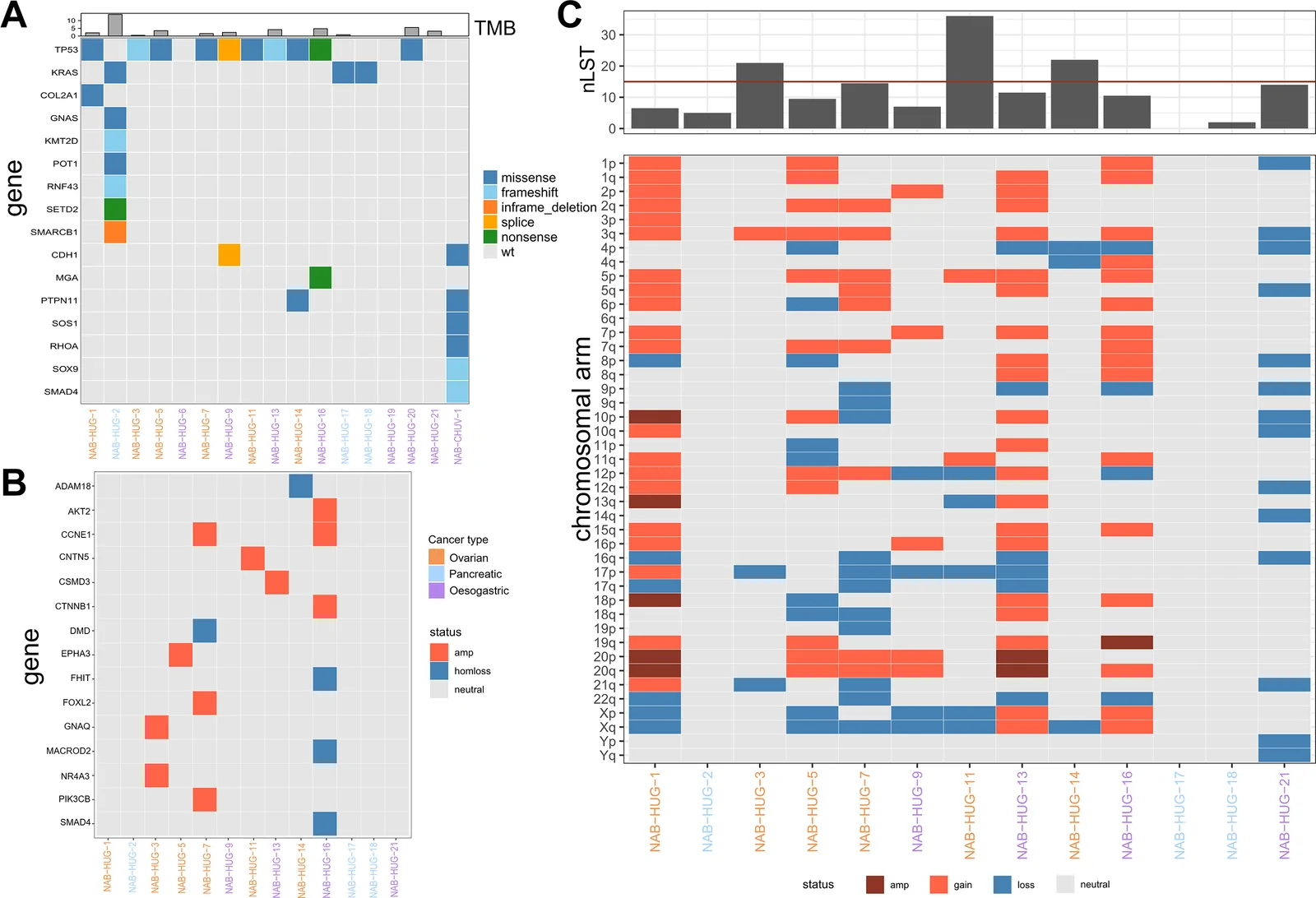
**Molecular alterations. A.** Pathogenic mutations and Tumour mutational burden (TMB). **B.** Gene amplification and homozygote losses. **C.** Whole chromosomal arms copy number variations and homologous recombination defects (HRD) score (nLST).

Molecular analyses were successful in 17 patients for NGS 400-genes and 13 patients for SNP arrays. They revealed that all high-grade serous ovarian carcinoma had *TP53* mutations, mostly missense (5/6; Fig. 4A), and high genomic instability (Fig. 4B and C), as expected, while gastro-intestinal carcinomas had heterogeneous alterations. The three patients with pancreatic adenocarcinoma had pathogenic *KRAS* mutations. Further, patient HUG-02 that had MSI-high pancreatic adenocarcinoma showed high TMB at 14, as expected. Genomic instability was high in most of the analysed tumours, as depicted in Fig. 4C. Three patients with ovarian cancer had HRD positive tumours. The two long-survivor ovarian cancer patients (H07 and H11), alive at 24 months, had HRD negative tumours. Two long-survivor gastric cancer patients (H09 and H20), alive at 12 months, harboured *TP53* mutation. These observations are limited by the number of patients per subgroup and are hypothesis generating.

## Discussion

Peritoneal carcinomatosis is a major clinical challenge in multiple gynaecological and gastrointestinal cancers, and is associated with poor outcomes. PIPAC emerged in the last decade as a palliative therapeutic option for PC, enabling repeated locoregional delivery of chemotherapy.^9^ However, very few clinical trials with dose-escalation of chemotherapy administered by PIPAC only have been conducted.

In the current study we report the safety and tolerability of a phase 1b trial investigating the administration of nab-paclitaxel and cisplatin by PIPAC.^19^ Overall, Nab-PIPAC was well tolerated. One grade 3 TRAE was recorded, a bladder breach (Clavien-Dindo 3b surgical complication) during the second PIPAC procedure in one patient, with no chemotherapy-related grade 3 toxicity observed. MTD was not reached with the caveat that the grade 3 TRAE was excluded from the DLT assessment on procedural grounds. Haematologic toxicity was moderate, not exceeding grade 2. In our study, most patients (87.5%) had metastases limited to the peritoneal cavity and all received at least one line of previous chemotherapy for metastatic disease. Two previous studies investigated IP administration of nab-paclitaxel. One phase 1 clinical trial investigated weekly IP administration of nab-paclitaxel.^18^ The NAB-PTX phase 1 trial explored nab-paclitaxel administration by PIPAC. However, the majority of patients enrolled in NAB-PTX received concomitant IV chemotherapy, not allowing specific investigation of safety and tolerability of nab-paclitaxel by PIPAC.^15^

The rationale for having low dose (7.5 mg/m^2^) at the first dose level of nab-paclitaxel was that PIPAC allows administration of very low dose of chemotherapy, up to 10-fold less than IV route with a high penetration of the drug into the tissue.^26^ In theory, this approach would allow a higher therapeutic ratio by decreasing systemic toxicities while increasing or maintaining local response. Interestingly, the two long-survivor ovarian cancer patients (alive more than 2 years after enrolment) received nab-paclitaxel at 25 mg/m^2^ (DL3). Beyond PIPAC, the benefit of low chemotherapy has been shown with oral drugs such as cyclophosphamide and capecitabine, administered through a “metronomic schedule”.^27^ Five patients with gastrointestinal cancer survived more than one year: 3 patients with gastric cancer and two patients with pancreatic cancer. This prolonged survival is notable in light of the dismal prognosis typically associated with synchronous PC, where median OS from diagnosis reported to be 5 months for pancreatic cancer^28^ and 10 months for gastric cancer.^29^

In terms of safety, DLT was defined as grade 3 or 4 CTCAE occurring the 4 weeks from the first cycle of PIPAC. If a SAE occurred after the second or third PIPAC, it was not considered as a DLT. We did not observe a DLT during the study and MTD was not reached with nab-paclitaxel at 70 mg/m^2^. This is consistent with another phase 1 trial with nab-paclitaxel administered by PIPAC, where the MTD 140 mg/m^2^.^15^ As no DLT was observed and MTD was not reached, the RP2D for future trials is 70 mg/m^2^ of nab-paclitaxel combined with cisplatin at 10.5 mg/m^2^. The linear dose–AUC relationship and low systemic exposure at this dose level provide pharmacokinetic support for its use in phase 2 evaluation. Importantly, we observed few severe adverse events, in accordance with previous reports of favourable tolerance of PIPAC, and only one grade 3 TRAE.

To better understand the limited systemic toxicity observed in our study, we compared our PIPAC PK parameters at 70 mg/m^2^ to those reported by Nyman et al.^30^ for intravenous (IV) nab-paclitaxel at a similar dose (80 mg/m^2^). The maximal concentration (Cmax) did not exceed 173 ng/mL with PIPAC, representing an approximately 17-fold reduction compared to the 3017 ng/mL reported for IV administration.^30^ Furthermore, overall systemic exposure was substantially reduced. In our cohort, the extrapolated area under the curve (AUC0-inf) at doses approximating 70 mg/m^2^ was roughly 1900 ng∗h/mL. This is lower than the IV exposures reported by Nyman et al., which range higher at comparable doses. Finally, regarding the elimination half-life (t1/2), the median of 6.0 h observed in our study is shorter than the terminal t1/2 of 15–18 h reported for IV administration.^30^ This is primarily a reflection of our 24-h sampling window, which captures the drug’s extensive tissue distribution phase rather than the full terminal elimination phase. Strikingly, this profound reduction in both peak and overall systemic exposure are highly consistent with the PK data reported by Ceelen et al. in the NAB-PTX trial.^15^ It is consistent with the locoregional delivery principle of PIPAC and provides a pharmacokinetic rationale for its favourable systemic safety profile.

### Caveats and limitations

Our study faced several challenges that are specific to local therapies of PC. PIPAC could not be performed in three of the 22 enrolled patients due to peritoneal adherences, all three having high-grade serous ovarian carcinoma. There was no measurable disease at baseline in 8 patients (45%) limiting radiological assessment of ORR. The median follow-up of 9.3 months was similar to the median OS of 9.4 months, reflecting the poor prognosis of this heavily pre-treated population with peritoneal carcinomatosis and indicating that most survival time is event-driven rather than censored. Consequently, longer-term survival estimates, such as the 12-month OS rate, carry wide confidence intervals and should be interpreted with caution. Notably, two patients with prolonged survival (approximately 3 years) contribute to the observed heterogeneity and the width of the confidence interval. As OS was a secondary exploratory endpoint in this phase 1b safety study, the limited follow-up does not affect the primary conclusions regarding safety and tolerability. The analyses of genomic alterations were limited by the number of patients per subgroup and are hypothesis generating. We observed few changes of PRGS after repeated cycles of PIPAC. It is important to note that we chose the PRGS score as the maximal one among the collected biopsies per PIPAC, rather than the mean value of biopsies collected in the 4 abdominal quadrants,^22^ which limits comparability with previous reports. We chose to collect the maximal PRGS because, from the biological perspective, drug-tolerant “persistent” tumour cells will drive tumour recurrence and progression.^31^ The numerous biopsies collected during this trial are a unique opportunity for a better comprehension of the biology and tumour microenvironment of PC resistant to therapy, and translational research on these biopsies is ongoing^32^ (not peer-reviewed).

In conclusion, intraperitoneal aerosolised nab-paclitaxel and cisplatin administered by PIPAC is safe and well-tolerated in patients with previously treated PC, and no MTD was reached. Further investigation of this combination in gastric and pancreatic cancer is warranted.

## Contributors

MU: conceptualisation, investigation, methodology, writing—original draft, and writing—review & editing; NL: conceptualisation, funding acquisition, methodology, writing—review & editing; TK: investigation, resources, writing—review & editing; AD: investigation, writing—review & editing; MDM: methodology, project administration, writing—review & editing; NM: project administration, resources, writing—review & editing; CR: data curation, project administration, methodology, writing—original draft; VG: data curation, formal analysis, software, validation, visualisation, writing—original draft; VDB: investigation, methodology, writing—review & editing; JCT: investigation, writing—review & editing; AB: investigation, writing—review & editing; JSM: investigation, writing—review & editing; MW: investigation, writing—review & editing; LS: investigation, writing—review & editing; JLS: data curation, formal analysis, software, validation, visualisation; JT: investigation, data curation, writing—review & editing; YC: data curation, formal analysis, software, validation, visualisation; JV: data curation, formal analysis, software, validation, visualisation; PTs: investigation, writing—review & editing; PTh: investigation, methodology, validation, visualisation, writing—review & editing; TM: investigation, methodology, validation, visualisation, writing—review & editing; SM: investigation, writing—review & editing; PP: investigation, funding acquisition, resources, writing—review & editing; CT: funding acquisition, resources, writing—review & editing, LD: investigation, methodology, validation, visualisation, writing—review & editing; MH: conceptualisation, investigation, methodology, writing—review & editing; ILG: conceptualisation, funding acquisition, investigation, methodology, resources, supervision, validation, writing—original draft; FR: conceptualisation, funding acquisition, investigation, methodology, resources.

Three authors have accessed and verified the underlying data (JV, JLS and ILG). All the authors read and approved the final version of the manuscript.

## Data sharing statement

The anonymised source clinical data from this study and the study protocol is available upon request from the co-last author (Intidhar.labidi-galy@hug.ch).

## Declaration of interests

MU: none; NL: funding (my institution):Hopitaux Universitaires de Geneve (bourse jeune chercheur); TK: consulting fees (my institution): GSK, TAKEDA, Pierre Fabre; Payment for expert testimony (my institution): Incyte; Support for attending meetings/travel (my institution): MERCK, ROCHE; AD: none; MDM: none; NM: none; CR: none; VG: none; VDB: none; JCT: none; AB: none; JSM: none; MW: none; LS: none; JLS: none; JT: none; YC: none; JV: none; PTs: consulting fees (my institution): NOVARTIS, ASTELLAS, AZ, MSD, BAYER, JANSSEN, BMS; support for attending meetings/travel (my institution): JANSSEN, BAYER; PTh: none; TM: none; SM: none; PP: none; CT: none; LD: none; MH: none; ILG: funding (my institution): foundation NOVA and foundation Fond’action; consulting fees (my institution): GSK, ABBVIE, MSD; Support for travelling/meetings (my institution): PHARMAMAR; Stock (myself): AGENUS, DAIICHI-SANKYO; FR: none.

## Appendix A Supplementary data

Supplementary Figs. S1 and S2

NabPIPAC_Protocol_V 5.0_FINAL
