# Supplementary material for: Safety and efficacy of cisplatin and nab-paclitaxel pressurised intraperitoneal aerosol chemotherapy (PIPAC) in patients with peritoneal carcinomatosis: Nab-PIPAC phase 1b study
Source: eBioMedicine. 2026 Sep 11;132:106477. doi: 10.1016/j.ebiom.2026.106477 (PMC13587052; doi:10.1016/j.ebiom.2026.106477)
Supplement: Supplementary file 1 [file mmc2.pdf]

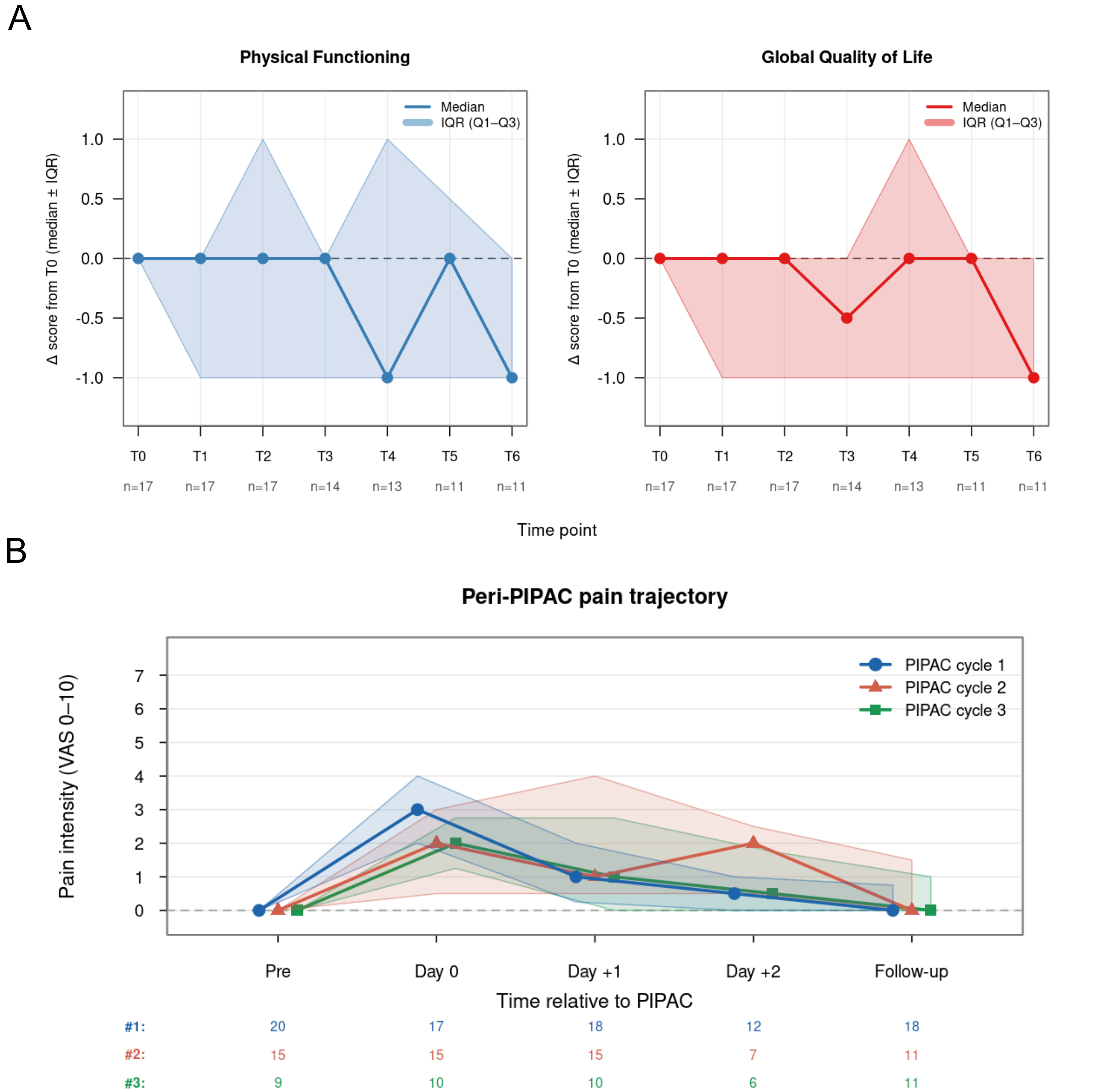

Supplementary Figure 1: Patient-reported quality of ife during PIPAC treatment. A. Patient-reported quality of life during PIPAC treatment**.** Physical Functioning (left panel) and Global Quality of Life (right panel) were assessed using the EORTC QLQ-C30 questionnaire the day before each PIPAC procedure (T0, T2 and T4), 10 days after a PIPAC procedure (T1, T3 and T5) and within 28 days of the last PIPAC (T6). Scores are expressed as change from individual baseline (T0). Each line represents the median change across evaluable patients; shaded areas indicate the interquartile range (IQR, Q1–Q3). The number of patients contributing at each time point is shown below the x-axis. A positive value indicates improvement; a negative value indicates deterioration relative to baseline. PIPAC, pressurised intraperitoneal aerosol chemotherapy; EORTC QLQ-C30, European Organisation for Research and Treatment of Cancer Quality of Life Questionnaire Core 30; IQR, interquartile range; T0, baseline assessment prior to first PIPAC procedure. **B.** Peri-procedural pain intensity by PIPAC cycle. Median VAS scores (0–10) are plotted at five time windows anchored to the procedure date: pre-procedure (days −3 to −1), day of procedure (day 0), day +1, day +2, and follow-up (days +7 to +11). Shaded areas represent interquartile ranges. Only time points with n ≥ 3 observations are shown. Numbers below the x-axis indicate the number of observations per cycle and time window.

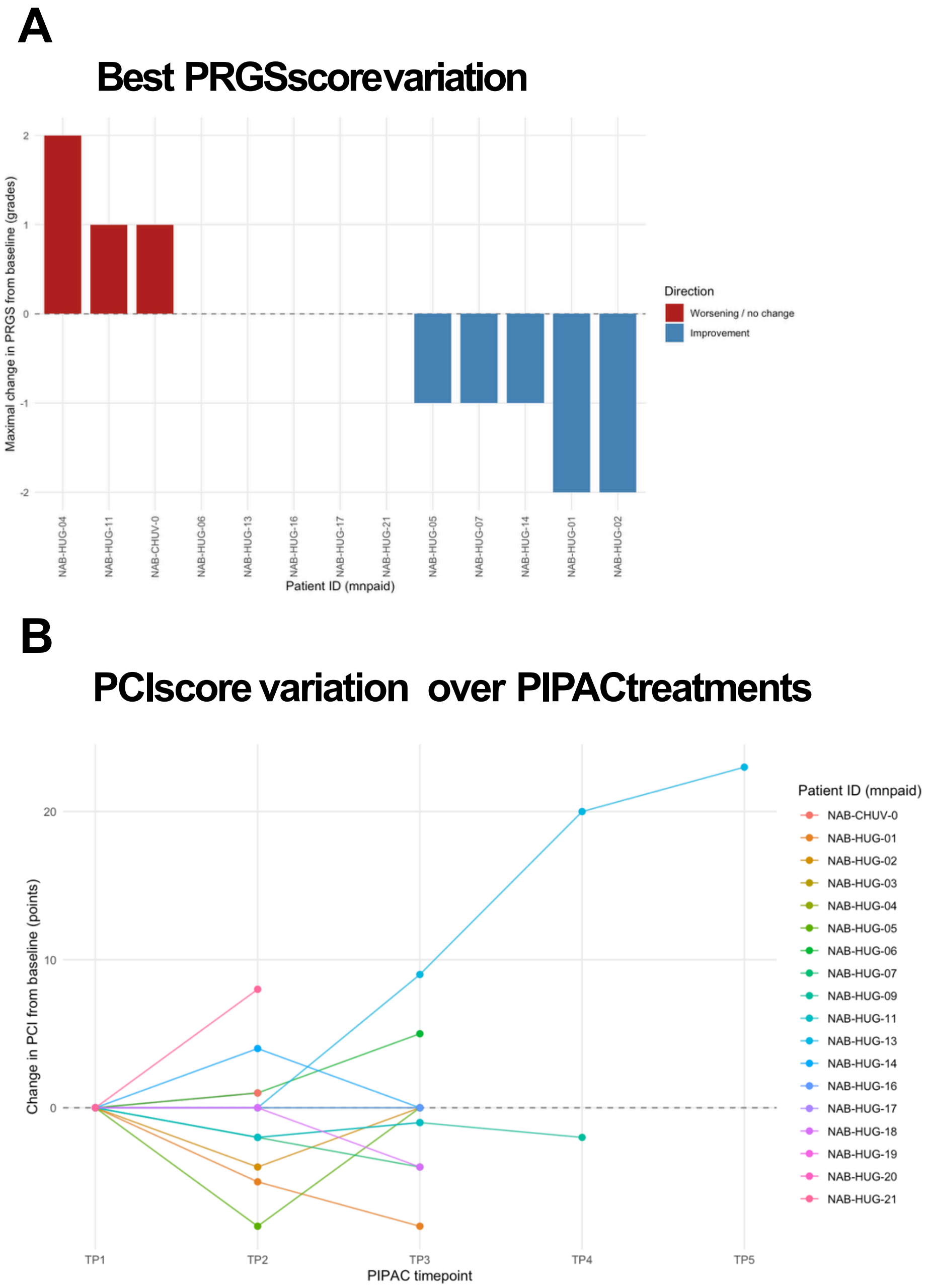

Supplementary Figure 2: PRGS and PCI evolution. A. Best PRGS score variation**. B.** PCI score variation over PIPAC treatments.
