# Supplementary material for: Safety and efficacy of cisplatin and nab-paclitaxel pressurised intraperitoneal aerosol chemotherapy (PIPAC) in patients with peritoneal carcinomatosis: Nab-PIPAC phase 1b study
Source: eBioMedicine. 2026 Sep 11;132:106477. doi: 10.1016/j.ebiom.2026.106477 (PMC13587052; doi:10.1016/j.ebiom.2026.106477)
Supplement: Supplementary file 2 [file mmc3.pdf]

Clinical Study Protocol

A PHASE IB TRIAL OF INTRAPERITONEAL CISPLATIN AND NAB-PACLITAXEL ADMINISTERED BY PRESSURIZED INTRAPERITONEAL AEROSOL CHEMOTHERAPY (PIPAC) IN THE TREATMENT OF ADVANCED MALIGNANCIES CONFINED TO THE PERITONEAL CAVITY

SHORT TITLE and / or trial acronym: NAB – PIPAC

Study Type:	Phase IB with Nab-paclitaxel and cisplatin administered intraperitoneally by PIPAC
Study Categorisation:	Risk category according to LHR : B
Study Registration:	www.Clinicaltrials.gov, number NCT04000906
Sponsor:	Hopitaux Universitaires de Genève
Principal Investigator<br>And Investigator coordinator:	Dr Intidhar Labidi-Galy<br>Departement of Oncology<br>Geneva University Hospitals (HUG)<br>Faculty of medicine, UNIGE<br>intidhar.labidi-galy@hcuge.ch<br>+41 79 55 34 976
Sponsor Investigator	Pr Nicolas Mach<br>Head of the DFDL Onco-Haematology Research Unit<br>Departement of Oncology<br>Geneva University Hospitals (HUG)<br>Faculty of medicine, UNIGE<br>Nicolas.Mach@hcuge.ch<br>0041223722901<br>Rue Gabrielle-Perret-Gentil 4<br>1205 Genève
Investigational Product:	Nab-paclitaxel (Abraxane®)
Protocol Version and Date:	V 5.0 – 17.01.2023

CONFIDENTIAL
The information contained in this document is confidential and the property of the Dr Intidhar Labidi-Galy and the HUG. The information may not - in full or in part - be transmitted, reproduced, published, or disclosed to others than the applicable Competent Ethics Committee(s) and Regulatory Authority (ies) without prior written authorisation from the sponsor except to the extent necessary to obtain informed consent from those who will participate in the study.

Signature Page(s)

Study number	2018 -01327
Study Title	A PHASE IB TRIAL OF INTRAPERITONEAL CISPLATIN AND NAB-PACLITAXEL ADMINISTERED BY PRESSURIZED INTRAPERITONEAL AEROSOL CHEMOTHERAPY (PIPAC) IN THE TREATMENT OF ADVANCED MALIGNANCIES CONFINED TO THE PERITONEAL CAVITY

The Sponsor-Investigator and trial biostatistician have approved the protocol version [V5.0 (dated 17.01.2023)], and confirm hereby to conduct the study according to the protocol, current version of the World Medical Association Declaration of Helsinki, ICH-GCP guidelines or ISO 14155 norm if applicable and the local legally applicable requirements.

Sponsor: Pr Nicolas Mach

____________________________ ____

Place/Date Signature

Local Principal Investigator at study site:

I have read and understood this trial protocol version [V5.0 (dated 17.01.2023)] and agree to conduct the trial as set out in this study protocol, the current version of the World Medical Association Declaration of Helsinki, ICH-GCP guidelines or ISO 14155 norm and the local legally applicable requirements.

I, the undersigned, am responsible for the conduct of the study at this site and agree to the following:

- I understand and will conduct the study according to the protocol, any approved protocol amendments, ICH-GCP and all applicable regulatory authority requirements and national laws.
- I will not deviate from the protocol without prior written permission from the Sponsor, except where necessary to prevent any immediate danger to the subject.
- I have been trained for the use of PIPAC and I am familiar with the Investigational Medicinal Product (Abraxane®) and its/their use according to this protocol.
- I have sufficient time to properly conduct and complete the trial within the agreed trial period, and I have available an adequate number of qualified staff and adequate facilities for the foreseen duration of the trial to conduct the trial properly and safely.
- I will ensure that any staffs at my site(s) who are involved in the study conduct are adequately trained regarding PIPAC, the protocol and their responsibilities. In the case of delegating any of my study responsibilities I will provide the Sponsor with a Delegation of Activities certificate.

**Site**	**HUG** – Rue Gabrielle Perret-Gentil 4, 1205 Genève
Principal investigator	Dr Intidhar Labidi-Galy

Place/Date Signature

**Site**	**CHUV** – Rue du Bugnon 21 CH 1011 Lausanne
Principal investigator	Dr Antonella Diciolla

Place/Date Signature

Table of Contents

STUDY SYNOPSIS **7**
STUDY SUMMARY IN LOCAL LANGUAGE **15**
ABBREVIATIONS **15**
STUDY SCHEDULE OF ASSESSMENTS **18**
1. STUDY ADMINISTRATIVE STRUCTURE **20**
1.1 Sponsor, Sponsor-Investigator 20
1.2 Principal Investigator(s) 20
1.3 Statistician 20
1.4 Local Site Investigators 20
1.5 Laboratory 20
1.6 Monitoring institution 21
1.7 Data Safety Monitoring Committee 21
1.8 Any other relevant Committee, Person, Organisation, Institution 21
2. ETHICAL AND REGULATORY ASPECTS **21**
2.1 Study registration 21
2.2 Categorisation of study 21
2.3 Competent Ethics Committee (CEC) 21
2.4 Competent Authorities (CA) 21
2.5 Ethical Conduct of the Study 22
2.6 Declaration of interest 22
2.7 Patient Information and Informed Consent 22
2.8 Participant privacy and confidentiality 22
2.9 Early termination of the study 22
2.10 Protocol amendments 23
3. BACKGROUND AND RATIONALE **23**
3.1 Background and Rationale 23
3.2 Investigational Product and Indication 25
3.3 Preclinical Evidence 26
3.4 Clinical Evidence to Date 26
3.5 Dose Rationale / Medical Device: Rationale for the intended purpose in study (pre-market MD) 27
3.6 Explanation for choice of comparator (or placebo) 27
3.7 Risks / Benefits 27
3.8 Justification of choice of study population 28
4. STUDY OBJECTIVES **28**
4.1 Overall Objectives 28
4.2 Primary Objective 28
4.3 Secondary Objectives 28
4.4 Safety Objectives 29
5. STUDY OUTCOMES **29**
5.1 Primary Outcome 29
5.2 Secondary Outcomes 29
5.3 Safety Outcomes 29
5.4 Other Outcomes 29
6. STUDY DESIGN **30**
6.1 General study design and justification of design 30
6.2 Methods of minimising bias 30
    6.2.1 Randomisation 30
    6.2.2 Blinding procedures 31

6.3 Unblinding Procedures (Code break) ... 31
7. STUDY POPULATION ... 31
7.1 Eligibility criteria ... 31
7.2 Recruitment and screening ... 32
7.3 Assignment to study groups ... 32
7.4 Criteria for withdrawal / discontinuation of participants ... 32
8. STUDY INTERVENTION ... 32
8.1 Identity of Investigational Products (treatment / medical device) ... 32
8.1.1 Experimental Intervention (treatment / medical device) ... 33
8.1.2 Control Intervention (standard/routine/comparator treatment / medical device) ... 33
8.1.3 Packaging, Labelling and Supply (re-supply) ... 33
8.1.4 Storage Conditions ... 33
8.2 Administration of experimental and control interventions ... 34
8.2.1 Experimental Intervention ... 34
8.2.2 Control Intervention ... 35
8.3 Dose / Device modifications ... 35
8.4 Compliance with study intervention ... 35
8.5 Data Collection and Follow-up for withdrawn participants ... 35
8.6 Trial specific preventive measures ... 35
8.7 Concomitant Interventions (treatments) ... 36
8.8 Study Drug accountability ... 36
8.9 Return or Destruction of Study Drug / Medical Device ... 37
9. STUDY ASSESSMENTS ... 37
9.1. Schedule of assessments ... 37
9.2 Assessment of outcome ... 37
9.2.1 Assessment of primary outcome ... 37
9.2.2 Assessment of secondary outcomes ... 38
9.2.3 Assessment of safety outcomes ... 39
9.2.4 Assessments in participants who prematurely stop the study ... 39
9.3 Procedures at each visit ... 39
9.3.1 Screening ... 40
9.3.2 Intervention phase: ... 41
9.3.3 Short-term Follow-up Visit (End-of-treatment visit) ... 42
9.3.4 Follow-up Visits (+/- 14) (each 3 months for 5 years) ... 43
10. SAFETY ... 43
10.1 Drug studies ... 43
10.1.1 Definition and assessment of (serious) adverse events and other safety related events43
10.1.2 Reporting of serious adverse events (SAE) and other safety related events ... 45
10.1.3 Follow up of (Serious) Adverse Events ... 46
11. STATISTICAL METHODS ... 46
11.1 Hypothesis ... 47
11.2 Determination of Sample Size ... 47
11.3 Statistical criteria of termination of trial ... 47
11.4 Planned Analyses ... 47
11.4.1 Datasets to be analysed, analysis populations ... 47
11.4.2 Primary Analysis ... 47
11.4.3 Secondary Analyses ... 47
11.4.4 Interim analyses ... 47
11.4.5 Safety analysis ... 47
11.4.6 Deviation(s) from the original statistical plan ... 47

11.5 Handling of missing data and drop-outs 48
12. QUALITY ASSURANCE AND CONTROL 48
12.1 Data handling and record keeping / archiving 48
12.1.1 Case Report Forms 48
12.1.2 Specification of source documents 48
12.1.3 Record keeping / archiving 49
12.2 Data management 49
12.2.1 Data Management System 49
12.2.2 Data security, access and back-up 49
12.2.3 Analysis and archiving 50
12.2.4 Electronic and central data validation 50
12.3 Monitoring 50
12.4 Audits and Inspections 50
12.5 Confidentiality, Data Protection 50
12.6 Storage of biological material and related health data 51
13. PUBLICATION AND DISSEMINATION POLICY 51
14. FUNDING AND SUPPORT 52
14.1 Funding 52
14.2 Other Support 52
15. INSURANCE 52
16. REFERENCES 53
17. APPENDICES 58

Study synopsis

**Principal-Site Investigators**<br><br>**Sponsor-Investigator**	Dr. Intidhar Labidi-Galy (HUG)<br>Dr. Antonella Diciolla (CHUV)<br><br>Prof. Nicolas Mach, Hôpitaux Universitaires de Genève (HUG)
**Study Title:**	A PHASE IB TRIAL OF INTRAPERITONEAL CISPLATIN AND NAB-PACLITAXEL ADMINISTERED BY PRESSURIZED INTRAPERITONEAL AEROSOL CHEMOTHERAPY (PIPAC) IN THE TREATMENT OF ADVANCED MALIGNANCIES CONFINED TO THE PERITONEAL CAVITY
**Short Title / Study ID:**	PIPAC with Nab-paclitaxel and cisplatin in peritoneal carcinomatosis, a Phase IB Study/study ID
**Protocol Version and Date:**	V5.0 17.01.2023
**Trial registration:**	www.clinicaltrials.gov number NCT04000906
**Study category and Rationale**	Categorisation of clinical trials of medicinal products: Category B.<br>PIPAC is a laparoscopic procedure for intraperitoneal administration of chemotherapy; cisplatin (Cisplatin-Teva) has been administered by PIPAC with a good tolerance. Intraperitoneal route of Nab-paclitaxel (Abraxane®) has been studied in a phase I clinical trial with a good tolerance profile. Cisplatin and paclitaxel or Nab-paclitaxel administered intravenously are approved for the treatment of cancers with peritoneal carcinomatosis such as ovarian, gastric and pancreatic cancers or primitive peritoneal mesothelioma.
**Clinical Phase:**	Interventional Phase IB clinical trial, open label, single group assignment
**Hypothesis**	Preclinical reports have shown that Nab-paclitaxel (Abraxane®), a nanoparticule albumin-bound formulation of paclitaxel, has an enhanced antitumoral activity due to its internalization through macropinocytosis by the macrophages of the tumor environment (TAMs) leading to anti-tumoral immunomodulatory effect. We hypothesized nab-paclitaxel could be a good candidate for intraperitoneal administration by PIPAC in patients with peritoneal carcinomatosis, as this route could reduce systemic toxicity and increase intratumoral drug concentration. We are expecting that this local intervention might rebalance favorably the immune environment and will lead to a prolonged local control and potentially a survival benefit. As PIPAC procedure is usually repeated three times, it would be important to observe changes of tumor mutational profile and the immunological responses of the microenvironment in vivo.<br>We are hoping to demonstrate that the administration of new chemotherapy components, in particular nanomedicine, is safe and will pave the way for future development in association with novel immunotherapies.

**Background and Rationale:**	Intraperitoneal dissemination of malignant tumors is a major problem in the management of digestive and gynecological cancers resulting in very poor prognosis and a rapid marked deterioration in the quality of life of these patients. Malignancies that most likely spread to the peritoneum include gastric (60%), pancreatic (50%), ovarian (70%), colorectal (35%), peritoneal mesothelioma and pseudomyxoma peritonei.<br>Maintaining the quality of life of patients in palliative oncology is of high importance. Surgical and / or systemic treatments (intravenous chemotherapy) have limited efficacy in the palliation of symptoms related to peritoneal carcinomatosis at the cost of systemic toxicities, which are usually significant. Intravenous chemotherapy efficacy is usually short lived due to poor penetration into the peritoneal cavity. The role of intraperitoneal chemotherapy (IP) is to maximize tumor penetration and optimize cell death while minimizing systemic toxicity. Intraperitoneal chemotherapy retains several advantages: i) the dose intensity delivered to peritoneal carcinomatosis increases as half-life is longer, ii) intraperitoneal concentration is higher and iii) systemic toxicity reabsorption is weaker than intravenous route (1). Intraperitoneal chemotherapy (IP) is a recommended treatment for epithelial ovarian cancer in combination with a fully reductive surgery and is the standard treatment of pseudomyxoma peritonei (2-10) (11-13). It has also been studied in several cancers of digestive origin (14).<br>Pressurized intraperitoneal aerosol chemotherapy (PIPAC) is a new laparoscopic procedure of about 90 minutes duration used for the intraperitoneal application of a pressurized aerolization of chemotherapy which increases the therapeutic ratio of the substance administered between the local and systemic concentrations by 2-3-fold, resulting in an improvement of the local efficacy and reduction of the systemic toxicity. The application of repeated four weeks' interval cycles increases the efficacy compared to systemic treatments. Taking advantage of physical laws, PIPAC generates an artificial pressure gradient enhancing tissue drug uptake by convection and distributing drugs more homogeneously within the closed and expanded abdominal cavity (15-23). The intraperitoneal administration of chemotherapy is reimbursed by a specific DRG. With a standardized surgical approach and dedicated safety checklist, PIPAC could be safely introduced in clinical routine with minimal learning curve (24).<br>This novel procedure was shown to be feasible in most patients with refractory carcinomatosis of various origins. Several retrospective and five prospective trials have shown that PIPAC can be used safely. Intraoperative complications and postoperative morbidity rates were low. Surgery-related complications occurred in up to 12 per cent and postoperative morbidity was low (CTCAE grade 3-5 events reported in 0-37%). Regarding technical feasibility, laparoscopic access and repeatability were 83-100 and 38-82% (25). PIPAC was followed by a modest and transitory inflammatory response that was commensurate to the disease extent. No hematological, renal or hepatic toxicity was observed even after repetitive administration (26). Quality of life and symptoms were not impacted by PIPAC treatment (27). The available evidence on PIPAC was scrutinized in a systematic review confirming that PIPAC treatment was feasible, safe and well tolerated (28). Preliminary good response rates call for prospective analysis of oncological efficacy. The overall tumor response ranged between 40 to 75% in peritoneal carcinomatosis of ovarian and gastric origin with 3 successive PIPAC treatments with cisplatin and doxorubicin.

	Current practice of the new drug administration method was studied within an international expert panel showing excellent standardization of PIPAC among expert centers opening the door for registries and multi-center studies(29). There are currently several ongoing trials reported on clinicaltrials.gov in addition to those already published such as *NCT03294252, NCT03172416, NCT03210298, NCT03280511, NCT02735928, NCT03100708, NCT03287375, NCT02604784, NCT03304210*. Nowadays doxorubicine, cisplatin, oxaliplatin, mitomycine C and irinotecan have been used with PIPAC (off-label use). Classical chemotherapy components used intravenously for ovarian, gastric and pancreatic neoplasias belong to the taxane and platin cytostatic families. A phase I conducted by the german group, aiming to report the MTD of cisplatin and doxorubicin administered intraperitoneally by PIPAC has found that dose level of 10.5 mg/m^2 and 2.1 mg/m^2 of cisplatin and doxorubicin respectively were feasible and well tolerated. MTD was not reached in this study. This study confirms the safety of PIPAC and the low systemic reabsorption of chemotherapy administered by this route. They did not report any significant hematologic toxicity, neither hepatic, neurological nor renal toxicity (30).<br><br>Nab-paclitaxel is a next-generation taxane that has been specifically designed to overcome the limitations of conventional paclitaxel formulations, including the barriers to effective drug delivery of highly lipophilic agents (31). It is an albumin-drug complex of about 130 nm in diameter and is distributed similarly to endogenous albumin. Nanoparticles within this size range accumulate in solid tumors due to the enhanced permeability and retention effect (EPR), which results from an aberrant and leaky tumor vasculature and the lack of poorly developed lymphatic drainage system in many solid tumors. By exploiting the natural interactions between albumin and the gp60/caveolin-1 receptor pathway, it has been associated with rapid and preferential delivery and accumulation of paclitaxel at the tumor site. Pharmacokinetic studies have shown that, compared with paclitaxel, nab-paclitaxel is associated with a 9-fold greater penetration of paclitaxel into tissues via transporter-mediated pathways, a 33% higher intratumoral drug concentration, a 10-fold higher mean maximal concentration of free paclitaxel, and a 4-fold lower elimination rate (32-34).<br>It is currently approved intravenously for breast, non–small cell lung (NSCLC), and pancreatic cancers. It has been studied intravenously in three phases II clinical trials for recurrent ovarian cancer and in two phases II and a phase III trial for recurrent gastric adenocarcinoma. A recent phase I trial examined the maximally tolerated dose (MTD) of IP administered nab-paclitaxel in peritoneal carcinomatosis mostly from gynecologic and gastro-intestinal origin. The reached MTD was 140 mg/m^2. IP administration of nab-paclitaxel showed higher peritoneal exposure to the drug compared with the plasma exposure with a low inter- and intra-patient variability (35).

	There is scarce published data on the optimal dilution of nab-paclitaxel. However in the light of published literature, it appears that its stability and efficacy poperties are not impaired with a 10 times dilution (36-38).<br><br>Preclinical and recent clinical studies have shown that nab-paclitaxel has better penetration into the tumor cells due to its macropinocytosis internalization by macrophages of tumor environment (TAMs). Macropinocytosis is a form of endocytosis in which a large fluid-filled vesicle is pinched off from the cell membrane and brought into the interior of the cell. This is particulary relevant as peritoneal carcinomatosis from tumors highly infiltrated by TAMs have an especially poor prognosis and this hold true for pancreatic, gastric and ovarian carcinoma. Tumor-associated macrophages (TAMs) may be polarized in two phenotypes: type M1 or type M2. TAMs of the M2 phenotype are known to promote tumor proliferation by suppressing anti-tumor immune reactions and inducing angiogenesis and are associated with a poor prognosis in numerous cancers (39). On the contrary, a ratio favoring type M1 TAMs confers a better prognosis (40). A new mechanism of action of nab-paclitaxel recently identified in preclinical models of pancreatic cancers is its capacity to polarize TAMs toward M1 activation state (41).<br><br>In addition to a direct cytotoxic effect on cancer cells, nab-paclitaxel has immunostimulatory properties and might therefore improve the immune surveillance and prognosis of peritoneal carcinomatosis. It is becoming more and more obvious that novel cancer immunotherapies may require « priming » for an enhanced and prolonged response. Pancreatic, breast cancers and NSCLC, for which nab-paclitaxel is an approved treatment regimen, are highly infiltrated by TAMs (41). Current reports of nab-paclitaxel with check-point inhibitors are limited, but early data suggest that nab-paclitaxel-based therapy may augment the antitumor activity of immune-checkpoint inhibitors and may double or triple the overall response rate or act as a sensitizer for immune checkpoint blockade therapy (42) (43) (44). Early clinical trials have indicated that pancreatic tumors may be fully resistant to monotherapy with immune checkpoint inhibitors (45, 46). However, recent data from a mouse-model study demonstrated that this resistance could be overcome with a combination therapy that contained nab-paclitaxel (47, 48). Nab-Paclitaxel is currently being studied in combination with nivolumab or pembrolizumab in pancreatic cancer, as well as with other checkpoint inhibitors (atezolizumab, durvalumab and ipilimumab) in multiple trials of solid tumors.
**Objective(s):**	The **primary objective** of this phase IB study is to assess the safety and tolerability of IP administration by PIPAC of Nab-paclitaxel (Abraxane®) and cisplatin in patients with peritoneal carcinomatosis.<br>The **secondary objectives** are to evaluate i) the response rate in patients with peritoneal carcinomatosis; ii) the impact on quality of life (QoL); iii) the reproducibility of radiological PCI evaluation by CT enterography (CT-PCI score), when available, and its correlation with surgical PCI evaluation; and iv) the changes of intratumoral immune response.

**Outcome(s):**	The **main outcome** of this phase IB study is to determine the maximal tolerated dose (MTD) of Nab-paclitaxel (Abraxane®) administered IP by PIPAC in concomitance with cisplatin. MTD is defined as the lowest dose level at which ≥33% of patients' experience dose limiting toxicity in accordance to CTCAE version 5.0 criteria.<br>The **secondary outcomes** are:<br>- Adverse events (AE) and serious adverse events (SAE) with predefined toxicity criteria will be applied using CTCAE version 5.0 criteria, documented before and after each cycle of PIPAC treatment. Surgical complications will be assessed according to Clavien classification and comprehensive complication index (CCI) (Time frame D-0/D10 of each cycle).<br>- The efficacy as assessed by the objective histological regression and objective tumor response rate (OTR) according to the new regression system for peritoneal cancer (PRGS, peritoneal regression grade score system). Time frame (D1 of each cycle). The objective response rate (ORR), the clinical benefit rate (CBR) as defined by RECIST version 1.1 criteria and the relevance of radiological response assessed by CT enterography (CT-PCI score) when available. Time frame (Screening and EOT visit).<br>- The QoL will be evaluated based on the EORTC questionnaire QLQ-C30 Version 3.0 and visual analogic scale for pain (VAS scale). Time frame (D0/D10 of each cycle).<br>**Other outcomes of interest:**<br>- The intratumoral immune response changes following PIPAC as assessed by multiplexed immunohistochemistry (IHC). Time frame (D1 of each PIPAC cycle). The molecular profile of the tumor will be assessed by Next Generation Sequencing of DNA (whole-exome sequencing) and RNA (RNAseq) before and after treatment. Time frame (D1 of each PIPAC cycle). In a latter phase, pharmacokinetic analysis of plasmatic concentrations of nab-paclitaxel (Abraxane®) will be performed at pre-dose, end of infusion, H1, H4 and 24 hours after the first PIPAC treatment for the two first patients treated for each dose level escalation.
**Study design:**	The study design for nab-paclitaxel dose escalation is based on a modified Fibonacci sequence (dose increase by 100%, 67%, 50%, 40%, and 33% for all the rest): 7.5 mg/m^2, 15 mg/m^2, 25 mg/m^2, 37.5 mg/m^2, 52.5 mg/m^2 and 70 mg/m^2. Three patients are treated at each dose level (DL) until the first DLT (defined as grade 3 or 4, CTCAE version 5.0 with the exceptions clarified in chapter 9.2.1) occurred in the first cycle of treatment.<br><br>If one patient among the three of the first cohort experiences a DLT within 4 weeks from the first cycle of PIPAC treatment, then an additional cohort of 3 patients is treated at the same DL. If no patient among 3 or 1 among 6 patients experiences a DLT within 4 weeks from the first cycle of PIPAC treatment, the dose is escalated. MTD (maximal tolerated dose) defined as the lowest dose level at which ≥33% (≥ 2/6) subjects experienced DLT during the first cycle of treatment.

**Inclusion / Exclusion criteria:**	**Inclusion**:<br>- Informed consent as documented by signature<br>- ≥18 years,<br>- psychologically able to follow the trial procedures<br>- with peritoneal carcinomatosis from pancreatic, oeso-gastric, epithelial ovarian cancers or primitive peritoneal mesothelioma,<br>- ECOG 0, 1 or 2,<br>- Life expectancy > 3 months,<br>- Not candidate for surgical cytoreduction and IP/HIPEC based on expert multidisciplinary board<br>- Who received at least one line of chemotherapy and for whom standard therapies have been exhausted or not feasible. Patients with residual disease following the first line of therapy or following secondary debulking are eligible.<br>**Exclusion**:<br>- Predominant extra-peritoneal metastases at the discretion of the study team after discussion at the multidisciplinary tumor board,<br>- Bowel obstruction, clinically significant active gastro-duodenal ulcer or ongoing abdominal infection (bacterial, viral or fungal),<br>- Chemotherapy or surgery within the last two weeks prior to C1D1,<br>- General or local (abdominal) contra-indications for laparoscopic surgery<br>- Known allergy to cisplatin or other platinum-containing compounds or to nab-paclitaxel,<br>- Severe renal impairment (calculated GFR (CKD-EPI) < 50 mL/min/1.73 m2), myelosuppression (platelet count < 100.000/µl, hemoglobin < 9g/dl, neutrophil granulocytes < 1.500/ml), severe hepatic (Serum total bilirubin ≥ 1.5 mg/dl, AST or ALT >5 x ULN), respiratory or neurologic impairment (grade 3), severe myocardial insufficiency (NYHA class > 2), recent myocardial infarction, severe arrhythmias,<br>- Prophylactic or therapeutic anticoagulant cannot be safely interrupted for the PIPAC procedures,<br>- Pregnancy or breastfeeding, women who can become pregnant must ensure effective contraception.<br>- Known or suspected non-compliance, inability to follow the procedures of the study, e.g. due to language problems, psychological disorders, dementia, etc. of the participant,<br>- Has a history or current evidence of any condition, therapy, or laboratory abnormality that might confound the results of the trial, interfere with the subject's participation for the full duration of the trial, or is not in the best interest of the subject to participate, in the opinion of the treating investigator.

**Measurements and procedures:**	**Choice of the dosage of the cyclostatic products**<br>The choice of 10.5 mg/m^2 cisplatin dose has been based on the result of a recent phase I escalation dose of cisplatin and doxorubicin association administered IP by PIPAC (30). The MTD in a phase I of IP nab-paclitaxel administration by catheterism was 140 mg/m^2 (35). Thus, we are expecting a lower MTD for this phase I as PIPAC has higher activity and penetration compared to conventional IP treatment.<br><br>PIPAC procedure:<br>- Intervention under general anesthesia<br>- Antibiotic prophylaxis with commercial cephalosporine administered during anesthetic induction<br>- Introduction of a 5mm optic after insufflation with Hasson technique<br>- Insufflation capnoperitoneum at 12 mmHg and insertion of 1 trocar of 10/12 for the nebulizer, 1 trocar for the camera and 1 working trocar (ascites removal, biopsies, peritonectomy).<br>- Removal of ascites and documentation of the volume and cytology<br>- Documentation of the peritoneal carcinomatosis index (surgical PCI score and Fagotti score for ovarian carcinoma)<br>- 5 punch peritoneal biopsies in the 4 quadrants of the abdomen and 1 peritonectomy of several cm^2 (See Appendice C & G)<br>- Connection of the nebulizer (Capnopen®, Capnomed GmbH, Villigendorf, Germany) to a high-pressure injector (Accutron HP-D Injecteur, Medtron AG) and insertion into the abdomen.<br>- Pressurized dose of cisplatin and nab-paclitaxel (Abraxane®) at escalating doses will be applied via the nebulizer and injector, with a flow rate of 0.6-0.7ml/s and a median droplet size of 11 (3-15) µm<br>- Maximal upstream pressure of 300 psi.<br>- Treatment to be maintained for 30 minutes after administration at ambient temperature and a pressure of 12 mmHg.<br>**Protection of personnel:**<br>- Documentation of tightness of the abdomen via maximum CO2 flow of 0-0.4 L/min corresponding to the body resorption of CO2.<br>- Operation to be performed in an operating room equipped with ventilation system<br>- Remote control of injection of chemotherapy<br>- Elimination of chemotherapy by exsufflation via a closed line over two sequential microparticle filters into the airwaste system of the hospital<br>- Retraction of the trocars and elimination in special adapted cytostatic bins<br>- Video recording of every procedure<br>Check list to double-check safety measures before administration of cytostatics

**Study Product / Intervention:**	Escalating doses of nab-paclitaxel (Abraxane®) in 150 ml NaCl 0,9% and cisplatin 10.5 mg/m^2 body surface in 150 ml NaCl 0,9% (concentration of 1mg/ml) will be applied sequentially, starting with nab-paclitaxel, through a nebulizer (Capnopen®, Capnomed GmbH, Villigendorf, Germany) to a high-pressure injector (Accutron HP-D Injecteur, Medtron AG) with a flow rate of 0.6-0.7 ml/s and a median droplet size of 11 (3-15) µm at ambient temperature and at a maximal upstream pressure of 300 psi intraperitoneally. Treatment is to be maintained for 30 minutes after administration at a pressure of 12 mmHg.<br><br>PIPAC administration will be repeated q4-6 weeks intervals for a total of 3 courses
**Control Intervention (if applicable):**	Not applicable.
**Number of Participants with Rationale:**	An estimated number of 6 to 36 patients is expected to determine the MTD for this phase IB study with a modified Fibonacci escalation 3+3 design.
**Study Duration:**	The estimated duration of this phase IB study from the start of screening of the first participant to the last participant processed and treated is 18 months.
**Study Schedule:**	Month Year of First-Participant-In (planned): June 2020<br>Month Year of Last-Participant-Out (planned): June 2022
**Other relevant Investigator(s):**	Dr Frédéric Ris, Department of Surgery, HUG<br><br>Dr Manuela Undurraga Malinverno, Department of Gynecology, HUG<br><br>Dr Martin Hübner, Department of Surgery, CHUV<br>Dr Noémie Lang, Department of Oncology, HUG
**Study Centre(s):**	Hôpitaux Universitaires de Geneve (HUG)<br>Centre Hospitalier Universitaire Vaudois (CHUV)
**Statistical Considerations:**	We expect an estimated number of 6 to 36 patients to determine the MTD for this phase IB study with a modified Fibonacci escalation 3+3 design.
**GCP Statement:**	This phase IB study will be conducted in compliance with the protocol, the current version of the Declaration of Helsinki, the ICH-GCP or ISO EN 14155 (as far as applicable) as well as all national legal and regulatory requirements.

Study summary in local language

La diffusion intrapéritonéale des tumeurs malignes est un problème majeur dans la prise en charge des cancers. Elle entraine une deterioration importante de la qualité de vie des patients et un pronostic sombre. L'administration intra-péritonéale de la chimiothérapie permet de traiter directement la carcinose péritonéale, et a montré une certaine efficacité dans les cancers de l'ovaire et du colon, mais au prix d'une morbidité importante. L'administration intraperitonéale pressurisée de chimiothérapie (PIPAC) est une nouvelle technique chirurgicale peu invasive qui permet d'administrer les medicaments dans la cavité abdominale sous forme d'aérosols lors d'une laparoscopie. Ce dispositif médical permet une augmentation de l'index therapeutique entre les concentrations locales et systémiques des drogues de chimiothérapie, l'objectif étant d'augmenter l'efficacité locale tout en réduisant la toxicité systémique.
L'étude NAB-PIPAC est un essai clinique de phase IB qui a pour but d'évaluer la tolérance et la faisabilité d'un traitement associant l'administration de cisplatine et nab-paclitaxel par PIPAC chez les patients en situation palliative traités pour une carcinose peritonéale d'origine ovarienne, oeso-gastrique, peritoneale ou pancréatique. Elle comprend une analyse translationnelle sur les prélévements histologiques réalisées avant et après l'intervention afin d'analyser les changements opérés au niveau du microenvironnement tumoral et des altérations moléculaires au sein de la tumeur.

Abbreviations

ADLs= activities of daily living
AE= Adverse event
ASR= Annual Safety Report
AUC= Area under the curve
CA= Competent Authority
CBR= Clinical Benefit Rate
CCER= Commission cantonale d'ethique de la recherche de Geneve
CCI= Comprehensive Complication Index
CE=European community
CEC=Competent Ethics Committee
CER-VD= Commission cantonale d'ethique de la recherché sur l'etre humain-Canton de Vaud

CHUV= Centre Hospitalier Universitaire Vaudois
Cmax= Maximal concentration
CO2= Carbone Dioxide
CRA= Clinical research associate
CRC= Clinical Research Center in HUG
CT= Computed tomography
CTCAE= Common terminology criteria for adverse events
CTLA= Clinical trial letter of agreement
CT-PCI= Radiological Peritoneal Carcinomatosis Index assessed by CT-scan
CYP= Cytochrome P
DFDL= Dr Henri Dubois-Ferrière Dinu Lipatti Foundation Onco-Haematology Research Unit
DL= Dose Level
DLT= Dose Limiting toxicity
ECG= Electrocardiogram
ECOG= Eastern Cooperative Oncology Group
eCRF= Electronic Case Report Form
EORTC= European Organisation for Research and Treatment of Cancer
EOT= End-of-Treatment
FCBG= Fondation Campus Biotech Geneva
FFPE= Formalin-Fixed Paraffin Embedded
GCP= Good Clinical Practice
HIPEC= Hyperthermic Intraperitoneal Chemotherapy
HPR=Histopathologic regression
HUG= Hopitaux Universitaires de Geneve
IMP= Investigational Medicinal Product
ICH-GCP=International Conference on Harmonisation-Good Clinical Practice
IEC= Institutional Ethics Committee
IHC= Immunohistochemistry
IP= Intraperitoneal
IRB= Institutional Review Board
ISO = International Organization for Standardization
ITT= Intention to treat
IV= Intravenous
LOCF= Last observation carried forward
MD= Medical Device
Min=Minute
Ml=Milliliter
MTD=Maximum tolerated dose
NaCl= Sodium Chlorure
NCT=National clinical trial
NSCLC= Non-small cell lung cancer
NYHA= New York Heart Association
ORR= Objective response rate
OTR=Objective tumor response
PC= Peritoneal carcinomatosis
PCI= Peritoneal Carcinomatosis Index
PD= Progressive Disease
PD-L1= Programmed death-ligand 1
PI= Principal Investigator
PIPAC= Pressurized intraperitoneal aerosol chemotherapy
PK= Pharmacokinetics

PRGS= Peritoneal Regression Grade Score system
PSI= pound-force per square inch
QLQ= Quality of Life Questionnaire
QoL= Quality of Life
RECIST= Response Evaluation Criteria In Solid Tumors
SAE=Serious adverse event
SAKK= Swiss Group for Clinical Cancer Research
SDV= Source Data Verification
SOP= Standard Operating Procedure
SPC= Summary of product characteristics
SUSAR= Suspected Unexpected Serious Adverse Reaction
TAM= Tumor-associated macrophages
TMF= Trial Master File
UNL= Upper normal limit
UPN = Unique Patient Number
VAS=Visual analgesic scale for pain

Study schedule of assessments

Visit Number	1	2	3	4	5	6	7	8	9	10	11	12	13	14	15	16	17	18
**Visit Name**	**Screening**	**C1▪ D0**	**C1 D1**	**C1 D2**	**C1 D3**	**C1 D10**	**C2 D0**	**C2 D1**	**C2 D2**	**C2 D3**	**C2 D10**	**C3* D0**	**C3* D1**	**C3* D2**	**C3* D3**	**C3* D10**	**CXD56[a]**	**Q3M**
**Scheduling Window**	-28	- 3				+/- 3	- 3	+/-3			+/-3	- 3	+/- 3			+/- 3	+/- 7	+/- 14
Patient Information & Consent	x																	
Demographics	x																	
Medical History	x																	
In- /Ex- Criteria	x																	
Physical Examination[b]	x	x	x	x	x	x	x	x	x	x	x	x	x	x	x	x	x	
ECOG score	x	x				x	x				x	x				x	x	
AE/SAE	x	x	x	x	x	x	x	x	x	x	x	x	x	x	x	x	x	
DLT evaluation[c]							x											
Concomitant Medication	x	x	x	x	x	x	x	x	x	x	x	x	x	x	x	x	x	
Vital Signs[d]	x	x	x	x	x	x	x	x	x	x	x	x	x	x	x	x	x	
Hematology[e]	x[e]	x				x	x				x	x				x	x	
Serum Chemistry[e]	x[e]	x				x	x				x	x				x	x	
Coagulation[e]	x[e]																	
Serum Tumor biomarker[e]	x[e]						x					x					x	
Urine analysis[e]	x[e]																	
Pregnancy Test	(x)[f]	(x)					(x)					(x)						
Chest CT	x																x	
CT enterography[g] (CT-PCI score)	x											X[h]					x	
ECG	x																	
Ascite volume			x					x					x					
PIPAC			x					x					x					

**Visit Number**	1	2	3	4	5	6	7	8	9	10	11	12	13	14	15	16	17	18
**Visit Name**	**Screening**	**C1▪ D0**	**C1 D1**	**C1 D2**	**C1 D3**	**C1 D10**	**C2 D0**	**C2 D1**	**C2 D2**	**C2 D3**	**C2 D10**	**C3* D0**	**C3* D1**	**C3* D2**	**C3* D3**	**C3* D10**	**CXD56[a]**	**Q3M**
**Scheduling Window**	-28	- 3				+/- 3	- 3	+/-3			+/-3	- 3	+/- 3			+/- 3	+/- 7	+/- 14
Surgical PCI score			x					x					x					
Biopsies (standard & translational)			x					x					x					
Blood sampling (translational research)		x																
PK (free plasmatic nab-paclitaxel)[i]			x	x														
QoL		x				x	x				x	x				x	x	
VAS[j]	x	x	x	x	x	x	x	x	x	x	x	x	x	x	x	x	x	
Survival status																		x
Pathology and Molecular analysis			x															
Disease status																		x

▪ Labs performed within 7 days of treeatments initiation during screening do not need to be repeated at D0 unless clinically indicated

[a] To be performed within 28 days after the last PIPAC administration and/or prior the start of the next ancicancer treatment

[b] Neurological examination with pallesthesia and abdominal circumference only requested at screening visit

[c] For Dose Limiting Toxicities, refer to the section 9.2.1

[d] See Appendix H (Abdominal circumference only Screening, C2D0, C3D-0 and EoT visit)

[e] To be performed within 10 days prior to registration

[f] To be performed within 7 days prior to registration

[g] when available

[h] 3 days before in order to assess the PCI score before the next administration

[i] Blood samples for pharmacokinetic analysis will be performed at the following timepoints: Pre-dose 30 minutes [± 5 minutes], end of infusion [± 15 minutes], 1 hour [± 15 minutes], 4 hours [± 30 minutes], 24 hours [± 4 hours]. Samples collected at the first cycle of PIPAC for the two first patients treated for each dose level escalation. More details in Appendix F

[j] highest value of the day taken in bed

* for patients in the study for whom the treatment is effective, the visit of this cycle will be repeated as long as it is necessary and reported in the e-CRF.

1. STUDY ADMINISTRATIVE STRUCTURE

1.1 Sponsor, Sponsor-Investigator

The "Hôpitaux Universitaires de Genève" (HUG) will be the sponsor and will provide the capnomed device and injector to perform PIPAC procedure in Geneva and the cisplatin to perform the study in Geneva and Lausanne. The "Centre Hospitalier Universitaire Vaudois" (CHUV) will provide the capnomed device and injector to perform PIPAC procedure at Lausanne. The collection and interpretation of all data and the writing of the report will be done independently from the Sponsor.

Sponsor
Prof. Nicolas Mach, MD,
Head of the DFDL Onco-Haematology Research Unit
Departement of Oncology
Geneva University Hospitals and medical school (HUG)
Nicolas.Mach@hcuge.ch (+41)22 37 22 901

Hôpitaux Universitaires Genevois (HUG)
Rue Gabrielle-Perret-Gentil 4
1205 Genève

1.2 Principal Investigator(s)

Principal Investigator/ Investigator Coordinator
Dr. Intidhar Labidi-Galy,
Attending physician
Departement of Oncology
Geneva University Hospitals and medical school (HUG)
Intidhar.labidi-galy@hcuge.ch (+41)79 55 34 976

Principal Investigator
Dr. Antonella Diciolla
Consultant physician
Departement of Oncology
Lausanne University Hospital and medical school (CHUV)
Antonella.Diciolla@chuv.ch (+41)79 55 63 011

1.3 Statistician

See Study contact list (Appendix I)

1.4 Local Site Investigators

See Study contact list (Appendix I)

1.5 Laboratory

See Study contact list (Appendix I)

1.6 Monitoring institution
See Study contact list (Appendix I)

1.7 Data Safety Monitoring Committee
Not applicable.

1.8 Any other relevant Committee, Person, Organisation, Institution
Not applicable

2. ETHICAL AND REGULATORY ASPECTS

The approval of the Commission cantonale d'ethique de la recherche de Genève (CCER), la Commission cantonale (VD) d'éthique de la recherche sur l'être humain (CER-VD) and Swissmedic concerning the conduct of the study, the protocol, the proposed patient information and consent form as well as other study-specific documents will be obtained in writing by the Sponsor-Investigator before commencement of this study. The clinical study can only begin once approval for all the study-specific documents from all required authorities has been received. Any additional requirements imposed by the authorities will be implemented. Any amendment to the protocol will be as well approved (if legally required) by these institutions.

2.1 Study registration
The study is registered at www.clinicaltrials.gov (NCT04000906)
Swiss National Clinical trial Portal (SNCTP000003129 via BASEC).

2.2 Categorisation of study
The clinical trial comes under category B (clinical trials of medicinal products).

Cisplatin-Teva and Abraxane (nab-paclitaxel) are authorized in Switzerland and will not be used in an indication within the same disease group of the International Classification of Diseases. However, the dosage of the medicinal product is lower than that specified in the prescribing information.
All devices used for the PIPAC procedure have a CE marking.

2.3 Competent Ethics Committee (CEC)
The responsible investigator at each site ensures that approval from an appropriately constituted Competent Ethics Committee (CEC) is sought for the clinical study. The reporting duties and allowed time frame (all changes in the research activity and all unanticipated problems involving risks to humans; including in case of planned or premature study end and the final report) and no changes are made to the protocol without prior Sponsor and CEC approval, except where necessary to eliminate apparent immediate hazards to study participants. Premature study end or interruption of the study will be reported within 15 days. The regular end of the study will be reported to the CEC within 90 days, the final study report will be submitted within one year after study end. Amendments will be reported according to chapter 2.10.

2.4 Competent Authorities (CA)
The Sponsor will obtain approval from Swissmedic before the start of the clinical trial. The reporting duties and allowed time frame to Swissmedic including the reporting duties in case of planned or premature study end and the final report will be followed. Reporting duties and

timelines will be the same as for CEC, except of non-substantial amendments that will be reported as soon as possible. Amendments will be reported according to chapter 2.10.

2.5 Ethical Conduct of the Study

The study will be carried out in accordance to the protocol and with principles enunciated in the current version of the Declaration of Helsinki, the guidelines of Good Clinical Practice (GCP) issued by ICH and the Swiss Law and Swiss regulatory authority's requirements. The CEC and regulatory authorities will receive annual safety and interim reports and be informed about study stop/end in agreement with local requirements.

2.6 Declaration of interest

The authors of this study protocol certify that they have NO affiliations with or involvement in any organization or entity with any financial interest (such as honoraria; educational grants; participation in speakers' bureaus; membership, employment, consultancies, stock ownership, or other equity interest; and expert testimony or patent-licensing arrangements), or non-financial interest (such as personal or professional relationships, affiliations, knowledge or beliefs) in the subject matter or materials discussed in this protocol.

2.7 Patient Information and Informed Consent

The investigator's team will explain to each participant the nature of the study, its purpose, the procedures involved, the expected duration, the potential risks and benefits and any discomfort it may entail. Each participant will be informed that participation to the study is voluntary and that he/she may withdraw from the study at any time and that withdrawal of consent will not affect his/her subsequent medical assistance and treatment. The participant will be informed that his/her medical records may be examined by authorised delegates other than their treating physician. All participants for the study will be provided a participant information sheet and a consent form describing the study providing sufficient information for participant to make an informed decision about their participation in the study. The participants will have enough time given to decide to participate or not (minimum of 2 days). The formal consent of a participant, using the approved consent form, will be obtained before the participant is submitted to any study procedure. The participant will have to read and consider the statement before signing and dating the informed consent form, and will have a copy of the signed document. The consent form will also be signed and dated by the investigator (or his designee) and will be retained as part of the study records.

2.8 Participant privacy and confidentiality

The investigators affirm and uphold the principle of the participant's right to privacy and will comply with applicable privacy laws. The anonymity of the participants will be guaranteed when presenting the data at scientific meetings or publishing them in scientific journals.

Individual subject medical information obtained as a result of this study is considered confidential and disclosure to third parties is prohibited. Subject confidentiality will be ensured by utilising subject identification code numbers to correspond to treatment data in the computer files.

For data verification purposes, authorised representatives of the Sponsor (-Investigator), a competent authority (e.g. Swissmedic), or an ethics committee are allowed to direct access to parts of the medical records relevant to the study, including participants' medical history.

2.9 Early termination of the study

The Sponsor-Investigator and the PI may terminate the study prematurely according to certain circumstances, for:

- In case of severe, life threatening or irreversible side effect grade IV (CTCAE V5.0) related to the study medication in >4 of the first ten patients or grade V (CTCAE V5.0) toxicity in >2 of the first ten patients, the investigators will close the study.
- Ethical concerns
- Insufficient participant recruitment.

The Sponsor may temporarily or permanently discontinue the study at the single site for safety, ethical, compliance or other reasons. If this is necessary, the Sponsor will endeavour to provide advance notification to the site. If the site or study is suspended or discontinued, the Investigator will be responsible for promptly informing the IRB/IEC. Where required by local regulations, the Sponsor will be responsible for informing the IRB/IEC of study or site discontinuation. In such cases, all study data and unused Nab-paclitaxel (Abraxane®) must be returned to the Sponsor.

2.10 Protocol amendments

PI, Local Site Investigators and Sponsors are allowed to amend the protocol or to provide suggestions for a protocol amendment. Substantial amendments will be only implemented after approval of the CEC and CA respectively. Under emergency circumstances, deviations from the protocol to protect the rights, safety and well-being of human subjects may proceed without prior approval of the sponsor and the CEC/CA. Such deviations shall be documented and reported to the sponsor and the CEC/CA as soon as possible. All Non-substantial amendments are communicated to the CA as soon as possible if applicable and to the CEC within the Annual Safety Report (ASR).

3. BACKGROUND AND RATIONALE

3.1 Background and Rationale

Rationale:

In this prospective open-label bi-centric phase IB clinical trial we will investigate the maximal tolerated dose (MTD) of Nab-paclitaxel (albumin-bound paclitaxel, Abraxane®) and cisplatin (Cisplatin-Teva®), both administered intraperitoneally by PIPAC in patients with peritoneal carcinomatosis (PC) of gastric, pancreatic, ovarian origin or primitive peritoneal mesothelioma. Indeed, platinum and/or taxanes compounds have shown antitumor efficacy as a palliative systemic treatment in these four types of cancer.

Preclinical reports have shown that Nab-paclitaxel (Abraxane®), a nanoparticle albumin-bound formulation of paclitaxel, has an enhanced antitumoral activity due to its internalization through macropinocytosis by the macrophages of the tumor environment (TAMs) leading to anti-tumoral immunomodulatory effect. We hypothesized that nab-paclitaxel could be a good candidate for intraperitoneal administration by PIPAC in patients with peritoneal carcinomatosis, as this route allows reduced systemic toxicity and increased intratumoral drug concentration. We are expecting that this local intervention might rebalance favorably the immune environment, lead to a prolonged local control and potentially a survival benefit. As PIPAC procedure is usually repeated three times. Thus, it would be a great opportunity to observe changes in the tumor mutational profile and the immunological responses of the microenvironment in vivo.

We are hoping to demonstrate that the administration of new chemotherapy components, in particular nanomedicine is safe and will pave the way for future development in association with novel immunotherapies.

Background:

Intraperitoneal dissemination of malignant tumors is a major problem in the management of digestive and gynecological cancers resulting in very poor prognosis and a rapid marked deterioration in the quality of life of these patients. Malignancies most likely to spread to the peritoneum include gastric (60%), pancreatic (50%), ovarian (70%), colorectal (35%), peritoneal mesothelioma and pseudomyxoma peritonei.

Maintaining the quality of life of patients in palliative oncology is of great importance. Surgical and/or systemic treatments (intravenous chemotherapy) have limited efficacy in the palliation of symptoms related to peritoneal carcinomatosis at the cost of systemic toxicities, which are usually significant. Intravenous chemotherapy efficacy is usually short lived due to poor penetration into the peritoneal cavity. The role of intraperitoneal chemotherapy (IP) is to maximize tumor penetration and optimize cell death while minimizing systemic toxicity. Intraperitoneal chemotherapy retains several advantages: i) the dose intensity delivered to peritoneal carcinomatosis increases as half-life is longer, ii) intraperitoneal concentration is higher and iii) systemic toxicity reabsorption is weaker than intravenous route (1). Intraperitoneal chemotherapy (IP) is a recommended treatment for epithelial ovarian cancer in combination with maximal cytoreductive surgery and is the standard treatment of pseudomyxoma peritonei (2-10) (11-13). It has also been studied in several cancers of digestive origin (14).

Pressurized intraperitoneal aerosol chemotherapy (PIPAC) is a new laparoscopic procedure used for the intraperitoneal application of a pressurized aerolization of chemotherapy which increases the therapeutic ratio of the substance administered between local and systemic concentrations by a 2-3-fold, resulting in an improvement of local efficacy and reduction of systemic toxicity. The application of repeated cycles at a four-six weeks' interval increases the efficacy compared to systemic treatments. Taking advantage of physical laws, PIPAC generates an artificial pressure gradient enhancing tissue drug uptake by convection and distributing drugs more homogeneously within the closed and expanded abdominal cavity.

PIPAC with cisplatin 7.5 mg/m^2 and doxorubicin 1.5 mg/m^2 (about 10 % of usual systemic, and intraperitoneal (HIPEC) dose, respectively) at 12 mmHg and 37 °C for 30 min q 28–42 days was first applied in 2011 in three end-stage patients with peritoneal metastasis (49). The application was well tolerated and superior pharmacological properties were confirmed with higher intratumoral concentration of the drug and lower systemic drug concentration. PIPAC caused almost no hepatic or renal toxicity and no local complication was recorded (50-52). Retrospective data showed that PIPAC applied without any concomitant cytoreductive surgery and using the same regimen of chemotherapy (cisplatin and doxorubicin) in women with recurrent, platinum-resistant ovarian cancer was safe and effective (53). A Phase II trial with low-dose doxorubicin and cisplatin administered with PIPAC in 53 women with recurrent, platinum-resistant ovarian cancer showed a clinical benefit rate (CBR) of 62 % and an objective histological regression rate of 76 % coupled with a low incidence of serious adverse events: 15 % Common Terminology Criteria for Adverse Events (CTCAE) grade 3 events, no grade 4 or 5 (54). Retrospective studies from patients with colorectal and gastric peritoneal carcinomatosis treated with PIPAC suggested similar benefits (55, 56).
Intraperitoneal administration of chemotherapy is reimbursed by a specific DRG. With a standardized surgical approach and dedicated safety checklist, PIPAC could be safely introduced in clinical routine with minimal learning curve (24). This novel procedure was shown to be feasible in most patients with refractory carcinomatosis of various origins. Several retrospective and five prospective trials have shown that PIPAC can be used safely. Intraoperative complications and postoperative morbidity rates were low. Surgery-related complications occurred in up to 12 per cent and postoperative morbidity was low (CTCAE

grade 3-5 events reported in 0-37%). Regarding technical feasibility, laparoscopic access and repeatability were 83-100 and 38-82% (25). PIPAC was followed by a modest and transitory inflammatory response that was commensurate to the disease extent. No hematological, renal or hepatic toxicity was observed even after repetitive administration (26). Quality of life and symptoms were not impacted by PIPAC treatment (27). The available evidence on PIPAC was scrutinized in a systematic review confirming that PIPAC treatment was feasible, safe and well tolerated (28). Preliminary good response rates call for prospective analysis of oncological efficacy. The overall tumor response ranged between 40 and 75% in peritoneal carcinomatosis of ovarian and gastric origin with 3 successive PIPAC treatments with cisplatin and doxorubicin. Current practice of this new drug administration method was studied within an international expert panel showing excellent standardization of PIPAC among expert centers opening the door for registries and multi-center studies (29). There are currently several ongoing trials reported on clinicaltrials.gov in addition to those already published such as *NCT03294252, NCT03172416, NCT03210298, NCT03280511, NCT02735928, NCT03100708, NCT03287375, NCT02604784, NCT03304210*. In the recently published phase I study conducted by the german group, aiming to report the MTD of cisplatin and doxorubicin administered intraperitoneally by PIPAC has found that dose level of 10.5 mg/m^2 and 2.1 mg/m^2 of cisplatin and doxorubicin respectively were feasible and well tolerated. MTD was not reached in this study. This study confirms the safety of PIPAC and the low systemic reabsorption of chemotherapy administered by this route. The authors did not report any significant hematologic toxicity, neither hepatic, neurological nor renal toxicity (30).

Nowadays doxorubicine, cisplatin, oxaliplatin, mitomycine C and irinotecan have been used with PIPAC (off-label). Classical chemotherapy components used intravenously for ovarian, gastric and pancreatic neoplasias belong to the taxane and platin cytostatic families. Taxanes, and in particular paclitaxel, represent an important class of antitumor agents available since 30 years and have proven to be effective in the treatment of a number of solid malignancies. The albumin-bound form of paclitaxel, nab-paclitaxel, has fewer side effects, shows increased tumor cell cytotoxicity, and patients have higher overall response rates, compared with equal doses of solvent-based paclitaxel in breast, non–small cell lung (NSCLC) and pancreatic cancers.

3.2 Investigational Product and Indication

Abraxane (Nab-paclitaxel) is a next-generation taxane that has been specifically designed to overcome the limitations of conventional paclitaxel formulations, including the barriers to effective drug delivery of highly lipophilic agents (31). It is an albumin-drug complex of about 130 nm in diameter and is distributed similarly to endogenous albumin. Nanoparticule within this size range accumulates in solid tumors due to the enhanced permeability and retention effect (EPR), which results from an aberrant and leaky tumor vasculature and the lack of poorly developed lymphatic drainage system in many solid tumors. By exploiting the natural interactions between albumin and the gp60/caveolin-1 receptor pathway, it has been associated with rapid and preferential delivery and accumulation of paclitaxel at the tumor site. Pharmacokinetic studies have shown that, compared with paclitaxel, nab-paclitaxel is associated with a 9-fold greater penetration of paclitaxel into tissues via transporter-mediated pathways, a 33% higher intratumoral drug concentration, a 10-fold higher mean maximal concentration of free paclitaxel, and a 4-fold lower elimination rate (32-34). It is currently approved intravenously for breast, non–small cell lung (NSCLC), and pancreatic cancers. It has been studied intravenously in three phases II clinical trials for recurrent ovarian cancer and in two phases II and a phase III trial for recurrent gastric adenocarcinoma.

3.3 Preclinical Evidence

Preclinical and recent clinical studies have shown that nab-paclitaxel has better penetration into the tumor cells due to its macropinocytosis internalization by macrophages of the tumor environment (TAMs). The accumulation of Nab-paclitaxel via albumin-binding proteins at the tumor cell membranes allows for diffusion of paclitaxel into the intracellular compartment and subsequent induction of tumor cell death (57). Macropinocytosis is a form of endocytosis in which a large fluid-filled vesicle is pinched off from the cell membrane and brought into the interior of the cell. This is particulary relevant as peritoneal carcinomatosis from tumors highly infiltrated by TAMs have an especially poor prognosis, this hold true for pancreatic, gastric and ovarian carcinoma (39, 58, 59). Tumor-associated macrophages (TAMs) may be polarized in two phenotypes: type M1 or type M2. TAMs of the M2 phenotype are known to promote tumor proliferation by suppressing anti-tumor immune reactions and inducing angiogenesis and are associated with a poor prognosis in numerous cancers (39). On the contrary, a ratio favoring type M1 TAMs confers a better prognosis (40). A new mechanism of action of nab-paclitaxel recently identified in preclinical models of pancreatic cancers is its capacity to polarize TAMs toward M1 activation state. Nab-paclitaxel–mediated M1 induction might result in a positive feedback signaling, further promoting uptake of drug and enhancing its M1-activating effects in autocrine and paracrine fashions (41).

In addition to a direct cytotoxic effect on cancer cells, nab-paclitaxel has immunostimulatory properties and might therefore improve the immune surveillance and the prognostic of peritoneal carcinomatosis. It is becoming more and more obvious that novel cancer immunotherapies may require « priming » for an enhanced and prolonged response.

3.4 Clinical Evidence to Date

Taxanes, and in particular paclitaxel, represent a major class of antitumor agents available for 25 years and have proven to be effective in the treatment of a number of solid malignancies. The albumin-bound form of paclitaxel, nab-paclitaxel, has fewer side effects, shows increased tumor cell cytotoxicity, and patients have higher overall response rates, compared with equal doses of solvent-based paclitaxel in breast, non–small cell lung (NSCLC) and pancreatic cancers. Nab-paclitaxel is approved for intravenously use in patients with metastatic breast cancer, non-small cell lung cancer and pancreatic cancer. It has been studied intravenously in three phases II clinical trials for recurrent ovarian cancer and in two phases II and a phase III trial for recurrent gastric adenocarcinoma and is currently investigated in metastatic colorectal cancer. Pancreatic, breast and NSCLC cancers, for which nab-paclitaxel is as approved treatment regimen, are highly infiltrated by TAMs, which as mentioned above, may contribute to the mechanism of action of this nanoparticle (41).

Current reports of nab-paclitaxel with check- point inhibitors are limited, but early data suggest that nab-paclitaxel-based therapy may augment the antitumor activity of immune-checkpoint inhibitors and may double or triple the overall response rate or act as a sensitizer for immune checkpoint blockade therapy (42) (43) (44). Supporting the immunomodulatory effect of nab-paclitaxel on TAM, a phase IB trial that combined Nab-paclitaxel with anti-PD-L1 atezolizumab in triple-negative breast cancer patients showed an unexpected response rate of 42% (43). As monotherapy, the response rate to atezolizumab in this disease did not exceed 10% (NCT01375842)(60). These data have been confirmed with the recent clinical phase III trial that has demonstrated that this association allows to triple the overall response rate in comparison of the use of immune-checkpoint inhibitors alone (45, 46). Early clinical trials have indicated that pancreatic tumors may be fully resistant to monotherapy with immune-checkpoint inhibitors. However, recent data from a mouse-model study demonstrated that this resistance could be overcome with a combination therapy that contained nab-paclitaxel (47, 48). Nab-Paclitaxel is currently being studied in combination with nivolumab or

pembrolizumab in pancreatic cancer, as well as with other checkpoint inhibitors (atezolizumab, durvalumab, and ipilimumab) in multiple trials of solid tumors. A phase IB combining nab-paclitaxel and atezolizumab in NSCLC patients demonstrated a overall response rate of 56% (including 25% of complete response) (44).

IP administration of paclitaxel is a standard therapy for advanced epithelial ovarian carcinoma in North America (61), but the IP administration of Nab-paclitaxel has been little studied. A recent phase I trial examined the maximally tolerated dose (MTD) of IP administered Nab-paclitaxel in peritoneal carcinomatosis in 27 patients with advanced peritoneal malignancies (gynecologic (n=14), gastro-intestinal (n=12) and 1 peritoneal mesothelioma). The reached MTD was 140 mg/m^2. IP administration of Nab-paclitaxel showed higher (~150 fold) peritoneal exposure to the drug compared with the plasma exposure with a low inter- and intra-patient variability. Plasma AUCs from IP Nab-paclitaxel are ∼ 50% of plasma AUCs from IV delivery of the same dose; thus patients are exposed to therapeutic systemic drug levels (35).

3.5 Dose Rationale / Medical Device: Rationale for the intended purpose in study (pre-market MD)

The choice of 10.5 mg/m^2 cisplatin dose has been based on the result of the recent phase I escalation dose of cisplatin and doxorubicin association administered IP by PIPAC (30). The MTD dose in the phase I of IP nab-paclitaxel administration by catheterism was 140 mg/m^2 (35), we are expecting MTD at a lower dose for this phase I as PIPAC has an enhanced activity and penetration than conventional IP treatment. Cisplatin 10.5 mg/m^2 body surface in 150 ml NaCl 0,9% (concentration of 1mg/ml) and nab-paclitaxel (Abraxane®) in 150 ml NaCl 0,9% at escalating doses will be applied sequentially through a nebulizer (Capnopen®, Capnomed GmbH, Villigendorf, Germany) to a high-pressure injector (Accutron HP-D Injecteur, Medtron AG) with a flow rate of 0.6-0.7 ml/s and a median droplet size of 11 (3-15) µm at ambient temperature and at a maximal upstream pressure of 300 psi intraperitoneally. Treatment will be maintained for 30 minutes after administration at a pressure of 12 mmHg.

PIPAC administration will be repeated q4 -6 weeks intervals for a total of 3 courses.

3.6 Explanation for choice of comparator (or placebo)

Not applicable (single group assignment).

3.7 Risks / Benefits

PIPAC procedure:

The benefits of the PIPAC technique are due to the ability to decrease the total systemic drug dosage while increasing the drug concentration in peritoneal cells. From a surgical point of view, the PIPAC procedure does not leave foreign material intra-abdominally, as it is done in the IP therapy with the catheter, thus avoiding the risk of migration, obstruction and infection. The risks of PIPAC are due to the surgical nature of the procedure. The operation is done under general anesthesia, which has a very low mortality risk of 1/200.000. Risks associated with PIPAC procedure are extremely low, and in all published studies there have been no major perioperative complications (CTCAE ≤ 4). Grade 3 toxicities described have been the following: trocar hernia, bowel obstruction, intraoperative bleeding and cystitis with urosepsis (54). Minor complications described have been abdominal pain, nausea, and hematoma. There have been no post-operative re-laparotomies, or mortality (52). There have been no acute or chronic toxicities. Because of the chemical peritonitis caused by the procedure, one can expect a slight leukocytosis with an increase in C-reactive protein (CRP), which usually peaks on day 2 and decreases by postoperative day 3. Liver function remains in the normal range, but a slight

increase of ASAT and bilirubine can be seen on day 0, with normalization by day 3. Recently published phase I trial on cisplatin and doxorubicin escalation dose has proven that PIPAC phase I escalation dose is feasible with association of two chemotherapy compounds and did not report any significant hematologic toxicity, neither hepatic, neurologic nor renal toxicity.

Nab-paclitaxel (Abraxane®):

The toxicity of Nab-paclitaxel administered by intravenous route is well known. The only data available to our knowledge on its administration by IP route are from a recent phase I study showing the feasibility and safety of this route of administration (35).

Cisplatin (Cisplatin Teva®):

Cisplatin has been currently administered by intraperitoneal route at higher concentrations than in this study.

3.8 Justification of choice of study population

This study is intended for patients with peritoneal carcinomas from neoplasias all known to be sensitive to platin and/or taxane chemotherapy, who are in a palliative situation, due to peritoneal metastatic spreading, but still in good shape and would offer them an additional therapy that might improve their quality of life and potentially their survival. The study population will include all voluntary patients aged > 18 years, psychologically and physically able to follow the trial procedures and to give a written informed consent, suffering from peritoneal carcinomatosis, with limited extra-peritoneal metastases from pancreatic, oesogastric, ovarian cancers or primitive peritoneal mesothelioma, for whom standard therapies have been exhausted, or not feasible, or having residual disease following first line of therapy. We will not include patients with colorectal peritoneal carcinomatosis since this neoplasia is less sensitive to platin and taxanes compounds.

4. STUDY OBJECTIVES

4.1 Overall Objectives

The purpose of this phase IB study is to evaluate the safety and tolerability of the IP administration of cisplatin and nab-paclitaxel by PIPAC and to estimate the MTD of the combination of cisplatin and nab-paclitaxel in treating PC. The study aims to identify a selected population who may benefit from this treatment and to assess the predictive relevance of radiological assessment of PCI by abdominal CT enterography when available. The trial will include a QoL assessment and translational research on the impact of locally administered nab-paclitaxel and cisplatin on intratumoral immune response (spatial distribution of immune cell subsets), gene expression profile (RNAseq), and evaluation of potentially predictive biomarkers (whole-exome sequencing, RNAseq, immune cell subsets).

4.2 Primary Objective

The study seeks primarily to determine the maximum tolerated dose (MTD) and the safety profile of nab-paclitaxel and cisplatin administered by pressurized intraperitoneal aerosol chemotherapy (PIPAC) in accordance to CTCAE criteria version 5.0.

4.3 Secondary Objectives

Secondary objectives are to evaluate i) the response rate, ii) the effect on intratumoral immune response in patients with peritoneal carcinomatosis (translational research), iii) any benefit in QoL, iv) the predictive relevance of radiological assessment of PCI by abdominal CT enterography when available, v) the reproducibility of CT-PCI score and its correlation with surgical PCI evaluation.

4.4 Safety Objectives

To assess short-term safety and tolerability of the IP association of cisplatin and Nab-paclitaxel administration by PIPAC.

5. STUDY OUTCOMES

5.1 Primary Outcome

The **main outcome** of this phase IB study is to determine the MTD of Nab-paclitaxel (Abraxane®) administered IP by PIPAC in concomitance with cisplatin. MTD of nab-paclitaxel (Abraxane®) administered IP by PIPAC in concomitance with cisplatin is defined as the lowest dose level at which ≥33% (≥ 2/6) of patients experience DLT in the first cycle of treatment in accordance to CTCAE criteria version 5.0 with the exceptions clarified in chapter 9.2.1. DLT is defined as any CTCAE Grade 3 or 4 adverse event determined to be possibly, propably or definitely related to nab-paclitaxel (Abraxane®) and cisplatin IP administration.

5.2 Secondary Outcomes

i) The objective histological regression and objective tumor response rate (OTR) will be assessed according to the new regression system for peritoneal cancer (PRGS, peritoneal regression grade score system) at D1 of PIPAC 2 and 3 cycles. Histologic regression will be assessed by pathologic review of repeated peritoneal biopsies proceeded during laparascopy before each PIPAC cycle, according to the new regression system for peritoneal cancer.

ii) The objective response rate (ORR) and the clinical benefit rate (CBR) are defined by RECIST version 1.1 criteria. The reproducibility of radiological PCI score will be assessed by an abdominal CT enterography at screening and EOT visit, when available, and will be correlated with surgical PCI evaluation.

iii) Any benefit in QoL assessed by EORTC QLQ-C30 Version 3.0 and visual analogic scale for pain (EVA) questionnaires before (D0) and after each cycle of PIPAC application (D10).

5.3 Safety Outcomes

Documentation of AE and SAE with predefined toxicity criteria will be applied using CTCAE version 5.0 criteria, documented before and after the first, second, and third course of treatment (D0/D10 of each cycle). Surgical complications will be assessed according to Clavien classification and comprehensive complication index (CCI).

5.4 Other Outcomes

We will explore possible correlation between pathology or molecular alterations and other individual variables investigated in this study (type of cancer, survival, progression disease, reponse to treatment etc.) These analyses will be done on biopsies collected at D1 C1 of PIPAC. Translational research: the effect on intratumoral immune response (TAMs ratio, PD-L1/2 expression and TILs infiltration) as assessed by multispectral IHC before each PIPAC cycle, including the evaluation of specific cell subpopulations (CD8, CD4 etc) and the protein expression of relevant immune checkpoint proteins (for example, PDL1). We have planned to perform DNA and RNA sequencing of samples obtained before treatment (D1, cycle 1) and during treatment (D1, cycles 2 and 3). Outcomes of interest include (a) the distribution of gene expression profiles, (b) the gene expression changes during treatment, (c) the gene expression differences between responders and non-responders, (d) mutational patterns (pathogenic variants, mutational signatures) predicting response, (e) sub-clonal changes in response to treatment, for example expansion of resistant clones and resistance mutations.

In a latter phase, pharmacokinetic analysis of plasmatic concentrations of nab-paclitaxel (Abraxane®) and will be performed at pre-dose, end of infusion, H1, H4 and 24 hours after the first PIPAC treatment for the two first patients treated for each dose level escalation.

6. STUDY DESIGN

6.1 General study design and justification of design

This phase IB study will enroll between 6 to 36 patients with PC of ovarian, gastric and pancreatic origin or primitive peritoneal mesothelioma for whom standard therapies have been exhausted or not feasible. It is a prospective sequential open-label non-randomized multicentric conventional phase IB with a single dose escalation of the investigational drug (nab-paclitaxel, Abraxane®) performed in association with a cisplatin dose (prespecified at 10.5 mg/m^2) administered intraperitoneally by PIPAC.

The study design consists in a modified Fibonacci sequence (Nab-paclitaxel dose increase by 100%, 67%, 50%, 40%, and 33% for all the rest): 7.5 mg/m^2, 15 mg/m^2, 25 mg/m^2, 37.5 mg/m^2, 52.5 mg/m^2 and 70 mg/m^2. Three patients are treated at each dose level (DL) until the first DLT in the first cycle of treatment (defined as grade 3 or 4, CTCAE version 5.0 with the exceptions clarified in chapter 9.2.1) occurred.

If one patient among the three of the first cohort experiences a DLT within 4 weeks from the first cycle of PIPAC, then an additional cohort of 3 patients is treated at the same DL. If no patient among 3 or 1 among 6 experiences a DLT within 4 weeks from the first cycle of PIPAC, the dose is escalated. The MTD is (maximal tolerated dose) defined as the lowest dose level at which ≥33% (≥ 2/6) subjects experienced a DLT during the first cycle of treatment (62).

Within each cohort, a timeframe after the first PIPAC procedure will be respected before starting the treatment of the next patient.

In the first cohort of a DL:

- 2 patients can be enrolled simultaneously in cycle 1, while the third patient should be included only after at least one of the previous patients has completed the DLT monitoring period without experiencing any DLT.
- In case only 1 patient was initially enrolled: The next 2 patients of the same cohort can be enrolled simultaneously only after the first patient has completed the DLT reporting period without experiencing a DLT. The next 2 patients of the same cohort should be enrolled sequentially, if the first patient experienced a DLT.

In the second cohort of a DL:

- If no DLT was experienced in the first cohort of 3 patients: 2 patients can be enrolled simultaneously, while the third patient should be included only when at least one of the previous patients has completed the DLT monitoring period without experiencing any DLT.
- If 1 DLT was experienced in the first cohort of 3 patients: the 3 planned patients of the second cohort should be enrolled sequentially.

6.2 Methods of minimising bias

We will use a validated questionnaire for the evaluation of QoL, filled by the patient itself. The response rate according to RECIST criteria version 1.1 will be determined through radiological review by dedicated radiologists in each implicated center. Adverse events will be assessed by the Principal Investigator or delegate.

6.2.1 Randomisation

Not applicable. Single group assignment.

6.2.2 Blinding procedures

Not applicable. Single group assignment.

6.3 Unblinding Procedures (Code break)

Not applicable. Single group assignment.

7. STUDY POPULATION

7.1 Eligibility criteria

Participants fulfilling all of the following **inclusion** criteria are eligible for the study:

- Informed Consent as documented by signature
- Age ≥18 years
- Who are psychologically able to follow the trial procedures
- With peritoneal carcinomatosis from pancreatic, oesogastric, epithelial ovarian cancers or primitive peritoneal mesothelioma
- Not candidate for surgical cytoreduction and IP/HIPEC based on expert multidisciplinary board
- Who received at least one line of chemotherapy and for whom standard therapies have been exhausted or not feasible. Patients with residual disease following the first line of therapy or following secondary debulking are eligible.
- ECOG 0, 1 or 2
- Life expectancy > 3 months

The presence of any one of the following **exclusion** criteria will lead to exclusion of the participant:

- Predominant extra-peritoneal metastases at the discretion of the study team after discussion at the multidisciplinary tumor board
- Bowel obstruction, clinically significant active gastro-duodenal ulcer or ongoing abdominal infection (bacterial, viral or fungal),
- Chemotherapy or surgery within the last two weeks prior to C1D1,
- General or local (abdominal) contra-indications for laparoscopic surgery
- Known allergy to cisplatin or other platinum-containing compounds or to compounds of similar chemical or biologic composition of nab-paclitaxel,
- Severe renal impairment (calculated GFR (CKD-EPI) < 50 mL/min/1.73 m^2)
- Myelosuppression (platelet count < 100.000/µl, hemoglobin < 9g/dl, neutrophil granulocytes < 1.500/ml)
- Prophylactic or therapeutic anticoagulant cannot be safely interrupted for the PIPAC procedures.
- Hepatic impairment (serum total bilirubin ≥ 1.5 mg/dl, AST or ALT >5 x ULN),
- Severe respiratory or neurologic impairment (grade 3),
- Severe myocardial insufficiency (NYHA class >2), recent myocardial infarction, severe arrhythmias,
- Pregnancy or breastfeeding, women who can become pregnant must ensure effective contraception,
- Known or suspected non-compliance, inability to follow the procedures of the study, e.g. due to language problems, psychological disorders, dementia, etc. of the participant,
- Has a history or current evidence of any condition, therapy, or laboratory abnormality that might confound the results of the trial, interfere with the subject's participation for

the full duration of the trial, or is not in the best interest of the subject to participate, in the opinion of the treating investigator.

7.2 Recruitment and screening

Recruitment of voluntary patient will be done during the oncological multidisciplinary tumor board (gyneco-oncology and gastro-intestinal oncology), the oncologic consultation and referral from private practice and other hospitals according to the inclusion and exclusion criteria stated in section 7.1.

7.3 Assignment to study groups

Not applicable. Single group assignement.

7.4 Criteria for withdrawal / discontinuation of participants

Patients will be informed that they have the right to withdraw from the study at any time, without prejudice to their medical care, and they do not need to provide justification for their withdrawal from the study. Any withdrawal must be fully documented in the eCRF and source documents, and should be followed up by the Investigator.

The Investigator may withdraw a subject at any time if this is considered to be in the subject's best interest.
Withdrawal is mandatory in the following situations:

- For subjects presenting grade IV toxicity lasting more than 28 days despite withholding treatment
- Any subject whose health or well-being may be threatened by continuation of the treatment
- Need to use a prohibited medication during the treatment period
- Patient is unable to comply >33% within the protocol for other reason than a DLT.
- Requests by the patient to be withdrawn from the study (withdrawal of informed consent)
- Requests by the Investigator to withdraw the patient from the study.

8. STUDY INTERVENTION

8.1 Identity of Investigational Products (treatment / medical device)

In this Phase IB study the intraperitoneal dose of cisplatin will not exceed 10.5 mg/m^2, corresponding to the highest tolerated dose level studied in previous phase I trial and to 10% of the systemic dose normally administered by intravenous route. Therefore, we do not expect different systemic toxicities then with conventional intravenous administration route.

1. **Nab-paclitaxel (Abraxane®)**

MARKETING AUTHORISATION HOLDER
Celgene GmbH
P.O Box
8048 Zürich
Switzerland

MARKETING AUTHORISATION NUMBER(S)
Number (swissmedic): 63182

DATE OF FIRST AUTHORISATION/RENEWAL OF THE AUTHORISATION
Date of first authorization (swissmedic): September 2014

2. **Cisplatin (Teva®)**

MARKETING AUTHORISATION HOLDER
Teva Pharma AG, Aesch

MARKETING AUTHORISATION NUMBER(S)
Number (swissmedic): 47998

DATE OF FIRST AUTHORISATION/RENEWAL OF THE AUTHORISATION
Date of renewal authorization (swissmedic): October 2009

3. **PIPAC**: Pressurized intraperitoneal aerosol chemotherapy

All devices used for this laparoscopic procedure have a CE marking. The nebulizer (Capnopen®, Capnomed GmbH, Villigendorf, Germany) and the high-pressure injector (Accutron HP-D Injecteur, Medtron AG) will be supplied by the Surgery Department of each center according to local standard procedures.

8.1.1 Experimental Intervention (treatment / medical device)

- **Nab-paclitaxel** (Abraxane®: nanoparticle albumin-bound paclitaxel) 5 mg/ml powder for injection (suspension) is an albumin nanoparticle form of paclitaxel with a mean particle size of approximately 130 nanometres.
- **Cisplatin** (Teva®): Cisplatin 0.5 mg/ml concentrate for solution for injection, is a cytostatic agent used in the treatment of various types of cancer. We will use commercial vials of 50 mg for this study.

8.1.2 Control Intervention (standard/routine/comparator treatment / medical device)

Not applicable.

8.1.3 Packaging, Labelling and Supply (re-supply)

- **Nab-paclitaxel** (Abraxane®) will be bougth directly from the market and will be stored and provided by the HUG pharmacy according to standard procedures. The packaging and labeling will be done in accordance with applicable local regulatory requirements.
- **Cisplatin** (Teva®) will be bougth directly from the market and will be stored and provided by the HUG pharmacy according to standard procedures. The packaging and labeling will be done in accordance with applicable local regulatory requirements.

8.1.4 Storage Conditions

Shelf life

- **Nab-paclitaxel** (Abraxane®)
  - Unopened vials: See label for EXP Date.
  - Stability of reconstituted suspension in the vial: After first reconstitution, the suspension should be filled into an infusion bag immediately. However, chemical and physical stability has been demonstrated for 24 hours at 2°C-8°C in the original carton, and protected from bright light. Alternative light-protection may be used in the clean room.

- Stability of the reconstituted suspension in the infusion bag: After reconstitution, the reconstituted suspension in the infusion bag should preferentially be used immediately and delivered to the clinical unit where it will be administered to the subject within a maximum of 4 hours and should be kept under 25°C. However chemical and physical stability has been demonstrated for the combined total shelf life of the reconstituted drug in the vial and in the infusion bag for 24 hours at 2°C-8°C, if protected from bright light.

- **Cisplatin (Teva®)**
  - Unopened vials: See commercial label for EXP Date.
  - Stability of reconstituted suspension in the vial: After dilution in infusion fluids, the product can be stored for at most 14 days at room temperature (15-25°C) under protection from light. Do not store diluted solutions in the refrigerator or freezer. Exposure to ambient light must be limited to at most 6 hours. If exceeding 6 hours, the bags must be thoroughly wrapped in aluminium foil in order to protect the contents from ambient light.

Special precautions for storage

- **Nab-paclitaxel** (Abraxane®)
  - Unopened vials: should not be opened and should be stored in the outer carton in order to protect from light, separately from any other product in a secure, limited access storage area (Pharmacy in HUG) and should be stored below 25°C.

- **Cisplatin (Teva®)**
  - Unopened vials: Store between 15 and 25°C. Do not refrigerate or freeze. Keep container in the outer carton in order to protect from light.

8.2 Administration of experimental and control interventions

8.2.1 Experimental Intervention

Nab-paclitaxel (Abraxane®) in 150 ml NaCl 0,9% at escalating doses (7.5 mg/m^2, 15 mg/m^2, 25 mg/m^2, 37.5 mg/m^2, 52.5 mg/m^2 and 70 mg/m^2) and cisplatin 10.5 mg/m^2 body surface in 150 ml NaCl 0,9% will be applied sequentially, starting with Nabpaclitaxel, through a nebulizer (Capnopen®, Capnomed GmbH, Villigendorf, Germany) to an intravenous high-pressure injector (Accutron HP-D Injecteur, Medtron AG) with a flow rate of 0.6-0.7 ml/s and a median droplet size of 11 (3-15) µm at ambient temperature and at a maximal upstream pressure of 300 psi intraperitoneally. Treatment has to be maintained at ambient temperature for 30 minutes after administration at a pressure of 12 mmHg.

PIPAC will be performed only by gynaecologic or gastrointestinal surgeon who has already completed a special training and will be repeated q4-6 week's intervals for a total of 3 courses procedure.

PIPAC procedure:

- Intervention under general anesthesia
- Antibiotic prophylaxis with commercial cephalosporine administered during anesthetic induction
- Introduction of a 5mm optic after insufflation with Hasson technique
- Insufflation capnoperitoneum at 12 mmHg and insertion of 1 trocar of 10/12 for the

nebulizer,1 trocar for the camera and 1 working trocar (ascites removal, biopsies, peritonectomy).
- Removal of ascites and documentation of the volume and cytology
- Documentation of the peritoneal carcinomatosis index (surgical PCI score and Fagotti score for ovarian carcinoma) (See Appendix C).
- 5 punch peritoneal biopsies in the 4 quadrants of the abdomen and 1 peritonectomy of several cm^2 (See Appendix C).
- Connection of the nebulizer (Capnopen®, Capnomed GmbH, Villigendorf, Germany) to a high-pressure injector (Accutron HP-D Injecteur, Medtron G) and insertion into the abdomen.
- Pressurized dose of cisplatin and nab-paclitaxel (Abraxane®) at escalating doses will be applied via the nebulizer and injector, with a flow rate of 0.6-0.7 ml/s
- Maximal upstream pressure of 300 psi.
- Treatment to be maintained for 30 minutes after administration at ambient temperature and a pressure of 12 mmHg.

PIPAC hospitalisation: The length of stay in hospital for the PIPAC procedure is about 4 days (62).

Protection of personnel:
- Documentation of tightness of the abdomen via maximum CO2 flow of 0-0.4 L/min corresponding to the body resorption of CO2.
- Operation to be performed in an operating room containing a ventilation system.
- Remote control of injection of chemotherapy
- Elimination of chemotherapy by exsufflation via a closed line over two sequential microparticle filters into the airwaste system of the hospital
- Retraction of the trocars and elimination in special adapted cytostatic bins
- Video recording of every procedure
- Check list to double-check safety measures before administration of cytostatics

8.2.2 Control Intervention

Not applicable. Single group assignment.

8.3 Dose / Device modifications

No dose modification is planned in this study.

8.4 Compliance with study intervention

No special strategies to improve adherence apart from the medical visits is planned. Non-compliant patient will be dropped out from the study.

8.5 Data Collection and Follow-up for withdrawn participants

Patients who prematurely withdraw from the study will be displayed and summarized by primary reason and treatment.

8.6 Trial specific preventive measures

- **Permitted Medicines**

Any medications others than those excluded by the protocol, considered necessary for the patient's welfare, and not interfering with the study medication may be given at the discretion

of the Investigator or delegate. The Principal Investigator or delegate will record all concomitant medications taken by the subject during the study from the date of signature of informed consent until the FU visit, in the appropriate section of the eCRF. Additionally, any unplanned diagnostic, therapeutic or surgical procedure performed during the study period must be recorded in the concomitant procedure section of the eCRF, including the date, indication, description of the procedures and their outcome.

- **Prohibited Medicines**

Participation in other clinical trials evaluating experimental treatments or procedures is not permitted. The following drugs are prohibited during the study and patients requiring such drugs are excluded for participation in the study, because of its potential interaction with Nab-paclitaxel treatment: No other chemotherapy, immunomodulating therapy, immunosuppressive drugs, and high dose steroids (>1 mg/kg of prednisone or equivalent dose). Systemic hormonal therapy or experimental medications will be prohibited, while patients are in the treatment period of the study.

The metabolism of paclitaxel is catalyzed by CYP2C8 and CYP3A4. Pharmacokinetic drug-drug interactions between nab-paclitaxel and inhibitors or inducers of CYP2C8 or CYP3A4 have not been evaluated in humans. As reported in the investigator's brochure, caution should be exercised when administering nab-paclitaxel concomitantly with medicines known to inhibit (eg, ketoconazole and other imidazole antifungals, erythromycin, fluoxetine, gemfibrozil, cimetidine, ritonavir, saquinavir, indinavir, nelfinavir, and clopidogrel) or induce (eg, rifampicin, carbamazepine, phenytoin, efavirenz, and nevirapine) either CYP2C8 or CYP3A4. For this reason and if possible, all inhibitors or inducers of CYP2C8 or CYP3A4 will be replaced by another drug without this mode of metabolism. Strong inhibitors or inducers of cytochrome P450 and P-glycoproteins will be not employed. If a replacement is not possible, the PI will decide whether or not the patient has to be withdrawn from the study (see Appendix E).

Hormone replacement therapy, inhaled steroids and low dose steroids (<1 mg/kg of prednisone or equivalent dose) are allowed. Any exception must be discussed with the PI of the study. If the use of these drugs cannot be avoided, the Investigator should contact the PI of the study to agree on its exceptional use concomitant to study drug.

8.7 Concomitant Interventions (treatments)

No experimental procedures will be permitted, while patients are in the treatment period of the study. Any exception must be discussed with the PI of the study. Irradiation of target lesions should be avoided if possible. Palliative radiation during treatment with PIPAC can be performed but should be discussed closely with the Investigator regarding the field to be treated, the type of radiation technique to be used as well as potential side effect of the radiation treatment. Their use should be recorded in the eCRF.

8.8 Study Drug accountability

Cisplatin and Abraxane used in this study will be provided by the Pharmacy of Geneva University Hospitals. Two weeks before the planned study treatment day, the site Investigator (CHUV) will confirm the date of the first treatment to the sponsor (HUG). Sponsor is responsible for providing nab-paclitaxel (Abraxane®) and cisplatin to the site for the whole treatment of the patient enrolled (3 cycles).

Nab-paclitaxel (Abraxane®) in 150 ml NaCl 0,9% at escalating doses and cisplatin 10.5 mg/m^2 body surface in 150 ml NaCl 0,9% will be prepared at the Pharmacy of each site. The final identification labels for the administered trial products will be performed by both pharmacies involved, depending on patient's enrollment.
Accountability records will be held by study Investigators according to the delegation list. The study Investigator is responsible for ensuring drugs accountability, including reconciliation and maintenance records. All the processes will be carefully recorded on the appropriate drug accountability forms and an accurate accounting will be available for verification by the monitor at each monitoring visit.

Accountability records will include:

- Confirmation of the product reception.
- The use of each dose by each patient.
- Dates, quantities, batch numbers, formulation prepared at the site, expiry dates and study numbers assigned to the patients.
- The doses provided to the patients as specified by the protocol/amendment(s)

Nab-paclitaxel (Abraxane®) and cisplatin that have been dispensed to a patient must not be re-dispensed to a different patient.

8.9 Return or Destruction of Study Drug / Medical Device

At the end of the study unused nab-paclitaxel and cisplatin will be destroyed according to local site procedure.

9. STUDY ASSESSMENTS

9.1. Schedule of assessments

See Study Schedule of Assessments on page 18-19

9.2 Assessment of outcome

9.2.1 Assessment of primary outcome

- **MTD** defined as the lowest dose level at which ≥ 33% (≥ 2/6) of patients experienced DLT in the first cycle of treatment in accordance to CTCAE version 5.0 criteria.
- **DLT** is defined as any of the following CTCAE 5.0 Grade 3 or 4 adverse event determined to be probably, possibly or definitely related to Nab-paclitaxel (Abraxane®) and/or cisplatin IP administration and assessed within 4 weeks after the first PIPAC treatment:

Hematologic DLTs

- Febrile neutropenia grade >3for more than 7 days
- Platelet count decreased grade 3 or 4 for more than 7 days
- Thrombocytopenia requiring transfusion

Non-hematologic DLTs

- Any grade ≥ 3 non-hematological trial treatment-related AE Exception: non-clinically significant non-hematological laboratory findings
- Any treatment-related AE that leads to a delay of treatment in the start of cycle 2 of > 14 days
- Abdominal pain grade ≥ 3 during more than 7 days and requiring opioides treatment. Pain will be estimated with visual analogic scale (VAS). The highest VAS value of the day taken in bed will be recorded in the eCRF.

Note: Adverse events related to the primary tumor, such as progression of the disease will not be considered as DLTs.

9.2.2 Assessment of secondary outcomes

- **Histological assessment (OTR)**: evaluated by multiple peritoneal biopsies in minimum 3 different regions of the peritonei membrane with degree of histological regression determined by a dedicated pathologist according to the new regression system for peritoneal cancer (PRGS, peritoneal regression grade score system). This procedure will be repeated at D1 of each PIPAC cycle (See Appendix D).
- **Radiological assessment (ORR, CBR)**: will be evaluated by a CT enterography and /or chest CT Scan (RECIST/GCIG criteria version 1.1) at screening and EOT visit (FU+/- 7). Surgical PCI score (Sugarbaker) determined by the surgeon at D1 and radiological CT-PCI assessed after each CT enterography, when available,by dedicated radiologists will be notified in the patient file and reported in the eCRF (See Appendix B and C).
- **Quality of life** (QoL): will be evaluated with the EORTC questionnaire QLQ-C30 Version 3.0 and the VAS for pain at each medical visit before and after each PIPAC cycle and at EOT visit (D0, D10, FU). The highest VAS value of the day taken in bed will be recorded in the eCRF.

9.2.3 Assessment of other outcomes of interest

- **Pharmacokinectics analysis**: will be assessed in a latter phase by dosage of free plasmatic concentrations of nab-paclitaxel (Abraxane ®) performed at pre-dose, end of infusion, H1, H4 and 24 hours after the first cycle of PIPAC for the two first patients treated for each dose level escalation. Condition of participant: to be fasting 12 hours before the PIPAC procedure (See Appendix F).
- **Pathology and molecular alterations**: pathology (CD8, PDL1, MSI). These analyses will be done on biopsies collected at C1D1 of PIPAC.
- **Translational research: the effect on intratumoral immune response** (See Appendix G for more details)
    - **Immune response:** assessment of immune infiltrate on biopsies collected at baseline and after each PIPAC procedure to investigate changes in tumor microenvironment such as depletion of TAMs or downregulation of PD-L1 and whether it correlates with histologic response. Evaluation will be performed through multiplexed IHC at D1 after each PIPAC cycle. Assessment of the presence of TAMs ($CD68^+$, $CD163^+$, $Tie2^+$), regulatory T cells ($Foxp3^+$), TILs ($CD8^+$), plasmacytoid dendritic cells ($BDCA2^+$), resident T cells ($CD103^+$), PD-L1/ PD1and other immune cells to be defined.
    - **Gene expression profile (RNAseq):** quantification of gene expression by RNAseq will be performed on formalin-fixed paraffin embedded (FFPE) tumor samples. Bioinformatic processing will be based on a standardised pipeline (bwa, edgeR). The downstream analysis includes unsupervised hierarchical clustering for the discovery of underlying subgroups, differential expression analysis of matched samples before and during treatment and differential expression analysis between responders and non-responders. Gene expression information can also be used to deconvolute immune infiltrates (for example, CIBERSORT, TIMER), supplementing the immunohistochemical estimates.
    - **Whole-exome sequencing:** whole-exome sequencing will be performed on blood and FFPE tumor samples obtained before and during treatment and processed on a standardized pipeline (bcbio) for the identification of pathogenic variants (mutations) and copy number alterations (CNVkit). Mutational signatures can also be derived from exome data and have been associated with

distinct biological processes, such as deficient DNA repair. We plan to compare the patterns of responders with non-responders in the hope of identifying candidate biomarkers. In addition, we will examine the changes that might have resulted from exposure to treatment, such as the expansion of potentially resistant clones.

9.2.3 Assessment of safety outcomes

Safety will be assessed by the surgeon at each medical visit before (D0) and after (D10) each PIPAC course and at the EOT visit by a dedicated oncology physician, with predefined toxicity criteria which will be documented according to the CTCAE version 5.0 criteria and consigned in the patient file and eCRF. Surgical complications will be assessed according to Clavien classification and comprehensive complication index (CCI) (See Appendix A).

Adverse events

Treatment Emergent Adverse events (TEAEs) and serious adverse events (SAEs) will be summarized by presenting the number and percentage of patients having any adverse event, having any event by body system and having each individual adverse event (incidence, relationship to Nab-PIPAC, severity according CTCAE version 5.0). Adverse events that result in death (other than disease progression), discontinuation or are serious will be presented separately. Any other information, e.g. time of onset, duration, resolution, action to be taken, assessment of intensity, relationship with study treatment will be listed for all patients.

Laboratory parameters

All laboratory results mentionned in the eCRF monitored at each planned visit which are not in line with the laboratory normal ranges and/or the CTCAE version 5.0 criteria will be summarized by presenting shift tables using normal ranges, by presenting summary statistics of raw data and changes from baseline values (mean, median, standard deviation, range) and by flagging of notable values in data listings.

Vital signs

Vital signs at baseline and change from baseline will be summarized by changes from baseline values (mean, median, standard deviation, range) and by flagging of notable values in data listings.

9.2.4 Assessments in participants who prematurely stop the study

Patients who will prematurely withdraw from the study will be displayed and summarized by primary reason and treatment.

9.3 Procedures at each visit

Sequence and duration of all study periods:

- **Screening phase (S, D-28):** will consist in checking that every candidate meets the inclusion criteria and does not have any exclusion criteria. The physician will explain the purpose, the design, the risk and benefit balance of the study and the necessity of a good compliance. All the procedures and tests for the screening phase mentioned in the flow chart must have been completed during the defined interval timeline of 28 following days.
- **Intervention phase (C1D1, C2D1 +/- 3, C3D1 +/-3):** During the intervention phase, each participant intends to have 3 cycles of PIPAC repeated at q4-6 week's intervals. Before each PIPAC cycle (D0), a pre-operative medical visit by the surgeon and laboratory tests will be performed. The estimated duration of the hospitalization is 4 days. After each cycle of PIPAC, a medical visit will be done at day 10 (D10) to evaluate the toxicity and report any Treatment Emergent Adverse events assessed by using CTCAE criteria version 5.0, monitoring of vital signs and laboratory parameters and

filling the QoL questionnaires (QLQ-C30 Version 3.0 and VAS scale). The highest VAS value of the day taken in bed will be recorded in the eCRF.

- **End of treatment visit (EOT, CXD28 +/-7)**: will consist in a medical visit done within 28 days after the last PIPAC cycle and prior to the next anticancer treatment, with assessment of clinical and biological parameters, chest and CT enterography when available. Treatment Emergent Adverse events and QoL evaluation will be consigned by the physician in patient's file.
- **Data analysis and submission for publication**: within 1 year from the date of the EOT visit of the last patient recruited.
- **Expected duration of participant's participation:** from the screening phase till the EOT visit, the total participation time will be 6-8 months.
- **Study limitations:** Within this population, the DLT assessment will be difficult to distinguish from tumor associated symptoms. Selection of patients might create a bias.

9.3.1 Screening

Screening (baseline) and ICF records will be held by study Investigators according to the delegation list. Patient information consent will be collected before or the day of the screening visit, in any case before starting study procedures. It is mandatory that the written inform consent has been signed and dated to consider the patient as enrolled in the study.

Patient's file will be created in the eCRF on the day of the ICF signature in order to obtain a Unique Patient Number (UPN).

All pre-treatment screening procedures must be completed within 28 days prior to the registration, including:

- Demographics data
- Baseline disease characteristics, including diagnosis (histology proven) with main pathological characteristics, status of disease activity, previous treatments and responses
- Complete medical history
- Adverse event and concomitant medicationfrom the consent signature
- Inclusion/exclusion criteria review
- Complete physical examination including neurological examination with pallesthesia, abdominal circumference and vital signs (See Appendix H)
- ECOG performance status
- Tumor pain (intensity, site, frequency, analgesic use) and VAS scale.
- Assessment of tumor burden according to RECIST criteria version 1.1 and complete tumor staging performed by chest and abdominal CT-scan or PET-CT scan to rule out parenchyma metastatic disease
- Abdominal CT enterography when available, CT-PCI will be assessed and reported in the eCRF (See Appendix B)
- Laboratory tests **within 10 days** prior to the registration (see Appendix H):
    - Hematology
    - Serum chemistry
    - Coagulation
    - Serological tumor marker analysis when applicable
    - Urine analysis (dipstick). For pre-peri-menopausal females a urinary or serum pregnancy test (βhCG) must be performed to exclude pregnancy **within 7 days** prior to the registration. If the urine test is positive, a blood test (bHCG) has to be done to confirm the result. This is not required in females who are post-menopausal or surgically sterile

- Electrocardiogram (ECG)
- Surgical medical pre-operatory visit

When all screening procedures are completed and inclusion/exclusion criteria assessed, the patient will be registered in the eCRF as either included or screening failure. In case of screening failure, the reason must be entered in the eCRF. The first treatment should start within 7 days of the registration.

Patient characteristics assessed during the screening phase will be tabulated for visual comparison. For quantitative variables, the following descriptive statistics will be given: N, Mean and 95% confidence interval, Standard Deviation, Median and Interquartile range (for non-normally distributed); for qualitative variables, the Frequency and Percentage of patients within each category will be provided.

9.3.2 Intervention phase:

CYCLE 1

D0: Medical Visit by the surgeon the day before the PIPAC treatment (in respective centers)

- Cocomitant events and medication
- ECOG performance status
- Pain (intensity, site, frequency, analgesic use) grading according to CTCAE version 5.0 and VAS scale
- QoL questionnaires (EORTC QLQ-C30 Version 3.0) collected by the dedicated physician
- Physical examination and vital signs (see Appendix H)
- Laboratory tests (see Appendix H)▪:
    - Hematology
    - Serum chemistry
    - Serological tumor marker analysis when applicable (C2D0 and C3D0)
    - Pregnancy test if needed
- Mandatory pre-operative anesthesic visit
- Blood sample 3ml for translational research only C1D0 (See Appendix G)

▪ Labs performed within 7 days of treatments initiation during screening do not need to be repeated at D0 unless clinically indicated.

D1: Nab-PIPAC treatment and Hospital Stay

The PIPAC procedure will be performed only by a gynaecologic or gastrointestinal surgeon at CHUV/HUG who has completed the PIPAC training. The surgeon will determine the surgical PCI and/or Fagotti score, the volume of ascite if present and notify it on the operating procedure report recorded in patient's file. Then, he will proceed to collecting biopsy samples and administration of cisplatin and nab-paclitaxel through the PIPAC procedure (See Appendix C).

Tissular histopathological samples will be collected by the surgeon and sent to each histopathological center (CHUV/HUG). Three peritoneal biopsies and a several cm^2 peritonectomy will be needed for PRGS assessment. Two peritoneal biopsies will be collected for translational reasearch (IHC and molecular biology analysis). (See Appendix G). Ascites volume will be noted before each PIPAC procedure.

<u>Daily visits with the following assessment are mandatory during the hospital stay (D1-D3), including:</u>

- Physical examination and vital signs (See Appendix H)
- Adverse events and concomitant medications
- Pain (intensity, site, frequency, analgesic use) grading according to CTCAE version 5.0 and VAS scale.

Beyond the expected duration of hospitalization, follow-up will be performed as per standard of care or at the local investigator's discretion. If hospitalization exceed 4 days, SAE must be reported to the sponsor.

D10 (+/- 3): Medical Visit

- Adverse events will be graded by the local site Investigator with concomitant medication, anamnestical and physical relevant features
- ECOG performance status
- Pain (intensity, site, frequency, analgesic use), grading according to CTCAE version 5.0 and VAS scale
- Clavien-Dindo classification and comprehensive complication index assessment (See Appendix A)
- Physical examination and vital signs (See Appendix H)
- Laboratory tests (see Appendix H):
  - Hematology
  - Serum chemistry
- QoL questionnaires (EORTC QLQ-C30 Version 3.0) collected by the dedicated physician
- Audiogram will be performed only in case of new clinical symptoms

CYCLES 2 and 3

Each visit mentioned in cycle 1 (D0, D1 +/-3, D2 and thereafter during hospitalisation and Medical Visit at D10) will be repeated for PIPAC cycles 2 and 3.

When available, a CT enterography will be performed within 3 days before C3D0 in order to ensure the assessment of CT-PCI score before the next PIPAC administration.

For patients in the study for whom the treatment is effective, the visit of the cycle 3 will be repeated as long as it is necessary and reported in the e-CRF.

9.3.3 Short-term Follow-up Visit (End-of-treatment visit)

EOT CXD56 (+/- 7)

- Adverse events will be graded by the local site Investigator or an official delegate physician and recensed in each patient file with concomitant medication, anamnestical and physical relevant features
- ECOG performance status
- Pain (intensity, site, frequency, analgesic use) grading according to CTCAE version 5.0 and VAS scale (See Appendix D).
- Physical examination and vital signs (See Appendix H)
- Laboratory tests (see Appendix H):
  - Hematology
  - Serum chemistry

    - Serological tumor marker analysis when applicable
- QoL questionnaires (EORTC QLQ-C30 Version 3.0) collected by the dedicated physician.
- Assessment of tumor burden by chest and abdominal CT enterography, CT-PCI will be assessed and reported in the eCRF. Response will be assessed using the RECIST criteria version 1.1

9.3.4 Follow-up Visits (+/- 14) (each 3 months for 5 years)

- Disease and survival status

10. SAFETY

10.1 Drug studies

During the entire duration of the study, all serious adverse events (SAEs) will be collected, fully investigated and documented in source documents and electronic case report forms (eCRF). In addition all TEAEs will be reported in the source document and electronic CRF. Study duration encompassed the time from when the participant signs the informed consent until the end of treatment visit.

10.1.1 Definition and assessment of (serious) adverse events and other safety related events

An **Adverse Event (AE)** is any untoward medical occurrence in a patient or a clinical investigation participant administered a pharmaceutical product and which does not necessarily have a causal relationship with the study procedure. An AE can therefore be any unfavourable and unintended sign (including an abnormal laboratory finding), symptom, or disease temporally associated with the use of a medicinal (investigational) product, whether or not related to the medicinal (investigational) product. [ICH E6 1.2]

A **Treatment Emergent Adverse Events (TEAE)** are undesirable events not present prior to medical treatment, or an already present event that worsens either in intensity or frequency following the treatment.

A **Serious Adverse Event (SAE)** is classified as any untoward medical occurrence that:

- Results in death other than disease progression (grade 5), i.e. the AE causes or contributes to the death. Death is usually considered to be the outcome of an event; the event that leads to death <u>other than disease progression</u> is defined as the SAE.
- Is life-threatening, i.e. the AE places the patient at risk of death at the time of the event; the definition does not apply to an AE that hypothetically might cause death if it were more severe.
- Requires in-patient hospitalization at least a 24-hour (other then the study planned hospitalizations) or prolongation of existing hospitalisation beyond the expected length of stay more than the attended 4 days (70),
- Results in persistent or significant disability/incapacity, i.e. the AE resulted in a substantial disruption of the subject's ability to conduct normal activities.
- Is a congenital anomaly/birth defect, i.e. an adverse outcome in a child or fetus of a subject exposed to the NAB-PIPAC before conception or during pregnancy.

In addition, important medical events that may not be immediately life-threatening or result in death, or require hospitalisation, but may jeopardise the patient or may require intervention to

prevent one of the other outcomes listed above should also usually be considered serious. [ICH E2A].

Pregnancy, while not be considered SAEs, shall be reported as SAEs for monitoring and follow-up purposes.

If any of these events occur during the study, including during the SAE follow-up period, the event must be reported to the principal investigator and the sponsor designated safety monitoring body and to the Sponsor without delay. The SAE report form should be used to report the event, even though the event is not considered a SAE in itself. Pregnancies should be followed up until delivery, and the sponsor designated safety monitoring body informed of the outcome.

SAEs should be followed until resolution or stabilisation. Participants with ongoing SAEs at study termination (including safety visit) will be further followed up until recovery or until stabilisation of the disease after termination.

Assessment of Causality
Both Investigator and Sponsor-investigator make a causality assessment of the event to the study drug, based on the criteria listed in the ICH E2A guidelines:

Relationship	Description
Definitely	An adverse event that occurs during or shortly after administration of the investigational agent that follows a known response pattern to the investigational agent, that improves after stopping the investigational agent and that reappears after repeated exposure to the investigational agent (rechallenge). Depending on the nature of the adverse event, rechallenge may not be possible, in which case only a probable relationship can be established.
Probably	An adverse event that occurs during or shortly after administration of the investigational agent that follows a known response pattern to the investigational agent that improves after stopping the investigational agent, and that cannot be reasonably explained by the known characteristics of the patient's clinical state or by other therapies.
Possibly	An adverse event that occurs during or shortly after administration of the investigational agent, that follows a known response pattern to the investigational agent, but that could have been produced by the patient's clinical state or by other therapies.
Unlikely	An adverse event for which the temporal sequence is unlikely to have had any reasonable association with the event and/or the event could have been produced by the patient's clinical state or by other therapies.
Not related	An adverse event for which sufficient information exists to indicate that the etiology is unrelated to the investigational agent. Another etiology must be specified.
Unsufficient data	Sufficient information for judgment not available at this time (e.g. insufficient evidence, conflicting data or poor documentation). Followup necessary.

*Improvement after dechallenge only taken into consideration, if applicable to reaction

Unexpected Adverse Drug Reaction
An "unexpected" adverse drug reaction is an adverse reaction, the nature or severity of which is not consistent with the applicable product information (See Appendix A). [ICH E2A]

Suspected Unexpected Serious Adverse Reactions (SUSARs)
The Sponsor-Investigator evaluates any SAE that has been reported regarding seriousness, causality and expectedness. If the event is related to the investigational product and is both serious and unexpected, it is classified as a SUSAR.

Assessment of Severity
The grades for severity described in the "Common Terminology Criteria for Adverse Events CTCAE Version 5.0" terminology will be used (See Appendix A).

10.1.2 Reporting of serious adverse events (SAE) and other safety related events

Reporting of SAEs
All SAEs or new information on a previously reported SAE (irrespective of suspected cause) must be reported by fax by the Investigator immediately and within a maximum of <u>24 hours</u> of discovery to the Sponsor-Investigator of the study. The Sponsor-Investigator will re-evaluate the SAE and return the form to the site. SAEs must be recorded in the AE section of the Case Report Forms.
SAEs resulting in death are reported to the local Ethics Committee (via local Investigator) <u>within 7 days</u>. The other Ethics Committees involved in the trial receive SAEs resulting in death in Switzerland via Sponsor-Investigator <u>within 7 days</u>.
Exemptions from expedited reporting may be possible if the SAE is either a clear result of the underlying disease.

Reporting of SUSARs
A SUSAR needs to be reported to the local Ethics Committee (local event via local Investigator) and to Swissmedic (via Sponsor-Investigator) <u>within 7 days</u>, if the event is fatal, or <u>within 15 days</u> (all other events).
The Sponsor-Investigator must inform all Investigators participating in the clinical study of the occurrence of a SUSAR. All in the trial involved Ethics Committees will be informed about SUSARs in Switzerland via Sponsor-Investigator according to the same timelines.

Reporting of Safety Signals
All suspected new risks and relevant new aspects of known adverse reactions that require safety-related measures, i.e. socalled safety signals, must be reported to the Sponsor-Investigator within 24 hours. The Sponsor-Investigator must report the safety signals <u>within 7 days</u> to the local Ethics Committee (local event via local Investigator) and to Swissmedic.
The Sponsor-Investigator must immediately inform all participating Investigators about all safety signals. The other in the trial involved Ethics Committees will be informed about safety signals in Switzerland via the Sponsor-Investigator.

Reporting of Health hazards
If immediate safety and protective measures have to be taken during the conduct of a clinical trial, the investigator shall notify the ethics committee of these measures, and of the circumstances necessitating them, within 7 days. [ClinO Art. 37].

Reporting and Handling of Pregnancies
Pregnant participants must immediately be withdrawn from the clinical study. Any pregnancy during the treatment phase of the study and within 30 days after discontinuation of study medication will be reported to the Sponsor-Investigator within 24 hours. The course and outcome of the pregnancy should be followed up carefully, and any abnormal outcome regarding the mother or the child should be documented and reported.

Periodic reporting of safety
An annual safety report containing information from all sites is submitted once a year to the local Ethics Committee via local Investigator and to Swissmedic via Sponsor-Investigator.

Device deficiencies
Conforming to the Art. 15 of the MedDO (SR 812.213), any professional person who becomes aware of an incident when using medical devices must report this to the Agency. The report may be submitted by a professional association (internal reporting system of each hospital involved).

10.1.3 Follow up of (Serious) Adverse Events

All participants terminating the study (either regularly or prematurely) with reported ongoing SAE, or any ongoing AEs of laboratory values or of vital signs being beyond the alert must be documented and followed up until the event is either resolved, subsided, stabilized, disappeared or is otherwise explained or the study subject is lost to follow-up. All follow-up activities have to be reported, if necessary on one or more consecutive SAE report forms in a timely manner. All fields with additional or changed information must be completed and the report form should be forwarded to the Study Contact for Reporting Serious Adverse Events as soon as possible but within 7 calendar days after receipt of the new information. Clinically significant laboratory abnormalities will be followed up until they have returned to normal, or a satisfactory explanation has been provided. Follow-up reports relative to the subsequent course of an adverse event noted for any subject must be submitted to the Sponsor.

In case of lost to follow-up of participants, even if a SAE form cannot be fully completed at this stage, the following minimum information is required as initial notification:

- Patient's identification details
- PIPAC administration details
- Diagnosis of the event with the description (or event description if the diagnosis is not available) and the date of its occurrence
- Reason for considering the event serious
- Relationship of the event to the PIPAC
- Action taken and outcome
- Name, address and phone number of Investigator

11. STATISTICAL METHODS

A delegated biostatistician will perform data management and the pre-planned statistical analysis. A safety report will be performed 16 weeks after the last eligible patient has completed the last third cycle of PIPAC. The final efficacy analysis will be performed one year after the last eligible patient has completed the last follow-up visit (EOT visit). A final report will be issued at the end of the trial.

11.1 Hypothesis

Not applicable.

11.2 Determination of Sample Size

An estimated number of 6 to 36 patients is expected to determine the MTD for this phase IB study with a modified Fibonacci escalation 3+3 design.

11.3 Statistical criteria of termination of trial

The trial will end in case of more than one grade 5 event related to the Investigational product or to the study procedure (CTCAE version 5.0). Deaths due to progressive disease are not considered as grade 5 events.

11.4 Planned Analyses

A planned analysis will be performed within one year after the short-term follow-up visit of the last participant.

11.4.1 Datasets to be analysed, analysis populations

Intent-to-treat (ITT) analysis will be performed on all patients who receive at least two cycles of PIPAC.

11.4.2 Primary Analysis

The intended primary analysis will be done within one year after study completion (last EOT visit). The statistical analysis will be conducted by a dedicated biostatistician.

11.4.3 Secondary Analyses

Not applicable.

11.4.4 Interim analyses

Not applicable.

11.4.5 Safety analysis

The safety parameters will be consigned in the eCRF by the Investigator or delegate at each visit mentioned in the flow shart (9.1).
The safety endpoints in this study are described as:
Adverse and serious adverse events (incidence, relationship to nab-paclitaxel, severity according CTCAE version 5.0) will be summarized by presenting the number and percentage of patients having any adverse event, having any event by body system and having each individual adverse event.
Adverse events that result in death, discontinuation or are serious will be presented separately.
Any other information, e.g. start and end date, severity of adverse events or relatedness to study medication will be listed for all patients.
Patients who prematurely withdraw from the study will be displayed and summarized by primary reason and treatment.
Laboratory values: All laboratory results which are not in line with the laboratory normal ranges and/or the CTCAE version 5.0 criteria will be summarized by presenting shift tables using normal ranges, by presenting summary statistics of raw data and changes from baseline values (mean, median, standard deviation, range) and by flagging of notable values in data listings.
Vital signs at baseline and change from baseline will be summarized by changes from baseline values (mean, median, standard deviation, range) and by flagging of notable values in data listings.

11.4.6 Deviation(s) from the original statistical plan

No deviation(s) from the planned analyses will be justified.

11.5 Handling of missing data and drop-outs

Attempts will be made to complete any missing data. In addition, the means and ranges of all variable distributions and outlying data or improbable combinations of variables will be examined before analysis is undertaken. Queries will be sent to investigators whenever inconsistencies, outlying or missing data occurred.

Any crucial analysis not described in the statistical analysis plan will be specified and justified in the final clinical report.

12. QUALITY ASSURANCE AND CONTROL

The Sponsor is responsible for implementing and maintaining quality assurance and quality control systems with written Standard Operating Procedure (SOPs) and Working Instructions, at all sites in case of multicentre studies. The Principal Investigator is responsible for proper training of all involved study personnel.

12.1 Data handling and record keeping / archiving

Data (eCRF) will be collected and archived by study coordinator in electronic form. The study will strictly follow the protocol. If any changes become necessary, they must be laid down in an amendment to the protocol. All major amendments of the protocol must be signed by the Sponsor Investigator and submitted to IEC and Swissmedic.

12.1.1 Case Report Forms

The investigators and/or authorized designees will use electronic case report forms (eCRF), one for each enrolled study participant, to be filled in with all relevant data pertaining to the subject during the study. All subjects who either entered the study or were considered not-eligible or were eligible but not enrolled into the study additionally have to be documented on a screening log. The investigators and/or authorized designees will document the participation of each study subject on the Enrollment Log.
For each enrolled study participant an eCRF will be maintained. eCRFs must be kept current to reflect subject status at each phase during the course of study. Participants will not be identified in the eCRF by name or initials and birth date. Appropriate coded identification with participant number in combination with the site and study identification must be used. The Principal Investigator or designee is authorized for which eCRF entries and will assure that any authorised person will be identified.

12.1.2 Specification of source documents

Source data will be directly recorded in the eCRF but will also be available in the informatic patient file at the site to document the existence of the study participants and visits. Source data will include the original documents relating to the study, as well as the medical treatment and medical history of the participant. All data entered into the eCRF must also be available in the informatic patient file either as a letter or as notes taken by either the investigator and/or authorized designees. These records will include the following: originals or copies of laboratory and other medical test results. Documented medical histories and narrative statements relative to the subject's progress during the study will be maintained.

It is the responsibility of the investigators to assure to perform a complete and accurate documentation of the subject data in the course of the study will be entered completely and correctly in the respective data base. Corrections in the eCRF may only be done by the investigator and/or authorized designees. In case of corrections the original data entries will be archived in the system and can be made visible. For all data entries and corrections date, time of day and person who is performing the entries will be generated automatically. A list with

signatures and initials of all authorized persons will be filed in the study site file and the trial master file, respectively.

12.1.3 Record keeping / archiving

All study data must be archived for a minimum of at least 10 years after study termination or premature termination of the clinical trial (VKlin Art. 25). The Investigator will retain copies of the essential documents for the period of at least 10 years as specified by GCP. The data storage will be done at the DFDL unit (HUG) and Phase I Unit (CHUV). Essential documents defined by ICH-GCP include the signed protocol and any amendment(s), copies of the completed eCRFs, signed Informed Consent Forms (ICF) from all patients who consented, hospital records, quality of life questionnaires and other source documents, IRB/IEC approvals and all related correspondence including approved documents, drug accountability records, study correspondence and a list of the patients' names and addresses. The Investigator will inform the Sponsor of the storage location of the essential documents. All data documentation will be kept in dedicated locked cupboards in DFDL to prevent accidental or premature destruction of these documents.

12.2 Data management

Data management will be performed by UIC (Unité d'Investigation Clinique), a unit which is part of CRC (Centre de Recherche Clinique / CTU) at HUG (Hôpitaux Universitaires de Genève) and the Faculty of Medicine UNIGE (Geneva University). UIC is certified ISO 9001/2008, and the unit guarantees best practices in the field of clinical data management. Data are physically stored in a relational database management system, using a dedicated clinical database management system software [secuTrial®].

12.2.1 Data Management System

The Investigator or designee will be responsible for recording study data in the eCRF and to ensure their accuracy. The eCRF is the tool used to collect data from each participating site. The UIC will be responsible for the development and testing of the eCRFs in collaboration with the sponsor team. Data are physically stored in a relational database management system [Oracle ver.11g], using a dedicated clinical database management system software [secuTrial®].

Data protection is insured by the use of secuTrial(TM), a certified German data management application compliant with the requirements of the EU standards in clinical trials, as well as with the Swiss clinical trial requirements. SecuTrial is an Oracle-based software for capture and management of Clinical Trial data. SecuTrial(TM) comprises separate "modules" with distinct roles and identifications, such as administration, eCRF set-up, data capture, review, monitoring, data management, including data safety and trackback (personal identification, highly secured password protection, inactivity logging-out, activity log, detailed audit trails, etc.), and generation of up-to-date reports and statistics. The data management with SecuTrial(TM) provides a framework of data protection and security measures designed to guarantee that all data are safe from unforeseen, unintended, unwanted or malevolent use. This ensures the right of any individual, expecting that personal information will be processed securely and will not be disseminated in any form without their written consent.

Data management will be undertaken at the UIC in Geneva. SOPs are already in place for the collection and handling of data received at the Unit.

12.2.2 Data security, access and back-up

Health related personal data captured during this project are strictly confidential and accessible only by investigators and authorized personnel; disclosure to third parties is prohibited. Coding

will safeguard participants' confidentiality. Data generation, transmission, storage and analysis of health related personal data within this project will strictly follow the current Swiss legal requirements for data protection.
An eCRF will be utilized; no formalized back up is required.
Electronic data protection (eCRF), backup and archiving are under the responsability of the UIC of CRC at HUG, with the collaboration of the HUG Department of Information System.
All data collection forms and databases from the trial are the exclusive property of sponsor.

12.2.3 Analysis and archiving

The data will be entered into a validated database secuTrial(TM). The statistical analysis will be conducted by a dedicated biostatistician. A delegate from the UIC will be responsible for data processing, in accordance with Sponsor data management procedures. Database lock will occur once quality assurance procedures have been completed.

Data management is performed by the UIC. HUG data protection, backup and archiving is provided by Department of Information System, the information system direction department, which is separate from CRC.
Copies of the essential documents for the period will be archived for at least 10 years at the DFDL unit (HUG). The eCRF will be archived by the CRC for at least 10 years. The Investigator will inform the Sponsor of the storage location of the essential documents, and must contact the Sponsor for approval before disposing of any.

12.2.4 Electronic and central data validation

Validation of the eCRF and database will be done by the UIC at HUG.

12.3 Monitoring

The Investigator must ensure that eCRFs are completed in a timely manner and must allow a Sponsor representative periodical access to eCRFs, subject records and all study related materials. A risk-based monitoring will be conducted by the UIC and the frequency of monitoring visits will be determined by factors such as the frequency of subject visits and the site enrolment rate. In order to verify that the study is conducted in accordance with ICH-GCP, regulatory requirements, and the study protocol and that the data are authentic, accurate and complete, the Sponsor representative will review CRFs and other study documents and will conduct source data verification. Upon study completion, the Sponsor representative will visit the site to conduct a study termination visit. The source data/documents will be accessible to monitors and questions will be answered during monitoring. This will involve collection of any outstanding documentation.

12.4 Audits and Inspections

In compliance with GCP and regulatory requirements, the Sponsor, Swissmedics, or regulatory agencies or IRB/IECs may conduct quality assurance audits at any time during or following a study. The Investigator will agree to allow auditors direct access to all study related documents including source documents, and must agree to allocate his or her time and the time of his or her study staff to the auditors in order to discuss findings and issues. During the audits, the participant data will be kept strictly confidential.

12.5 Confidentiality, Data Protection

Data generation, transmission, archiving and analysis of personal data within this study, strictly follows Swiss legal requirements according to the federal law on data security, as well as the regulation on professional secrecy in clinical research. Prerequisite is the voluntary approval of the subject given by signing the informed consent prior start of participation of the clinical trial.

Individual subject medical information obtained as a result of this study is considered confidential and disclosure to third parties is prohibited. Subject confidentiality will be further ensured by utilising subject identification code numbers to correspond to treatment data in the computer files. Such medical information may be given to the subject's personal physician or to other appropriate medical personnel responsible for the subject's welfare, if the patient has given his/her written consent to do so. Data generated as a result of this study are to be available for inspection on request by the monitors, by the IEC and the regulatory health authorities

The Investigator will ensure that the patients' anonymity is maintained. On the eCRFs or other documents submitted to the Sponsor, patients will be identified by their assigned identification number. If subject names are included on copies of documents submitted to the Sponsor or the Sponsor's designee, the names will be obliterated and the assigned subject numbers added to the documents.

The Investigator will keep a separate log of patients' identification numbers, names, addresses, telephone numbers and hospital numbers. Documents not for submission to the Sponsor, such as signed Informed Consent Forms (ICF), will be maintained in strict confidence by the Investigator. The investigators are liable to treat the entire information related to the study and the compiled data strictly confidentially. Any passing-on of information to persons that are not directly involved in the study must be approved by the owner of the information.

12.6 Storage of biological material and related health data

The translational research samples will be performed for each participant who have signed the ICF and that has been enrolled in the study. The biobank of PIPAC trial will collect biological samples (blood and tumors) from patients included in the trial. See appendix G for more details.

13. PUBLICATION AND DISSEMINATION POLICY

The analysis of the data (short-term follow-up) are planned so the results of this study will be published collectively, the final manuscript being prepared by the Nab-PIPAC team. The Investigator Coordinator will be one of the first or last authors. Any manuscript, abstract or other publication or presentation of results or information arising from the study (including ancillary studies involving trial patients) will be prepared in conjunction with the Investigator Coordinator. Such materials will be submitted to Investigator Coordinator for review and comment at least 30 days prior to submission for publication or presentation.

14. FUNDING AND SUPPORT

14.1 Funding

Funding has been obtained from Bourse jeunes chercheurs HUG. Several applications for funding have been submitted to local foundations.

14.2 Other Support

The PIPAC device will be provided by each implicated center Surgery Department.

15. INSURANCE

In accordance with ICH-GCP, the Sponsor, HUG, will provide a study specific insurance (certificate as a separate study related document) covering both sites in Switzerland (Lausanne and Geneva).

16. REFERENCES

1. Dedrick RL, Myers CE, Bungay PM, DeVita VT, Jr. Pharmacokinetic rationale for peritoneal drug administration in the treatment of ovarian cancer. Cancer Treat Rep. 1978;62(1):1-11.
2. Alberts DS, Liu PY, Hannigan EV, O'Toole R, Williams SD, Young JA, et al. Intraperitoneal cisplatin plus intravenous cyclophosphamide versus intravenous cisplatin plus intravenous cyclophosphamide for stage III ovarian cancer. The New England journal of medicine. 1996;335(26):1950-5.
3. Armstrong DK, Bundy B, Wenzel L, Huang HQ, Baergen R, Lele S, et al. Intraperitoneal cisplatin and paclitaxel in ovarian cancer. The New England journal of medicine. 2006;354(1):34-43.
4. Gadducci A, Carnino F, Chiara S, Brunetti I, Tanganelli L, Romanini A, et al. Intraperitoneal versus intravenous cisplatin in combination with intravenous cyclophosphamide and epidoxorubicin in optimally cytoreduced advanced epithelial ovarian cancer: a randomized trial of the Gruppo Oncologico Nord-Ovest. Gynecologic oncology. 2000;76(2):157-62.
5. Kirmani S, Braly PS, McClay EF, Saltzstein SL, Plaxe SC, Kim S, et al. A comparison of intravenous versus intraperitoneal chemotherapy for the initial treatment of ovarian cancer. Gynecologic oncology. 1994;54(3):338-44.
6. Markman M, Bundy BN, Alberts DS, Fowler JM, Clark-Pearson DL, Carson LF, et al. Phase III trial of standard-dose intravenous cisplatin plus paclitaxel versus moderately high-dose carboplatin followed by intravenous paclitaxel and intraperitoneal cisplatin in small-volume stage III ovarian carcinoma: an intergroup study of the Gynecologic Oncology Group, Southwestern Oncology Group, and Eastern Cooperative Oncology Group. J Clin Oncol. 2001;19(4):1001-7.
7. Polyzos A, Tsavaris N, Kosmas C, Giannikos L, Katsikas M, Kalahanis N, et al. A comparative study of intraperitoneal carboplatin versus intravenous carboplatin with intravenous cyclophosphamide in both arms as initial chemotherapy for stage III ovarian cancer. Oncology. 1999;56(4):291-6.
8. Yen MS, Juang CM, Lai CR, Chao GC, Ng HT, Yuan CC. Intraperitoneal cisplatin-based chemotherapy vs. intravenous cisplatin-based chemotherapy for stage III optimally cytoreduced epithelial ovarian cancer. Int J Gynaecol Obstet. 2001;72(1):55-60.
9. Huo YR, Richards A, Liauw W, Morris DL. Hyperthermic intraperitoneal chemotherapy (HIPEC) and cytoreductive surgery (CRS) in ovarian cancer: A systematic review and meta-analysis. European journal of surgical oncology : the journal of the European Society of Surgical Oncology and the British Association of Surgical Oncology. 2015;41(12):1578-89.
10. Jaaback K, Johnson N, Lawrie TA. Intraperitoneal chemotherapy for the initial management of primary epithelial ovarian cancer. Cochrane Database Syst Rev. 2016(1):CD005340.
11. Dakwar GR, Shariati M, Willaert W, Ceelen W, De Smedt SC, Remaut K. Nanomedicine-based intraperitoneal therapy for the treatment of peritoneal carcinomatosis - Mission possible? Adv Drug Deliv Rev. 2016.
12. Goodman MD, McPartland S, Detelich D, Saif MW. Chemotherapy for intraperitoneal use: a review of hyperthermic intraperitoneal chemotherapy and early post-operative intraperitoneal chemotherapy. J Gastrointest Oncol. 2016;7(1):45-57.
13. van Driel WJ, Koole SN, Sikorska K, Schagen van Leeuwen JH, Schreuder HWR, Hermans RHM, et al. Hyperthermic Intraperitoneal Chemotherapy in Ovarian Cancer. The New England journal of medicine. 2018;378(3):230-40.

14. Glehen O, Gilly FN, Boutitie F, Bereder JM, Quenet F, Sideris L, et al. Toward curative treatment of peritoneal carcinomatosis from nonovarian origin by cytoreductive surgery combined with perioperative intraperitoneal chemotherapy: a multi-institutional study of 1,290 patients. Cancer. 2010;116(24):5608-18.
15. Jacquet P, Stuart OA, Chang D, Sugarbaker PH. Effects of intra-abdominal pressure on pharmacokinetics and tissue distribution of doxorubicin after intraperitoneal administration. Anticancer Drugs. 1996;7(5):596-603.
16. Reymond MA, Hu B, Garcia A, Reck T, Kockerling F, Hess J, et al. Feasibility of therapeutic pneumoperitoneum in a large animal model using a microvaporisator. Surg Endosc. 2000;14(1):51-5.
17. Esquis P, Consolo D, Magnin G, Pointaire P, Moretto P, Ynsa MD, et al. High intra-abdominal pressure enhances the penetration and antitumor effect of intraperitoneal cisplatin on experimental peritoneal carcinomatosis. Annals of surgery. 2006;244(1):106-12.
18. Minchinton AI, Tannock IF. Drug penetration in solid tumours. Nat Rev Cancer. 2006;6(8):583-92.
19. Facy O, Al Samman S, Magnin G, Ghiringhelli F, Ladoire S, Chauffert B, et al. High pressure enhances the effect of hyperthermia in intraperitoneal chemotherapy with oxaliplatin: an experimental study. Annals of surgery. 2012;256(6):1084-8.
20. Solass W, Hetzel A, Nadiradze G, Sagynaliev E, Reymond MA. Description of a novel approach for intraperitoneal drug delivery and the related device. Surg Endosc. 2012;26(7):1849-55.
21. Solass W, Herbette A, Schwarz T, Hetzel A, Sun JS, Dutreix M, et al. Therapeutic approach of human peritoneal carcinomatosis with Dbait in combination with capnoperitoneum: proof of concept. Surg Endosc. 2012;26(3):847-52.
22. Reymond M. Pleura and Peritoneum Retrieved 18 Jul. 2018.
23. Kuchen N, Cerser T, Hailemariam S and Scoeb O. Safety and efficacy of pressurized intraperitoneal/intrathoracic aerosol chemotherapy (PIPAC/PITAC) in patients with peritoneal and/or pleural carcinomatosis: A preliminary experience. J Med Therap. 2018.
24. Hubner M, Grass F, Teixeira-Farinha H, Pache B, Mathevet P, Demartines N. Pressurized IntraPeritoneal Aerosol Chemotherapy - Practical aspects. European journal of surgical oncology : the journal of the European Society of Surgical Oncology and the British Association of Surgical Oncology. 2017;43(6):1102-9.
25. Hubner M, Teixeira Farinha H, Grass F, Wolfer A, Mathevet P, Hahnloser D, et al. Feasibility and Safety of Pressurized Intraperitoneal Aerosol Chemotherapy for Peritoneal Carcinomatosis: A Retrospective Cohort Study. Gastroenterology research and practice. 2017;2017:6852749.
26. Teixeira Farinha H, Grass F, Labgaa I, Pache B, Demartines N, Hubner M. Inflammatory Response and Toxicity After Pressurized IntraPeritoneal Aerosol Chemotherapy. Journal of Cancer. 2018;9(1):13-20.
27. Teixeira Farinha H, Grass F, Kefleyesus A, Achtari C, Romain B, Montemurro M, et al. Impact of Pressurized Intraperitoneal Aerosol Chemotherapy on Quality of Life and Symptoms in Patients with Peritoneal Carcinomatosis: A Retrospective Cohort Study. Gastroenterology research and practice. 2017;2017:4596176.
28. Grass F, Vuagniaux A, Teixeira-Farinha H, Lehmann K, Demartines N, Hubner M. Systematic review of pressurized intraperitoneal aerosol chemotherapy for the treatment of advanced peritoneal carcinomatosis. The British journal of surgery. 2017;104(6):669-78.
29. Nowacki M, Alyami M, Villeneuve L, Mercier F, Hubner M, Willaert W, et al. Multicenter comprehensive methodological and technical analysis of 832 pressurized

intraperitoneal aerosol chemotherapy (PIPAC) interventions performed in 349 patients for peritoneal carcinomatosis treatment: An international survey study. European journal of surgical oncology : the journal of the European Society of Surgical Oncology and the British Association of Surgical Oncology. 2018.
30. Tempfer CB, Giger-Pabst U, Seebacher V, Petersen M, Dogan A, Rezniczek GA. A phase I, single-arm, open-label, dose escalation study of intraperitoneal cisplatin and doxorubicin in patients with recurrent ovarian cancer and peritoneal carcinomatosis. Gynecologic oncology. 2018.
31. Schettini F, Giuliano M, De Placido S, Arpino G. Nab-paclitaxel for the treatment of triple-negative breast cancer: Rationale, clinical data and future perspectives. Cancer Treat Rev. 2016;50:129-41.
32. Desai N, Trieu V, Yao Z, Louie L, Ci S, Yang A, et al. Increased antitumor activity, intratumor paclitaxel concentrations, and endothelial cell transport of cremophor-free, albumin-bound paclitaxel, ABI-007, compared with cremophor-based paclitaxel. Clin Cancer Res. 2006;12(4):1317-24.
33. Chen N, Li Y, Ye Y, Palmisano M, Chopra R, Zhou S. Pharmacokinetics and pharmacodynamics of nab-paclitaxel in patients with solid tumors: disposition kinetics and pharmacology distinct from solvent-based paclitaxel. J Clin Pharmacol. 2014;54(10):1097-107.
34. Gardner ER, Dahut WL, Scripture CD, Jones J, Aragon-Ching JB, Desai N, et al. Randomized crossover pharmacokinetic study of solvent-based paclitaxel and nab-paclitaxel. Clin Cancer Res. 2008;14(13):4200-5.
35. Cristea MC, Rivkin S, Lim D, Chung V, Chao J, Wakabayashi M, et al. Phase I trial of intraperitoneal nab-paclitaxel in the treatment of advanced malignancies primarily confined to the peritoneal cavity. Annals of Oncology 2014;25 (suppl_4)(iv146-iv164.).
36. Paik PK, James LP, Riely GJ, Azzoli CG, Miller VA, Ng KK, et al. A phase 2 study of weekly albumin-bound paclitaxel (Abraxane(R)) given as a two-hour infusion. Cancer chemotherapy and pharmacology. 2011;68(5):1331-7.
37. Coccolini F, Acocella F, Morosi L, Brizzola S, Ghiringhelli M, Ceresoli M, et al. High Penetration of Paclitaxel in Abdominal Wall of Rabbits after Hyperthermic Intraperitoneal Administration of Nab-Paclitaxel Compared to Standard Paclitaxel Formulation. Pharmaceutical research. 2017;34(6):1180-6.
38. Mizuta N, Nakagawa T, Yamamoto K, Nishioka T, Kume M, Makimoto H, et al. Compatibility and Stability of Nab-Paclitaxel in Combination with Other Drugs. The Kobe journal of medical sciences. 2017;63(1):E9-E16.
39. Takaishi K, Komohara Y, Tashiro H, Ohtake H, Nakagawa T, Katabuchi H, et al. Involvement of M2-polarized macrophages in the ascites from advanced epithelial ovarian carcinoma in tumor progression via Stat3 activation. Cancer Sci. 2010;101(10):2128-36.
40. Zhang M, He Y, Sun X, Li Q, Wang W, Zhao A, et al. A high M1/M2 ratio of tumor-associated macrophages is associated with extended survival in ovarian cancer patients. J Ovarian Res. 2014;7:19.
41. Cullis J, Siolas D, Avanzi A, Barui S, Maitra A, Bar-Sagi D. Macropinocytosis of Nab-paclitaxel Drives Macrophage Activation in Pancreatic Cancer. Cancer immunology research. 2017;5(3):182-90.
42. Soliman HH. nab-Paclitaxel as a potential partner with checkpoint inhibitors in solid tumors. Onco Targets Ther. 2017;10:101-12.
43. Adams S, Diamond J, Hamilton E. Phase IB trial of atezolizumab in combination with nab-paclitaxel in patients with metastatic triple-negative breast cancer (mTNBC). . J Clin Oncol. 2016;34:1009.

44. Camidge DR, Liu SV, Powderly J. Atezolizumab (MPDL3280A) combined with platinum-based chemotherapy in non-small cell lung cancer (NSCLC): a phase IB safety and efficacy update. . J Thorac Oncol. 2015;10:S176–S7.
45. Royal RE, Levy C, Turner K, Mathur A, Hughes M, Kammula US, et al. Phase 2 trial of single agent Ipilimumab (anti-CTLA-4) for locally advanced or metastatic pancreatic adenocarcinoma. Journal of immunotherapy. 2010;33(8):828-33.
46. Brahmer JR, Tykodi SS, Chow LQ, Hwu WJ, Topalian SL, Hwu P, et al. Safety and activity of anti-PD-L1 antibody in patients with advanced cancer. The New England journal of medicine. 2012;366(26):2455-65.
47. Winograd R, Byrne KT, Evans RA, Odorizzi PM, Meyer AR, Bajor DL, et al. Induction of T-cell Immunity Overcomes Complete Resistance to PD-1 and CTLA-4 Blockade and Improves Survival in Pancreatic Carcinoma. Cancer immunology research. 2015;3(4):399-411.
48. Le DT, Lutz E, Uram JN, Sugar EA, Onners B, Solt S, et al. Evaluation of ipilimumab in combination with allogeneic pancreatic tumor cells transfected with a GM-CSF gene in previously treated pancreatic cancer. Journal of immunotherapy. 2013;36(7):382-9.
49. Solass W, Kerb R, Murdter T, Giger-Pabst U, Strumberg D, Tempfer C, et al. Intraperitoneal chemotherapy of peritoneal carcinomatosis using pressurized aerosol as an alternative to liquid solution: first evidence for efficacy. Annals of surgical oncology. 2014;21(2):553-9.
50. Blanco A, Giger-Pabst U, Solass W, Zieren J, Reymond MA. Renal and hepatic toxicities after pressurized intraperitoneal aerosol chemotherapy (PIPAC). Annals of surgical oncology. 2013;20(7):2311-6.
51. Solass W, Giger-Pabst U, Zieren J, Reymond MA. Pressurized intraperitoneal aerosol chemotherapy (PIPAC): occupational health and safety aspects. Annals of surgical oncology. 2013;20(11):3504-11.
52. Robella M, Vaira M, De Simone M. Safety and feasibility of pressurized intraperitoneal aerosol chemotherapy (PIPAC) associated with systemic chemotherapy: an innovative approach to treat peritoneal carcinomatosis. World J Surg Oncol. 2016;14:128.
53. Tempfer CB, Celik I, Solass W, Buerkle B, Pabst UG, Zieren J, et al. Activity of Pressurized Intraperitoneal Aerosol Chemotherapy (PIPAC) with cisplatin and doxorubicin in women with recurrent, platinum-resistant ovarian cancer: preliminary clinical experience. Gynecologic oncology. 2014;132(2):307-11.
54. Tempfer CB, Winnekendonk G, Solass W, Horvat R, Giger-Pabst U, Zieren J, et al. Pressurized intraperitoneal aerosol chemotherapy in women with recurrent ovarian cancer: A phase 2 study. Gynecologic oncology. 2015;137(2):223-8.
55. Demtroder C, Solass W, Zieren J, Strumberg D, Giger-Pabst U, Reymond MA. Pressurized intraperitoneal aerosol chemotherapy with oxaliplatin in colorectal peritoneal metastasis. Colorectal Dis. 2016;18(4):364-71.
56. Nadiradze G, Giger-Pabst U, Zieren J, Strumberg D, Solass W, Reymond MA. Pressurized Intraperitoneal Aerosol Chemotherapy (PIPAC) with Low-Dose Cisplatin and Doxorubicin in Gastric Peritoneal Metastasis. J Gastrointest Surg. 2016;20(2):367-73.
57. von Minckwitz G, Martin M, Wilson G, Alba E, Schmidt M, Biganzoli L, et al. Optimizing taxane use in MBC in the emerging era of targeted chemotherapy. Crit Rev Oncol Hematol. 2013;85(3):315-31.
58. Yamaguchi T, Fushida S, Yamamoto Y, Tsukada T, Kinoshita J, Oyama K, et al. Tumor-associated macrophages of the M2 phenotype contribute to progression in gastric cancer with peritoneal dissemination. Gastric Cancer. 2016;19(4):1052-65.

59. Mielgo A, Schmid MC. Impact of tumour associated macrophages in pancreatic cancer. BMB Rep. 2013;46(3):131-8.
60. Nathan MR, Schmid P. The emerging world of breast cancer immunotherapy. Breast. 2017.
61. (NCCN) NCCN. NCCN clinical practice guidelines in oncology: Genetic/familial high-risk assessment breast and ovarian, . Version 1.20:[Available from: www.nccn.org.
62. Le Tourneau C, Gan HK, Razak AR, Paoletti X. Efficiency of new dose escalation designs in dose-finding phase I trials of molecularly targeted agents. PloS one. 2012;7(12):e51039.
63. Dindo D, Demartines N, Clavien PA. Classification of surgical complications: a new proposal with evaluation in a cohort of 6336 patients and results of a survey. Annals of surgery. 2004;240(2):205-13.
64. Chamokova B, Ciolina M, Pichi A, Iannitti M, Baldassari P, Cavallini C, et al. Imaging of Peritoneal Carcinomatosis: A prospective study to define correlation between surgical and radiological Peritoneal Cancer Index in patients before HIPEC and Peritonectomy. ECR2015.
65. Harmon RL, Sugarbaker PH. Prognostic indicators in peritoneal carcinomatosis from gastrointestinal cancer. International seminars in surgical oncology : ISSO. 2005;2(1):3.
66. Fagotti A, Ferrandina G, Fanfani F, Ercoli A, Lorusso D, Rossi M, et al. A laparoscopy-based score to predict surgical outcome in patients with advanced ovarian carcinoma : a pilot study. Annals of surgical oncology. 2006;13(8):1156-61.
67. Solass W, Sempoux C, Detlefsen S, Carr JN, Bibeau F. Peritoneal sampling and histological assessment of therapeutic response in peritoneal metastasis: proposal of the Peritoneal Regression Grading Score (PRGS). Pleura and Peritoneum. 2016;1(2):99–107.
68. Buscone S, Argentieri MC, Pilla D, Cattoretti G. Whole-slide, quadruple immunofluorescence labeling of routinely processed paraffin sections. Applied immunohistochemistry & molecular morphology : AIMM. 2014;22(4):e1-7.
69. Gendusa R, Scalia CR, Buscone S, Cattoretti G. Elution of High-affinity (>10-9 KD) Antibodies from Tissue Sections: Clues to the Molecular Mechanism and Use in Sequential Immunostaining. The journal of histochemistry and cytochemistry : official journal of the Histochemistry Society. 2014;62(7):519-31.
70. Hübner M, Teixeira H, Boussaha T, Cachemaille M, Lehmann K, Demartines N. PIPAC. Rev Med Suisse 2015; volume 11. 1325-1330
71. Alyami M, Hübner M, Grass F, Bakrin N, Villeneuve L, Laplace N, Passot G, Glehen O, Kepenekian V : Pressureised Intraperitoneal Aerosol Chemotherapy : rational, evidence, and potential indications. Lancet Oncol 2019; 20: e368–77

17. APPENDICES

A. SAFETY ASSESSMENT

- **COMMON TERMINOLOGY CRITERIA FOR ADVERSE EVENTS V5.0 (CTCAE)**

The CTCAE version 5.0 commonly known as CTC, was published November 27, 2017 by Division of Cancer Treatment and Diagnosis at National Cancer Institute of the National Institutes of Health and United States Department of Health and Human Services. Please consult the website of the Cancer Therapy Evaluation Program: www.ctep.cancer.gov.
The CTC v5.0 is descriptive terminology which can be utilized for adverse event reporting. A grading (severity) scale is provided for each AE term.

- **Clavien-Dindo classification and comprehensive complication index (CCI)(64)**

The therapy used to correct a specific complication is the basis of this classification in order to rank a complication in an objective and reproducible manner. It consists of 7 grades (I, II, IIIa, IIIb, IVa, IVb and V). The introduction of the subclasses a and b allows a contraction of the classification into 5 grades (I, II, III, IV and V) depending on the size of the population observed or the focus of a study.

Grades	Definition
Grade I	Any deviation from the normal postoperative course without the need for pharmacological treatment or surgical, endoscopic and radiological interventions<br>Allowed therapeutic regimens are: drugs as antiemetics, antipyretics, analgetics, diuretics and electrolytes and physiotherapy. This grade also includes wound infections opened at the bedside.
Grade II	Requiring pharmacological treatment with drugs other than such allowed for grade I complications.<br>Blood transfusionsand total parenteral nutritionare also included.
Grade III	Requiring surgical, endoscopic or radiological intervention
- IIIa	Intervention not under general anesthesia
- IIIb	Intervention under general anesthesia
Grade IV	Life-threatening complication (including CNS complications)* requiring IC/ICU-management
- IVa	single organ dysfunction (including dialysis)
- IVb	multiorgandysfunction
Greade V	Death of a patient
Suffix "d"	If the patients suffers from a complication at the time of discharge, the suffix "d" (for 'disability") is added to the respective grade of complication. This label indicates the need for a follow-up to fully evaluate the complication.

**brain hemorrhage, ischemic stroke, subarrachnoidalbleeding,but excluding transient ischemic attacks (TIA);IC: Intermediate care; ICU: Intensive care unit.*

B. RADIOLOGICAL ASSESSMENT
Revised RECIST Criteria version 1.1 CT-PCI (65)

Radiological analysis procedure:
Two transverse planes and two sagittal planes divide the abdomen into 9 regions. The upper transverse plane is located at the lowest aspect of the costal margin and the lower transverse plane is placed at the anterior superior iliac spine. The sagittal planes divide the abdomen into three equal sectors. The lines define the nine regions which are numbered in a clockwise direction with 0 at the umbilicus and 1 defining the space beneath the right hemidiaphragm. Regions 9-12 divide the small bowel. Lesion size score is determined after complete lysis of all adhesions and the complete inspection of all parietal and visceral peritoneal surfaces. It refers to the greatest diameter of tumor implants that are distributed on the peritoneal surfaces. Primary tumors or localized recurrences at the primary site that can be removed definitively are excluded from the lesion size assessment. If there is confluence of disease matting abdominal or pelvic structures together, this is automatically scored as L-3 even if it is a thin confluence of cancerous implants.

C. SURGICAL PROCEDURE: PERITONEAL CARCINOMATOSIS INDEX (PCI), FAGOTTI SCORE AND SURGICAL PROCEDURE FOR BIOPSIES

PCI score:(66) The peritoneal cancer index (PCI) is used to assess the extent of peritoneal cancer throughout the peritoneal cavity. For this purpose, the peritoneal cavity is divided in 13 well-defined regions (see figure). In each of the 13 regions, the size of the largest tumor nodule is measured. If no tumor is visualized, a score of “0” is given to that region. If the largest tumor nodule is smaller than 0.5 cm, the score is “1”. For tumors measuring between 0.5 cm and 5 cm, the score is “2”. For lesions larger than 5 cm, the score is “3”. If there is layering or a confluence of multiple small tumor nodules, the score is “3”. The PCI is calculated by adding the scores of all 13 regions together with a maximum score of 39 (13×3).

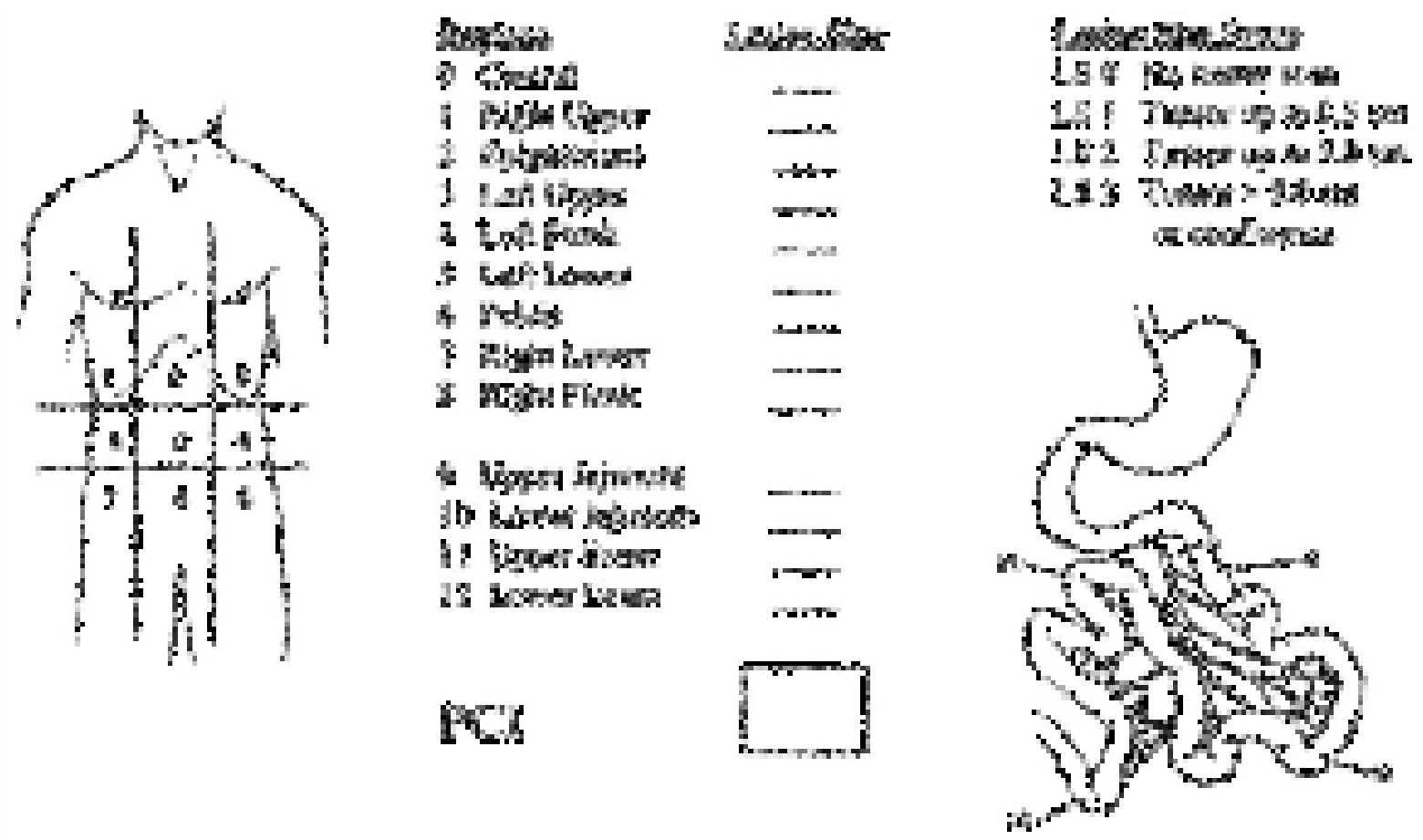

Fagotti score (only for ovarian carcinoma): (67)

- Omental cake
- Peritoneal carcinomatosis
- Diaphragmatic carcinomatosis
- Mesenteric retraction
- Stomach infiltration
- Liver metastases

Each parameter was attributed a score of 0 to 2 with a maximum of 18 (6x2).
Cytoreduction is incomplete in 100% of patients with a score ≥ 8.

Surgical Procedure for peritoneal biopsies

- During laparotomy or laparoscopy, the peritoneal cavity should be explored thoroughly and the Peritoneal Carcinomatosis Index (PCI) and Fagotti score in case of ovarian carcinoma [18] should be documented. Comment: placement of the camera access port in the abdominal midline might be advantageous [19]. The staging procedure should be videorecorded to allow later comparisons, or documented with intraoperative pictures during open surgery.
- 5 punch biopsies should be taken at suspect localizations, 3 for the PRGS analysis and 2 for the translational reasearch (typically in the right upper quadrant, right lower quadrant, left upper quadrant and left under quadrant), typically from tumor nodules. Comment: usually, biopsies are taken from the parietal peritoneum. In the presence of adherences, it might be impossible to obtain samples from each quadrant. Biopsies from the visceral peritoneum might induce a risk of postoperative hollow organ perforation. Using narrow-band imaging and image enhancement software might increase accuracy of biopsies [20].
- Peritoneal biopsies should have a diameter of at least 3 mm, ideally 5 mm. The use of a

punch biopsy device is recommended to generate standardized samples. Comment: the sample morphology is decisive for proper histopathological analysis. The biopsy should contain both the mesothelial and the submesothelial layers. Peritoneal samples are directional and the relationship surface/depth can influence results.

- Additionally, one local peritonectomy of several cm^2 should be taken. Comment: larger samples are needed in order to increase the accuracy of negative (tumor-free) biopsies for documenting complete tumor regression.
- Representative samples should be taken from surgical specimen. Comment: in the case of cytoreductive surgery, representative samples should be taken from each resected organ. The analysis of all tumor nodules is not feasible in clinical routine and is not required to assess diagnosis, extent and regression grading. Only appropriate selection is mandatory.
- Research samples should be obtained from the biopsies above. In any case the presence or absence of tumor has to be validated by a pathologist and the preservation of sufficient tumor tissue for routine diagnosis and assessment of PRGS has the absolute priority. Whether to divide the research samples into two parts and to send half of the sample to the routine diagnostic laboratory and the other half to the research laboratory/biobank or to send all samples directly to the diagnostic laboratory and then to decide which sample to use for research, is an individual decision for each institution.
- In the cases a negative peritoneal histology is suspected, a peritoneal cytology is recommended. Comment: after induction therapy or palliative chemotherapy, no vital tumor cells might be documented in the peritoneal biopsies and in the local peritonectomy sample. In this case, another three-step sections is recommended to confirm complete response. In the presence of tumor scarring or in the absence of macroscopic peritoneal lesions, sampling of peritoneal fluid for cytological analysis is advisable.

D. HISTOPATHOLOGICAL ANALYSIS

Histopathological analysis of peritoneal samplings (PRGS score):

Recommendations for peritoneal sampling

A peritoneal cytology should be sampled at the beginning of each procedure and peritoneal punch biopsies should be performed with a diameter of 3 to 5mm in all four abdominal quadrants. 3 biopsies should be collected at suspect localizations (in three different quadrants when feasible), typically from tumor nodules. Usually, biopsies are taken from the parietal peritoneum. In the presence of adherences, it might be impossible to obtain samples from each quadrant. Biopsies from the visceral peritoneum might induce a risk of postoperative hollow organ perforation. Additionally, one local peritonectomy of several square centimeters should be taken in order to increase the accuracy of negative (tumor-free) biopsies for documenting complete tumor regression. In the presence of tumor scarring or in the absence of macroscopic peritoneal lesions, sampling of peritoneal fluid for cytological analysis is advisable.

The sample morphology is decisive for proper histopathological analysis. The biopsy should contain both the mesothelial and the submesothelial layers. Peritoneal samples are directional and the relationship surface/depth can influence results, in particular for quantitative pharmacological analyses.

Recommendations for fixation and staining

For sample fixation, the use of 10% buffered formalin is recommended for a 24–48 hours' period of time. Sample fixation should be appropriate for reliable histopathological analysis and tumor regression grading. Optimal fixation is mandatory to preserve antigenicity for immunohistochemistry and DNA integrity for molecular studies. Adequate embedding is needed in order to perform a reliable histopathological analysis. This step is important for immunohistochemistry and molecular testing.

For PRGS assessment, hematoxylin-eosin staining is performed according to usual protocols.

Peritoneal Regression Grading Score (PRGS) assessment (68), defined as:

- Grade 1: complete response (absence of tumor cells)
- Grade 2: major response (major regression features, few residual tumor cells)
- Grade 3: minor response (some regressive features but predominance of residual tumor cells)
- Grade 4: no response (tumor cells without any regressive features).

Acellular mucin and infarct-like necrosis should be regarded as regression features. We recommend **reporting the mean and the worst value** of the regression grades obtained. In cases where the Peritoneal Regression Grading Score cannot be determined, PM should be classified as PRGSx, for example if enteroparietal adhesions prevent access to the abdomen or in the absence of representative tumor material.

E. DRUG INTERACTIONS (CYP2C8, 3A4)

References: HUG local guidelines www.pharmacoclin.ch (> outils > carte dynamique des interactions médicamenteuses et CYP) **and** **www.uptodate.com**. The current version is annexed (see chapter 18).

F. PHARMACOKINETIC ANALYSIS

Blood samples for nab-paclitaxel plasmatic concentrations analysis will be collected by the delegate of each phase I Unit (CHUV/HUG) in the surgical department (CHUV/HUG). The following points will be collected:

Free plasmatic concentration of Nab-Paclitaxel: one blood sample of 5-7 mL EDTA at Pre-dose, end of infusion, H1, H4 and 24 hours after the first cycle of PIPAC for the two first patients treated for each dose level escalation.

Samples will be processed following the procedure indiquated.

Blood samples (EDTA, 6 ml) will be collected at 5 time points: Pre-dose, end of infusion, H1, H4 and 24 hours after the first cycle of PIPAC for the two first patients treated for each dose level escalation. For each blood sample, the exact (real) time of collection should be reported in the PK eCRF.

Blood samples collected at Pre-dose, end of infusion, H1 and H4 should be stored upon collection at +4°C. This batch of samples will be send without delay to the local hospital laboratory (i.e. at CHUV, Laboratory of Clinical Pharmacology, PCL8; at UNIGE, Laboratory of Dr Labidi-Galy, CMU Room A06.2910) for centrifugation (2000g for 10 min at +4°C), and the collected plasma samples (2 x *ca* 1 ml-aliquots) are stored on-site preferably in cryovials at -80°C (if not possible -20°C) in a dedicated box “PIPAC Nab-paclitaxel”.

Blood sample collected at H24 shall be send without delay to the local hospital laboratory (i.e. at CHUV, Laboratory of Clinical Pharmacology, PCL8; at UNIGE, Laboratory of Dr Labidi-Galy, CMU Room A06.2910) for centrifugation (2000g for 10 min at +4°C), and the collected plasma samples (2 x *ca* 1 ml-aliquots) are stored on-site preferably in cryovials at -80°C (if not possible -20°C) in a dedicated box “PIPAC Nab-paclitaxel”.

For HUG, frozen plasma sample shall be shipped (HUG->CHUV shuttle) to PCL8 on dry ice, in a securely closed styrofoam box batchwise (patient by patient) after the first treatment administration, along with the information required for pharmacokinetic interpretations (copy of a dedicated drug level monitoring form (copy attached)) via the *Réception Centralisée des Laboratoires* du CHUV (RL, BH18) to:

Laboratory of Clinical Pharmacology, Att. Prof L.A. Decosterd, BU19 -04 labo 713, Centre Hospitalier Universitaire Vaudois CH 1011 Lausanne.
Email : laurentarthur.decosterd@chuv.ch, tél : 021 314 4272 ou 4271.

G. TRANSLATIONAL RESEARCH ANALYSIS

The translational research samples will be stored for Biobank storage for each participant who have signed the ICF and that has been enrolled in the study. The biobank of PIPAC trial will collect biological samples (blood and tumors) from patients included in the trial.

At CHUV, samples for translational research (2 punch biopsies) will be collected and stored in the oncologic biobank of the CHUV. Then they will be sent to the laboratory of Dr Intidhar Labidi-Galy(A06.2910.a Faculty of Medicine, Geneva).
At HUG, samples for translational research (2 punch biopsies) will be sent to the Labidi-Galy lab. All the translational research samples will be stored in the biobank of the laboratory of Dr Intidhar Labidi-Galy (A06.2910.a Faculty of Medicine, Geneva) where the analysis of the microenvironement by immunochemistry will be performed.

Blood samples 3ml dedicated to analysis of Whole Exome Sequencing from the CHUV and HUG will be centralized and stored in -80°C freezers in the laboratory of Dr Intidhar Labidi-Galy (Room A06.2910.a, Faculty of Medicine, UNIGE). Cooling and temperature control are monitored 24 hours by the Faculty of Medicine staff. Access to this area is restricted by use of personal ID to access to the building and the rooms. DNA extraction will be processed in the laboratory of Dr Intidhar Labidi-Galy and sent to the Genome Center of UNIGE (Campus Biotech) where the analysis will be performed.

- **Multisplexed immunochemistry**

  We will perform sequential multiplexed immunochemistry (IHC) of samples obtained before treatment (D1, cycle 1) and during treatment (D1, cycle 3). Assessment of the presence of TAMs ($CD68^+$, $CD163^+$, $Tie2^+$), regulatory T cells ($Foxp3^+$), TILs ($CD8^+$), plasmacytoid dendritic cells ($BDCA2^+$), resident memory T cells ($CD103^+$), PD-L1/ PD1 and other immune cells to be defined. The analysis will be performed in the the laboratory of Dr Intidhar Labidi-Galy. FFPE tissue sections will be cut at 4 µm, dried overnight, dewaxed by immersion in xylene, rehydrated in ethanol of decreasing concentrations, stained by Harris hematoxylin for 1 min, and coated by a glass coverslip with an aqueous mounting solution. Tissue sections will be scanned into MRXS images using Pannoramic 250 Flash III scanner (3D Histech, Budapest, Hungary). Glass coverslips will be removed by immersion in hot water, and slides were subjected to heat-mediated antigen retrieval in pH6 citrate buffer for 10 min. Endogenous peroxidases, non-specific proteins, endogenous biotins and avidins will be blocked with corresponding blocking solutions from Dako®. The first primary antibody will be applied to tissue sections, followed by a biotinylated-secondary antibody and a streptavidin-HRP complex, and revealed by AEC chromogen. Tissue sections will be scanned and AEC staining was removed by immersion in ethanol of increasing concentrations. Antibodies will be stripped by boiling tissue sections in pH6 citrate buffer for 10 to 20 min, and putative residual antibodies were blocked by Fab fragments directed against the host of the previous primary antibody used. Multiplex IHC consists of sequential cycles of staining with primary antibodies revealed by AEC chromogen, tissue section scanning, removal of AEC chromogen with ethanol, antibody stripping and blocking with Fab fragments. MRXS scans will be converted into TIFF files at half of the original resolution. A Matlab algorithm is written to execute all the following steps. Positive control tissues will be cropped from the original slide image. For each tumor, images stainings will be aligned to the image of hematoxylin staining. ROIs were defined and processed to extract the positive staining (revealed by AEC chromogen) from

each marker. $CD8^+$ staining or $CD68^+$ staining for instance will be superimposed to $Cytokeratin^+$ staining for each tumor's section in order to allow the visualization of final multiplex IHC images.

.

- **Molecular Biology Analysis**

We plan to conduct DNA and RNA sequencing of samples obtained before and during treatment. The analysis will be delegate to the Fondation Campus Biotech Geneva (FCBG).

- **Gene expression profile (RNAseq):** quantification of gene expression by exome-capture RNAseq will be performed on FFPE tumor samples obtained before (cycle 1, D1) and during treatment (cycle 2 and 3, D1). Bioinformatic processing will be based on a standardised pipeline (bwa, edgeR). The downstream analysis includes unsupervised hierarchical clustering for the discovery of underlying subgroups, differential expression analysis of matched samples before and during treatment and differential expression analysis between responders and non-responders. Gene expression information can also be used to deconvolute immune infiltrates (for example, CIBERSORT, TIMER), supplementing the immunohistochemical estimates.
- **Whole-exome sequencing:** Whole-exome sequencing will be performed on blood samples 3ml obtained before (C1D0) and FFPE tumor samples processed on a standardized pipeline (bcbio) for the identification of pathogenic variants (mutations) and copy number alterations (CNVkit). Mutational signatures can also be derived from exome data and have been associated with distinct biological processes, such as deficient DNA repair. We plan to compare the patterns of responders with non-responders in the hope of identifying candidate biomarkers. In addition, we will examine the changes that might have resulted from exposure to treatment, such as the expansion of potentially resistant clones.

Fondation Campus Biotech Geneva (FCBG) 9 Chemin des Mines
1202 Genève

H. VITAL SIGNS AND CLINICAL LABORATORY TESTS

The following Vital Signs will be assessed at each visit:

- Body height (only at screening)
- Body weight
- Body temperature
- Sitting pulse
- Blood pressure
- Abdominal circumference (Only at Screening, C2D0, C3D0 and EoT visit)

The laboratory analysis detailed in the following table will be performed by the local laboratory.

Laboratory assessments	Parameters

**Hematology**	Platelet Count<br>RBC Count<br>Hemoglobin<br>Hematocrit	White blood cell count with differential :<br>Neutrophils<br>Lymphocytes<br>Monocytes<br>Eosinophils<br>Basophils		
**Chemistry**	Glucose *	Sodium	Potassium	Calcium
	Creatinine	Total bilirubin	AST	ALT
	Alk phosphatase	GGT	Total protein *	Albumin
	Urea	LDH *	C-react protein	
**Coagulation**	PT	aPTT	fibrinogen	
**Serological tumor marker**	CA 125	CEA	CA19-9	
**Urine analysis (dipstick)**	pH	protein	glucose	blood
**Pregnancy**	Urinary or Serum pregnancy test (hCG)			

* To be performed **only at Screening** visit

I. STUDY CONTACT LIST

1.3 Statistician

Dr. Petros Tsantoulis, MD, PhD
Consultant in Molecular Oncology and Biostatistic
Petros.Tsantoulis@huge.ch

Oncology Departement,
Geneva University Hospitals and medical school (HUG)

1.4 Local Site Investigators

Dr. Med Frédéric Ris, PD
Consultant Surgeon
Head of colorectal surgery and HIPEC programm
Frederic.Ris@hcuge.ch

Surgery Department, Division of visceral surgery,
Geneva University Hospitals and medical school (HUG)

Dr. Manuela Undurraga Malinverno, MD
Consultant Surgeon
Manuela.Undurraga@hcuge.ch

Gynecology Department, Onco-gynecology unit,
Geneva University Hospitals and medical school (HUG)

Co-Investigators

Dr. Noémie Lang, MD
Consultant Oncologist

Oncology Departement
Geneva University Hospitals and medical school (HUG)

Prof. Patrick Petignat, MD
Consultant Surgeon
Head of Department of Gynecology

Geneva University Hospitals and medical school (HUG)

Prof. Nicolas Demartines, MD
Consultant Surgeon
Head of Department of Visceral Surgery

Lausanne University Hospital and medical school (CHUV)

Dr. Antonia Digklia, MD
Consultant Oncologist

Oncology Departement
Lausanne University Hospital and medical school (CHUV)

Prof. Patrice Mathevet, MD

Gynecology Departement
Lausanne University Hospital and medical school (CHUV)

Dr. Apostolos Sarivalasis, MD

Oncology Departement
Lausanne University Hospital and medical school (CHUV)

Dr. Martin Hübner, PD
Consultant Surgeon
Martin.Hubner@chuv.ch

Surgery Department, Service of visceral surgery
Lausanne University Hospital and medical school (CHUV)

Pathological team:

Dr. Giacomo Puppa, MD
Consultant Pathologist

Department of Pathology and Immunology
Geneva University Hospitals and medical school (HUG), CMU

Dr. Jean Christophe Tille, MD, PhD
Consultant Pathologist

Department of Pathology and Immunology
Geneva University Hospitals and medical school (HUG), CMU

Prof. Christine Sempoux, MD, PhD
Consultant Pathologist

Pathologic Department
Lausanne University Hospital and medical school (CHUV)

Dr. Nathalie Piazzon, PhD
Consultant Pathologist

Pathologic Department
Lausanne University Hospital and medical school (CHUV)

Data management team:

Clinical Investigational Unit (UIC)

Geneva Hospital Clinical Research Center (CRC)-4AL
Rue Gabrielle Perret-Gentil, 4
CH-1205 Geneva

1.5 Monitoring institution

Clinical Investigational Unit (UIC)

Geneva Hospital Clinical Research Center (CRC)-4AL
Rue Gabrielle Perret-Gentil, 4
CH-1205 Geneva

ANNEXES

PIPAC safety-check-list

Carte dynamique des interactions médicamenteuses et CYP – HUG

Note: Study questionnaires and scales, IBs of medicinal products, PIPAC device informations and CE marking are provided as a separate documentation.
