# Supplementary material for: Safety and efficacy of cisplatin and nab-paclitaxel pressurised intraperitoneal aerosol chemotherapy (PIPAC) in patients with peritoneal carcinomatosis: Nab-PIPAC phase 1b study
Source: eBioMedicine. 2026 Sep 11;132:106477. doi: 10.1016/j.ebiom.2026.106477 (PMC13587052; doi:10.1016/j.ebiom.2026.106477)
Supplement: Supplementary file 3 [file mmc1.docx]

**Supplementary Table 1: Radiological outcomes**

|  | No intraperitoneal PD | Intraperitoneal PD |
| --- | --- | --- |
| **No extraperitoneal PD** | 0 (0%) | 4 (22%) |
| **Extraperitoneal PD** | 1 (6%) | 13 (72%) |
